# Mapping the scientific knowledge and approaches to defining and measuring hate crime, hate speech, and hate incidents: A systematic review

**DOI:** 10.1002/cl2.1397

**Published:** 2024-04-28

**Authors:** Matteo Vergani, Barbara Perry, Joshua Freilich, Steven Chermak, Ryan Scrivens, Rouven Link, Daniel Kleinsman, John Betts, Muhammad Iqbal

**Affiliations:** ^1^ Deakin University Burwood Victoria Australia; ^2^ Ontario Tech University Oshawa Ontario Canada; ^3^ John Jay College of Criminal Justice‐CUNY New York New York USA; ^4^ Michigan State University East Lansing Michigan USA; ^5^ Te Ngāpara Centre for Restorative Practice Victoria University of Wellington Kelburn Wellington New Zealand; ^6^ Department of Data Science and Artificial Intelligence, Faculty of Information Technology Monash University Clayton Victoria Australia; ^7^ Victoria University Melbourne Victoria Australia

**Keywords:** ableism, anti‐Semitism, hate conduct, hate crime, hate group, hate incident, hate propaganda, hate speech, homophobia, islamophobia, misogyny, racism, sexism, transphobia, xenophobia

## Abstract

**Background:**

The difficulties in defining hate crime, hate incidents and hate speech, and in finding a common conceptual basis constitute a key barrier toward operationalisation in research, policy and programming. Definitions disagree about issues such as the identities that should be protected, the types of behaviours that should be referred to as hateful, and how the ‘hate element’ should be assessed. The lack of solid conceptual foundations is reflected in the absence of sound data. These issues have been raised since the early 1990s (Berk, 1990; Byers & Venturelli, 1994) but they proved to be an intractable problem that continues to affect this research and policy domain.

**Objectives:**

Our systematic review has two objectives that are fundamentally connected: mapping (1) original definitions and (2) original measurement tools of hate crime, hate speech, hate incidents and surrogate terms, that is, alternative terms used for these concepts (e.g., prejudice‐motivated crime, bias crime, among many others).

**Search Methods:**

We systematically searched over 19 databases to retrieve academic and grey literature, as well as legislation. In addition, we contacted 26 country experts and searched 211 websites, as well as bibliographies of published reviews of related literature, and scrutiny of annotated bibliographies of related literature.

**Inclusion Criteria:**

This review included documents published after 1990 found in academic literature, grey literature and legislation. We included academic empirical articles with any study design, as well as theoretical articles that focused specifically on defining hate crime, hate speech, hate incidents or surrogate terms. We also reviewed current criminal or civil legislation that is intended to regulate forms of hate speech, hate incidents and hate crimes. Eligible countries included Canada, USA, UK, Ireland, Germany, France, Italy, Spain, Australia and New Zealand. For documents to be included in relation to research objective (1), they had to contain at least one original definition of hate speech, hate incidents or hate crimes, or any surrogate term. For documents to be included in relation to research objective (2), they had to contain at least one original measurement tool of hate speech, hate incidents or hate crimes, or any surrogate term. Documents could be included in relation to both research objectives.

**Data Collection and Analysis:**

The systematic search covered 1 January 1990 to 31 December 2021, with searches of academic databases conducted between 8th March and 12th April 2022 yielding 35,191 references. We carried out country‐specific searches for grey literature published in the same time period between 27th August and 2nd December 2021. These searches yielded a total of 2748 results. We coded characteristics of the definitions and measurement tools, including the protected characteristics, the approaches to categorise the ‘hate element’ and other variables. We used univariate and bivariate statistical methods for data analysis. We also carried out a social network analysis.

**Main Results:**

We provide as annex complete lists of the original definitions and measurement tools that met our inclusion criteria, for the use of researchers and policy makers worldwide. We included 423 definitions and 168 measurement tools in academic and grey literature, and 83 definitions found in legislation. To support future research and policy work in this area, we included a synthetic assessment of the (1) the operationalisability of each definition and (2) the theoretical robustness and transparency of each measurement tool. Our mapping of the definitions and measurement tools revealed numerous significant trends, clusters and differences between and within definitions and measurement tools focusing on hate crime, hate speech and hate incidents. For example, definitions and measurement tools tend to focus more on ethnic and religious identities (e.g., racism, antisemitism, Islamophobia) compared to sexual, gender and disability‐related identities. This gap is greater in the definitions and measurement tools of hate speech than hate crime. Our analysis showed geographical patterns: hate crime definitions and measurement tools are more likely to originate from Anglophonic countries, especially the USA, but hate speech definitions and measurement tools are more likely to originate from continental Europe. In terms of disciplinary fragmentation, our social network analysis revealed that the collaboration and exchange of conceptual frameworks and methodological tools between social sciences and computer science is limited, with most definitions and measurement tools clustering along disciplinary lines. More detailed findings are presented in the results section of the report.

**Authors' Conclusions:**

There is an urgent need to close the research and policy gap between the protections of ‘ethnic and religious identities’ and other (less) protected characteristics such as gender and sexual identities, age and disability. There is also an urgent need to improve the quality of methodological and reporting standards in research examining hate behaviours, including transparency in methodology and data reporting, and discussion of limitations (e.g., bias in data). Many of the measurement tools found in the academic literature were excluded because they did not report transparently how they collected and analysed the data. Further, 41% of documents presenting research on hate behaviours did not provide a definition of what they were looking at. Given the importance of this policy domain, it is vital to raise the quality and trustworthiness of research in this area. This review found that researchers in different disciplinary areas (e.g., social sciences and computer science) rarely collaborate. Future research should attempt to build on existing definitions and measurement tools (instead of duplicating efforts), and engage in more interdisciplinary collaborations. It is our hope that that this review can provide a solid foundation for researchers, government, and other bodies to build cumulative knowledge and collaboration in this important field.

## 1 PLAIN LANGUAGE SUMMARY

[A guide to definitions and measurement tools of hate crime, hate speech and hate incidents].

### 1.1 The review in brief

Our systematic review maps out how hate crime, hate speech, and hate incidents are defined and measured, highlighting gaps and suggesting improvements for research and policy. An annex is provided, comprising complete lists of the original definitions and measurement tools that met our inclusion criteria. This compendium serves as a valuable tool for researchers and policymakers globally, guiding the selection of definitions and methodologies in future efforts to understand and combat hate.

### 1.2 What is this review about?

Hate crimes, hate incidents, and hate speech are challenging to define and measure consistently. Research and policy work in this important area have developed in disciplinary silos, leading to varied definitions and measurement methods. These variations in definitions hinder how research, policies, and programmes are put into practice, resulting in inconsistent data quality and reporting. This lack of uniformity makes it difficult to build a strong evidence base needed to evaluate and create effective policies and interventions aimed at reducing hate‐related behaviours. It also creates obstacles in setting legal standards to define what constitutes hate and in determining which groups need protection. This inconsistency affects the fair treatment of victims across different jurisdictions, hinders the development of suitable laws, and leads to confusion within communities about what actually counts as hate, which can discourage people from reporting such incidents. Our review examined academic articles, grey literature, and legislation across ten countries to understand these discrepancies and their implications, including Canada, the United States, the United Kingdom, Ireland, France, Germany, Italy, Spain, Australia, and New Zealand.

### 1.3 What is the aim of this review?

This review aims to map the definitions and measurements of hate‐related phenomena, providing a resource for researchers and policymakers to build upon. We analysed definitions found in 569 documents in five languages (English, German, French, Spanish, and Italian) to understand how hate crime, hate incidents, and hate speech are defined and measured.

### 1.4 What studies are included?

We included 423 academic definitions, 168 measurement tools in the literature, and 83 definitions in legislation. The quality of evidence varies, with many studies excluded due to a lack of transparency in methodology and data reporting. The findings suggest a need for interdisciplinary collaboration and standardisation in future hate research.

### 1.5 What are the main findings of this review?

Our review found significant diversity in definitions and tools for measuring hate crimes, incidents, and speech. There is a disproportionate focus on ethnic and religious identities, with other characteristics like gender and disability less covered, especially within hate speech. Most research originates from English‐speaking countries, with limited collaboration across different disciplines. Many existing tools were excluded for opaque methodology, and a notable portion of studies lacked clear definitions, underlining the need for more rigorous standards.

### 1.6 What do the findings of this review mean?

The current landscape shows a research and policy gap in the protection of various identities and the standardisation of research methodologies. To elevate the quality and reliability of future research on hate, there is a pressing need for clearer methodologies and more comprehensive definitions that span a wider range of protected characteristics. Encouraging interdisciplinary collaboration could enhance the development of more robust research frameworks and tools.

### 1.7 How up‐to‐date is this review?

This review includes literature and legislation published between 1 January 1990, and 31 December 2021. The search for relevant studies was completed on 12 April 2022.

## 2 BACKGROUND

### 2.1 The problem, condition or issue

There is limited international consensus on how to define behaviours motivated by hate or containing a hate element. This includes hate speech, hate incidents and hate crime (Schweppe, 2021). For some, a hate crime is any criminal behaviour motivated by hate against protected identities or minority communities (Office for Democratic Institutions and Human Rights, 2009). For others, hate crime captures all malicious behaviours motivated by hate, ranging from behaviour regulated by criminal law, by civil law, or not regulated at all (Chakraborti & Garland, 2015b; Hardy, 2019). This definition overlaps with what practitioners often define as hate incidents, that is, all malicious behaviours motivated by hate that fall below the threshold of criminality (Anti‐Defamation League, 2018a, 2019). Some use the term ‘hate incident’ to capture all malicious behaviour motivated by bias, including both criminal and non‐criminal acts (Sadique et al., 2018). The definitions of hate crime and hate incidents partially overlap with the concept of hate speech, which includes verbal or non‐verbal manifestations of hatred, such as gestures, words or symbols like cross‐burnings, bestial depictions of members of minorities, hate symbols, among others (Strossen, 2018). Some of these behaviours – for example, incitement to hatred, Holocaust denial – might be regulated by criminal law in certain jurisdictions (thus overlapping with some definitions of hate crime), by civil law, or not regulated at all (thus overlapping with some definitions of hate incidents). Determining whether a crime is motivated by hate is a well‐known challenge in the literature, which led to the adoption of two classic definitional models: the ‘animus model’ and the ‘discriminatory selection model’ (Lawrence, 1999). The animus model requires that the hate element (i.e., a form of bias, prejudice or hostility) is present and visible in the crime. For example, the offender might be seen as yelling a racial slur while attacking the victim. Conversely, under the discriminatory selection model, a crime is defined as a hate crime by reason of the victim's characteristics and perceived identity. For example, selecting a victim from a minority group is sufficient to define a crime as a hate crime (Lawrence, 1999). Not all jurisdictions focus on ascertaining motives (i.e., the *mens rea* element of criminal offending). Some jurisdictions including the United Kingdom, Malta and Singapore define hate crime as being based on either hateful motive, or a demonstration of hatred, the latter being part of the *actus reus* (Walters, 2022).

Cross‐cultural research found that, at an international level, hate crime and hate speech statutes are strongly influenced by the differing social, technical, historical and cultural contexts across nations (Sheppard et al., 2021). For example, Italy has banned the display of ideas and symbols of fascism under the Law 205/1993 known as Mancino law (Campani, 2016). In the German context, the legacy of the Holocaust is mainly responsible for the criminalisation of public expressions of hate that could engender or promote violence to protected groups such as Holocaust denial and trivialisation (Bleich, 2011; Kahn, 2005). In the United States, freedom of speech is constitutionally protected and has been a central tenet of individual liberty. that has prevented the country from passing stringent laws. However, some forms of speech are criminalised in the United States, such as speech that incites imminent threat of violence (Heyman, 2009). In Canada, the Parliament amended the Criminal Code in 1970, thus rendering hate propaganda as a punishable offence (Perry & Samuels‐Wortley, 2021). These laws fall under sections 318–320 of the Criminal Code. Four specific offences are listed as hate propaganda offences or hate crimes in the Criminal Code of Canada: advocating genocide, public incitement of hatred, wilful promotion of hatred and mischief motivated by hate in relation to religious property. In addition, subparagraph 718.2(a)(i) of the Criminal Code allows for increased penalties when sentencing any criminal offence (such as assault or mischief) where there is evidence that the offence was motivated by bias, prejudice or hatred toward a particular group as listed in the code. In these instances, the crime is considered a hate crime.

The term ‘hate’ is sometimes criticised by scholars and practitioners who find it ambiguous and used normatively to criminalise political opponents (Hall, 2013). For these reasons, some scholars and practitioners use surrogate terms to refer to the concepts of hate crime, hate speech and hate incidents. For example, in Australia, hate crimes are generally referred to as *bias* crimes in New South Wales, and as *prejudice‐motivated* crimes in Victoria. Moreover, hate crimes, hate speech and hate incidents are often captured using terms like racism, antisemitism or homophobia (among others). In the literature, these community‐specific terms are used to capture attitudes and behaviours interchangeably. In some jurisdictions, there is a considerable overlap between the concept of hate crime and neighbouring concepts like ‘extremism’ and ‘terrorism’. For example, several European countries criminalise membership of extremist groups, and include these acts within their national concepts of hate crime (Perry, 2016). In the United States, domestic terrorism is often defined as hate crime (Taylor, 2019). Notwithstanding, many incidents blur the distinction between hate crime and terrorism, such as the Pittsburgh Synagogue shooting in 2018 and the Christchurch attack in 2019 (Vogel‐Scibilia, 2020).

The difficulties in defining hate behaviours and in finding a common conceptual basis constitute a key barrier toward operationalisation in research, policy, and programming. Definitions disagree about issues such as the identities that should be protected, the types of crimes that should be referred to as hate crimes, and how the ‘hate element’ should be assessed. As a consequence, when we examine data about hate crime and hate speech, we cannot be sure what hate crime and hate speech data are telling us. These concerns have existed since the early 1990s (Berk, 1990; Byers & Venturelli, 1994) but have proved to be an intractable problem that continues within this research and policy domain.

Various data sources and methods are used to monitor hate behaviour globally. They are mostly disconnected, and they are shaped by different legislation and use varying terminology, criteria and definitions. Therefore, their operation and performance is often not comparable (even within the same country). A large proportion of information on the extent of hate crimes comes from law enforcement records. However, these registers are affected by numerous limitations, including incident misclassification, differences in reporting patterns between communities, and scarce funding allocated to the training of police officers for data collection. Further inconsistences between registers arise from differences in legislation between jurisdictions, thresholds for proof of bias motivation, data collection criteria, time periods considered in country reports, differences in visibility and acceptance of legislation (Sheppard et al., 2021). Multinational organisations such as the OSCE's Office for Democratic Institutions and Human Rights and the EU Fundamental Rights Agency publish reports of hate crimes reported to police and prosecuted across multiple countries (e.g., Office for Democratic Institutions and Human Rights, 2009). However, national comparisons are difficult to make due to limitations of the types mentioned earlier.

Victimisation surveys are another official source of data on hate crime and its consequences (e.g., physical and emotional costs). Although questions about the bias motivation of a crime are included only in a few countries (e.g., the United States, Canada, England and Wales), when it is present, it is often used to assess the so‐called ‘dark figure’ of hate crime, and to measure under‐reporting. Some organisations like the EU Fundamental Rights Agency run longitudinal cross‐jurisdictional surveys with minority communities that include a focus on hate crime, for example, the LGBT Rights (I and II), EU MIDIS I and II, Roma and Traveller Survey (e.g., European Union Agency for Fundamental Rights, 2020b). Other surveys conducted by government and non‐government organisations (such as universities, research institutes and think tanks) can explore the impact and manifestation of hate crimes, hate incidents and hate speech (Benier et al., 2016; Walters, 2020). Examples are the Leicester Hate Crime Project and the All Wales Hate Crime Project (see Williams & Tregidga, 2014). All surveys can be limited by methodological issues such as being exclusively based on the victim's perception of bias motivation, limitations of the questions asked, and missing categories of victim types including age groups or target identities such as the homeless (Sheppard et al., 2021).

Community organisations and watch‐groups often collect data about hate crime, hate speech and hate incidents (both above and below the criminal thresholds) within their communities. Notable examples are Tell MAMA (measuring anti‐Muslim attacks in the UK), the Anti‐Defamation League (measuring anti‐Semitic attacks in the US) and B'nai Brith (measuring anti‐Semitic incidents in Canada and other countries). Data collected by civil society organisations can be extremely valuable for research purposes and are often used in scholarly research (Green et al., 2001; Vergani et al., 2021). Some argue that watch groups might have a vested interest in inflating the perception of hate against the communities they represent (Kaplan, 1997). While acknowledging this critique, we believe that, in a context where hate crime data quality is often suboptimal because of all the limitations outlined above (Gerstenfeld & Grant, 2004; Saucier et al., 2006), community registers are a key source of data that can be used to compare to other sources (Mason et al., 2017).

Both on‐ and offline media are an important source of primary and secondary hate data. Web searches can be used to retrieve media coverage, law enforcement reports, and non‐profit reports to create a database of bias and hate crimes incidents, as in the case of ProPublica. On‐ and offline media can also be the vehicle of hate speech, and there is a growing scholarship focusing on creating effective automated detection tools to capture and measure it (see Poletto et al., 2021; see also Williams, 2021). Automatic detection of hate speech online, that is, without input from victims or witnesses, is an active field of research. Many of these methods apply Artificial Intelligence to identify hate speech, with ongoing development to improve classification tools, with respect measures such as accuracy, validity reliability, sensitivity and specificity (Williams, 2021).

Other government agencies such as National Human Rights Institutions (NHRIs) often collect reports of hate incidents both above and below the criminal threshold. In countries where NHRIs are tasked with dealing with incidents regulated by civil law (e.g., in Australia), they are an important repository of hate incidents and hate speech reports, although they are often prevented by privacy regulations to share the data that they collect. Government statistical agencies are also important to mention, such as Canada's Statistics Canada and the Canadian Centre for Justice and Community Safety Statistics (CCJCSS).

The proliferation of language used to refer to similar concepts, the lack of definitional clarity and the limitations of measurement approaches pose real challenges to policy, practice and research focusing on tackling hate crime, hate speech and hate incidents. These challenges include problems in:

(1) developing valid and reliable measurement tools to measure hate behaviours, which poses a barrier to:
  a. understanding the real magnitude and trends of hate behaviours;
  b. evaluating the impact of policy and programmes across different jurisdictions and build an understanding of what works and what does not;
  c. building comparative and cumulative knowledge of the causes and solutions of hate behaviours;
(2) developing legal standards, including:
  a. establishing standards for defining the hate element via mechanisms such as demonstrating the presence of a hate element, the hate motivation or the discriminatory selection of the victims;
  b. identifying the groups warranting protection;
  c. treating victims of hate equally across different jurisdictions (both between and within countries) and between groups;
  d. applying legal definition to real cases, and utilising hate crime statutes for criminal prosecution, sentencing, post‐conviction and restorative justice;
  e. developing appropriate legislative structures (such as sentencing and offences);
(3) raising awareness about hate among victims and target groups, among various professional groups including police officers, social workers, government officials, as well as the public;
(4) developing effective policing and policies (including prosecutorial policies and judicial guidance) on hate crime, hate speech and hate incidents, to make it easier navigating the system of reporting hate, receiving support and the criminal justice system for victims of hate;
(5) assessing empirically how and to what extent incidents like hate speech (including online hate speech) and hate incidents might constitute early warnings for hate crime and terrorism incidents.

This review aims to move this field of hate studies (i.e., studies on hate crime, hate speech, hate incidents and surrogate terms identifying specific forms of hate such as racism, antisemitism and homophobia) toward more empirical rigour and theoretical clarity by mapping current and historical approaches to defining and measuring hate crime, hate incidents, hate speech and surrogate terms in North America, Europe, Australia and New Zealand.

### 2.2 Why it is important to do the review

Hate has been a persistent problem across human history (Sternberg, 2003), and it has become an ever more pressing issue in the wake of the COVID‐19 pandemic. In Western contexts, the post pandemic environment has seen a surge in identity based terrorism – such as the May 2022 supermarket mass shooting in Buffalo, New York, that saw an AR‐15 wielding extremist shoot dead ten Black shoppers. Law enforcement agencies documented an unprecedented increase in hate crimes in the United States, the United Kingdom and Canada (Federal Bureau of Investigation, 2022; Home Office, 2022; Statistics Canada, 2022). The UN has recorded a ‘tsunami of hate’ online (United Nations, n.d.) triggered by the Covid‐19 pandemic, characterised by group identities forming around conspiratorial, xenophobic, transphobic and misogynistic content displaying toxic masculinity and racist ideas. Hate groups exploit this environment to promote hateful, extremist narratives, and divisive propaganda, with the aim of recruiting members and polarising the public. Despite the relevance of this policy area, the ability of states to create effective policies to address behaviours motivated by (or demonstrating) hate (i.e., hate crimes, hate incidents and hate speech) is constrained by a general lack of clarity of what constitutes hate, and how to measure it.

Scholars have been calling for a mapping of definitions and measurement tools of the whole spectrum of hate behaviours to map the current developments in policy, practice and research (Schweppe, 2021; Sheppard et al., 2021). This mapping aims to help government and non‐government stakeholders in North America, Europe, Australia and New Zealand inform the next generation of policies, programmes, and research, as well as advocacy for improving legislation. The reasons behind the choice of these jurisdictions is explained in the ‘Population’ section.

### 2.3 How this review might inform or supplement what is already known in this area

Many scholars have discussed the problems associated with the lack of consistent definitions and measurement of hate crime, hate speech and hate incidents both within federal countries like the United States and across different countries in North America and Europe (see Schweppe, 2021; see also Sheppard et al., 2021). They outlined the main issues (as discussed in the first section of this report) and highlighted the tendency for researchers, policy makers and practitioners to work in silos, each developing their own definitions and measurement of hate crime with little (or no) dialogue across sectors (Chakraborti & Garland, 2015a; Perry, 2016). However, no study to date has systematically mapped the field of hate studies. This review provides the first comprehensive mapping of the field by looking at:

(1) the whole spectrum of hate behaviours above and below the criminal threshold (including hate crime, hate incidents and hate speech);
(2) the components of definitions disaggregated by the behaviour captured (whether a behaviour is regulated by criminal law, civil law, or not regulated), the hate motivation (whether it is identified using an ‘animus’, ‘discriminatory selection’ or other approach), the targets of hate (whether any identity or group can be target of hate, or only certain protected characteristics that capture marginaliseised groups, and if so, which ones they are);
(3) different areas of scholarship and practice, including law and statutes, scholarship (differentiating between disciplines), practitioners (differentiating between different types of government and non‐government organisations);
(4) change across different geographical areas;
(5) change over time;
(6) different surrogate terms used to capture the concepts of hate crime, hate speech and hate incidents, including terms used in different jurisdictions (e.g., prejudice motivated crime) and community‐specific terms (e.g., antisemitism);
(7) different types of measurement tools.

The review maps and unpacks strengths and weaknesses of definitions and their operationalisation for measurement. This will benefit the field of hate studies by providing a baseline that can inform the building of cumulative knowledge and comparative research.

We conducted a search of the literature using the following terms to identify existing reviews: hate crime* OR hate speech* OR hate incident*. Searches of the following locations did not identify any existing systematic reviews (completed or ongoing) on the specific topic proposed in this proposal (i.e., definitions and measurements of hate crime, hate incidents and hate speech):

- Campbell Collaboration
- Cochrane Collaboration
- PROSPERO registry
- Google Scholar

## 3 OBJECTIVES

The first review objective is to map definitions of hate crime, hate incidents, hate speech, and surrogate terms. Specific research questions underpinning this objective are:

(a) How are hate crimes, hate speech and hate incidents defined in the academic, legal, policy, and programming literature?
(b) What are the concepts, parameters and criteria that qualify a behaviour as being hate crime, hate incident or hate speech? and
(c) What are the most common concepts, parameters and criteria found across definitions? What are the differences between definitions and the elements they contain?

The second review objective is to map the tools used to measure the prevalence of hate crime, hate incidents, hate speech, and surrogate terms. Specific research questions underpinning this objective are:

(a) How are definitions operationalised to measure hate crimes, hate speech, and hate incidents? and
(b) How valid and reliable are these measures?

In sum, this review identifies a comprehensive list of definitions and measurement tools, as well as a transparent assessment of their operationalisation for measurement, with the aim to support and inform researchers and policy makers.

## 4 METHODOLOGY

### 4.1 Criteria considering studies for this review

#### 4.1.1 Types of studies

This review included documents published after 1990 found in academic literature, grey literature and legislation. In our assessment, 1990 marks the start of an increasing interest in hate crimes among academics and policy stakeholders. In the USA, the Hate Crime Statistics Act passed in 1990 required that the US Department of Justice collect data and publish an annual report summarising the incidence of hate crimes in the nation. In the years since 1990, the USA Congress passed several hate crime laws that provided sentence enhancements when federal crimes were motivated by bias (Farrell & Lockwood, 2023).

Academic literature:

- Empirical articles, with any study design, which propose an original definition or an original measurement tool of hate crime, hate speech, hate incidents or surrogate terms. We included studies with all study designs because definitions and measurement tools can appear in documents presenting all types of empirical research.
- Theoretical articles that focus specifically on defining hate crime, hate speech, hate incidents or surrogate terms.

Grey literature:

- Reports authored by government and non‐government organisations, which propose an original definition or an original measurement tool of hate crime, hate speech, hate incidents or surrogate terms. We expect this literature to include policy and programming areas including political laws, civil acts and codes highlighting the criminality in discriminative actions such as hate speech and hate crime.
- Tech companies' definitions of hate speech and hateful conduct in terms of service, community standard guidelines and transparency reports. Specifically, we included the members of the Global Internet Forum to Counter Terrorism (GIFCT) (17 members as of May 2021).

Legislation:

- Current criminal or civil legislation that is intended to regulate or allow for the collection of data on forms of hate speech, hate incidents and hate crimes. Reported case law were not reviewed because it is a large field that would warrant a separate project with a different approach. Before taking this decision, we reviewed some relevant decisions in common law jurisdictions, and found that they were predominantly with the application, rather than the formulation, of definitions.

Within each document, we looked for definitions (objective 1) and measurement tools (objective 2). We recognised *definitions* as statements that describe what is meant by hate crime, hate incident, hate speech (and surrogate terms). Definitions can be found most likely in the introduction or methods section of a paper, in footnotes, or in a specific section of a grey literature report. The documents might explicitly state that they are ‘defining’ the term (e.g., ‘we define hate crime as…’), or directly describe what is meant by the term (e.g., ‘hate crime is…’). We recognised *measurement tools* as an operationalisation of a definition of any of the concepts of hate crime, hate speech and hate incidents in the form of interview/survey/focus group questions, community reporting tools, coding schemes for visual/audio/textual content, among others. Specifically, we looked for two characteristics in definitions and measurement tools: originality, relevance and transparency. Originality means that if study X used Y's definition or tool, we exclude X and only include Y). For example, a document defining hate crime as ‘generally understood as…’ was excluded because we interpreted this expression as being a synthesis of existing definitions. We excluded definitions that were provided implicitly and within the discussion of existing definitions, and definitions paraphrasing and citing previous work – even without direct quotes – because we regarded them as not original. Relevance means that the study addresses a hate behaviour (see Phenomenon of Interest and Context) in a relevant context (see Population). For example, we excluded definitions that did not directly refer to behaviours. For example, a definition of ‘Islamophobia’ as ‘fear and hatred of Islam’ would be excluded because there is no direct reference to a behaviour or a surrogate term like ‘murder’, ‘crime’, ‘speech’ or other. Transparency (only for measurement tools) means that the study provides a transparent description of the parameters (measures) or indices used for measurement. To be more precise in our methodological approach, we operationalised the meaning of ‘transparency’, ‘relevance’ and ‘originality’ differently for measurement tools and definitions. The next table summarises our understanding of originality, transparency and relevance for definitions and measurement tools (Table 1).

**Table 1:** The meaning of originality, transparency and relevance for definitions and measurement tools.

Objectives	Originality	Transparency	Relevance
(1) Mapping definitions	The definition is presented as an original contribution.	n.a	Defines a relevant behaviour AND The document focuses on a relevant country context OR a theoretical contribution.
(2) Mapping measurement tools	Uses original questions, annotation guidelines, or models to measure or categorise hate behaviours.	Provides a transparent description of all the information collected about the hate incident and the methodology used to collect the data.	Measures the prevalence of a relevant behaviour OR understands the types of manifestations of a relevant behaviourAND focuses on a relevant country context.

#### 4.1.2 Population

We focus on Canada and a sample of countries in different regions that are comparable in terms of democratic institutions, socio‐political context and legislative approach to hate crime, hate speech and hate incidents.

Eligible countries include, in addition to Canada, the United States and the United Kingdom, which represent two key and different approaches to hate crime data collection globally, with the United States generally requiring a crime to present objective indicators of bias to be counted as a hate crime, and the United Kingdom accepting third party perceptions as a valid hate crime indicator at the policing stage (not at the prosecutors or court stage). We include Ireland, Germany, France, Italy and Spain, which represent a heterogeneous sample of European countries in terms of hate crime and hate speech legislation, different socio‐political contexts and approaches to hate crime and hate speech data collection, as identified by recent reports by the European Union Agency for Fundamental Rights (2018a). For example, Germany and Italy have unique hate speech regulations to target hate speech propaganda and hate crime associated with the Nazi and Fascist ideologies. Ireland and Spain include third party perceptions as bias indicator, while France and Germany do not, and Italy does not have a list of bias indicators. Germany and France require by law to record only specific forms of hate crime, Spain and Ireland incorporated hate crime in the general crime recording system, but in Spain recording is not compulsory while in Ireland it is, and in Italy hate crime is not incorporated in the general recording system although hate crime data is collected by law enforcement authorities. All five countries have hate crime and hate speech regulations in criminal law, civil law, media law and press‐self regulation, as well as watch‐group organisations collecting hate incidents reports, which make them relevant case studies. We include Australia, which is a comparable context with Canada in terms of legal, political and democratic structures (e.g., federalism, bicameral parliaments, common law legal system – although Canada has one criminal law, whereas in Australia there are different statutes in each state). We include New Zealand, which after the Christchurch attack significantly increased its efforts in policy, practice and research on hate behaviours, as demonstrated by the Christchurch call to eliminate terrorist and violent extremist content online, and by the Royal Commission of Inquiry on the Christchurch attack, and its numerous recommendations relevant to regulating hate crime, hate speech and hate incidents.

The review considers academic articles in English, French, German, Italian and Spanish languages, and review relevant contemporary legal statutes, policy and programming in North America (Canada and United States), Europe (Germany, France, United Kingdom, Ireland, Italy and Spain), Australia and New Zealand.

#### 4.1.3 Phenomena of interest

This review focuses specifically on capturing both general definitions of hate crime, hate speech and hate incidents; and definitions of surrogate terms capturing hate directed towards identities based on the following perceived characteristics that appear in Canada's legislation:

(1) Racial and ethnic identity (e.g., racism, xenophobia, sinophobia);
(2) Religious identity (e.g., antisemitism, anti‐Muslim);
(3) Sexual orientation (e.g., homophobia, lesbophobia, biphobia);
(4) Gender identity (e.g., sexism, misogyny, transphobia);
(5) Disability (e.g., ableism, disablist violence);
(6) Occupation identity (e.g., anti‐abortion violence).

Our review pays particular attention to how protected characteristics are defined and captured, and how other characteristics absent from lists are captured by existing definitions and measurement tools. In this review, we focus exclusively on definitions of malicious behaviours motivated by (or demonstrating) prejudice, or by a worldview or ideology that dehumanises the target group and justifies aggression and violence, and how these definitions are operationalised to measure these behaviours. Behaviours that are included in this review can be criminal behaviours (e.g., murders, violence against people and properties, and forms of hate speech regulated by the criminal code) and non‐criminal behaviours (e.g., the so‐called ‘protected hate speech’ in the United States or other jurisdictions). Both on‐ and offline behaviours are included in our scope. Discrimination (e.g., discrimination in workplace, sale and supply of services), grievance fuelled‐violence, fixation, sex crimes and harassment are excluded because they are different fields of literature, which we believe would need a separate independent review, especially in relation to the legal literature.

#### 4.1.4 Context

Much of the literature focusing on ‘hate crime’, ‘hate speech’, ‘hate incidents’ or any surrogate terms (such as homophobia, Islamophobia or antisemitism) focuses on negative attitudes to out‐groups instead of behaviours (see e.g., Uenal et al., 2021; see also Huynh et al., 2020). This area of literature focusing on attitudes, although using concepts that might be relevant for this review (such as homophobia, Islamophobia and antisemitism), is excluded because our main focus are definitions and measurements of behaviours, not cognitive activities. As we only focus on measurement tools capturing behaviours, we do not review any psychometric scale instrument, or any tool aiming to capture attitudes related to hate or surrogate terms (e.g., racism, homophobia, antisemitism, etc.). Similarly, we exclude definitions that describe these terms as attitudes and emotions (e.g., defining Islamophobia as ‘fear of Islam’).

### 4.2 Search methods for identification of studies

As described in our Protocol (Vergani et al., 2022), our systematic search involved a variety of strategies tailored to retrieving academic and grey literature, as well as legislation.

#### 4.2.1 Electronic searches

We used EndNote to manage all references we retrieved from these searches. Our search strategies differed depending on the search capacities of the databases and citation indexes in question. We broadly distinguished between databases and citation indexes with complex search capacities and those with limited search capacities. The former included those that allowed us to combine all our search terms with Boolean and proximity operators into a single search string, whereas the latter included those that did not. Where available, we used search limits and filters to filter out material published before 1990 and on or after 1st January 2022 as well as document types ineligible for inclusion (e.g., news items, audio‐visual material, letters to the editor), and to narrow the search results to disciplines eligible for inclusion.

For databases and citation indexes with complex search capacities (e.g., Scopus), our search strings systematically combined attribute‐, behaviour‐ and country‐specific search terms as per our protocol (Vergani et al., 2022). Table 2 reports the full list of search terms that were used for electronic searches. Within each category, we connected all search terms with a Boolean OR operator. We combined all attribute‐ and behaviour‐specific keywords with a proximity operator, and then combined these with the country‐specific keywords with a Boolean AND operator. We applied wildcards to the search terms as appropriate. We used these keywords to search in title, abstract and subject fields, depending on the database and citation index. The full search strategy is provided in the Supporting Information: Appendix.

**Table 2:** Search terms for electronic searches.

	Attribute	Behaviour	Geographical context
Generic	hate	crim*	‘United States’
	prejudice*	speech	‘US’
	bias*	incident*	‘USA’
Racial and ethnic	racis*	conduct	Australia*
	xenophobi*	act	‘New Zealand*’
	sinophobi*	abus*	Aotearoa
	anti‐foreigner	vilif*	France
	anti‐migrant*	language*	French
	anti‐immigrant*	violen*	German*
	anti‐refugee*	rape*	Irish
	‘anti‐asylum seeker’	murder*	Ireland
	anti‐Roma	harass*	Ital*
	anti‐traveller	terroris*	Spain
	anti‐Gypsy	narrative*	Spanish
	‘anti‐First Nations’	discourse*	‘UK’
	anti‐Indigenous	propaganda	‘United Kingdom’
	anti‐Maori	‘targeted violence’	Brit*
	anti‐Aboriginal	incite*	Engl*
Religious	islamophobi*	extremis*	‘Northern Ireland’
	antisemiti*	hostil*	‘Northern Irish’
	anti‐Semiti*	micro‐aggression	Scot*
	anti‐Jew*	microaggression	Wales
	anti‐Amish	group*	Welsh
	anti‐Sikh		Canad*
	anti‐Buddhis*		
	anti‐Muslim*		
	anti‐Islam*		
	anti‐Christian*		
LGBTIQ+ and non‐binary	homophobi*		
	transphobi*		
	lesbophobi*		
	biphobi*		
	anti‐gay		
	anti‐lesbian*		
	anti‐bisex*		
	anti‐transgender		
	anti‐LGBT*		
Disability	ableis*		
	disableis*		
Gender	sexis*		
	misogyn*		
	misandr*		
	gender‐based		
	‘gendered’		
	incel		
	invcel		
	‘involuntary celibate’		
	anti‐feminis*		
Occupation	anti‐abortion		
	anti‐doctor		
	‘anti‐sex worker’		
	anti‐politician		

*Note*: Please note that the terms are designed to capture how hate behaviours are defined in the existing literature, they do not reflect terminology choices by the research team. Searches will be performed by combining all ‘attributes’ with all ‘behaviors’ and all ‘geographical contexts’.

We adapted this search string to the specificities of the platforms hosting the following databases and citation indexes:

- EBSCOHost
  ○ Communications and Mass Media Complete
  ○ Criminal Justice Abstracts with full text
  ○ SocIndex with full text
- ProQuest
  ○ Contintental Europe Database
  ○ Dissertations and Theses Global
  ○ Education Resources Information Centre (ERIC)
  ○ Sociological Collection
  ○ Technology Collection
- Web of Science
  ○ Web of Science Core Collection
  ○ SciELO citation index
- Ovid
  ○ PsycInfo
- Scopus

For databases and citation indexes with limited search capacities (e.g., Google Scholar), we used a list of eight keywords:

(1) ‘hate crime’
(2) ‘hate speech’
(3) ‘hate incident’
(4) ‘hate conduct’
(5) ‘hate propaganda’
(6) ‘hate group’
(7) ‘prejudice‐motivated crime’
(8) ‘bias crime’

We chose these keywords because they are the most relevant to our project. We expected them to have equivalents in the other languages considered in our review in the form of direct translations. For searches of Google Scholar carried out in Italian, Spanish, French and German, we translated those keywords into the respective languages. Where possible, we combined the eight search terms in a single search combining them with the Boolean operator OR. Where this was not possible, we carried out individual searches for each term separately. We searched the following databases and citation indexes:

- Science Direct
- Google Scholar
- National Criminal Justice Reference Centre Abstracts Database
- EUR‐Lex
- OSCE Office for Democratic Institutions and Human Rights Document Library
- United Nations Digital Library
- United Nations Office of the High Commissioner for Human Rights Digital Library

#### 4.2.2 Searching other sources

We complemented our electronic searches with other search strategies to minimise the risk of bias. Specifically, these complementary strategies included:

(1) We identified stakeholders in relevant fields with the aim of detecting potentially relevant organisations, websites, and documents, including: unpublished or ongoing projects, key government and non‐government organisations producing relevant grey literature, and legislation/statutes in each country context.
(2) We searched websites of relevant organisations for potentially relevant documents that we identified based on our previous research and existing professional networks, through hand‐searching of included documents, and based on feedback from our External Advisory Board.
(3) In order to identify all legal documents that might contain a relevant definition, we devised an additional and complementary search strategy in the following databases and websites: WorldLII, the Justia database, the UN digital library, the UN Treaty Body database, the EUR‐Lex database, the Council of Europe website, the OSCE hate crime database, the Anti‐Defamation League's website. Search terms for identifying relevant legislation and international instruments included: hate, hatred, bias, prejudice, malice, ill‐will, hostility, contempt, discriminat*, incite*, stir*, motivat*, aggravat*, race, raci*, religio*, ethnic*, sex*, gender, church, temple, synagogue, mosque, masjid, worship, cross, swastika.
(4) We contacted a legal scholar or practitioner with expertise in each country's context to assist with making sure that we had an exhaustive list of the most up‐to‐date legislation.

#### 4.2.3 Criteria for determining independent findings

The same definition or the same measurement tool can be contained in multiple documents. During the first round of data analysis, we performed manual checks to identify identical definitions and identical measurement tools. We clustered multiple identical copies, and retained only the most complete version (e.g., the version containing more information about a measurement tool, or more discussion about a definition), or – if identical – the first published version.

### 4.3 Data collection and analysis

#### 4.3.1 Description of methods used in primary research

What follows are exemplars of the types of papers that were included in the review and their uses. Mason (1993) examines the prevalence of anti‐homosexual violence in Australia, its characteristics, and its impact on the targets. The document also examines the cultural climate and social norms that encourage anti‐homosexual violence. Mason draws on the following definition of hate crime:

Hate crime refers to crime, most commonly violence, motivated by prejudice, bias or hatred towards a particular group of which the victim is presumed to be a member (Mason, 1993, p. 1)

This definition of ‘hate crime’ outlines three key criteria: first, hate crime is a ‘classic crime’, that is, the behaviour in question is defined as criminal under the law; second, hate crime is identifiable by the perpetrator's motivation to engage in criminal behaviour; third, hate crime is identifiable based on the discriminatory nature of the choice of the victim. This work meets our inclusion criteria (2) and (3) in relation to objective 1, as it focuses specifically on hate crimes and offers a specific definition of hate crime.

In relation to measurement tools, the content that we analysed is extremely diverse. We identified eight different types of measurement tools with very distinctive characteristics:

1. Law enforcement data
2. Incidents reporting tools
3. Case records and open source databases
4. Quantitative survey questionnaires
5. Manual quantitative text analysis tools
6. Automated or semi‐automated text analysis tools
7. Qualitative interviews schedules
8. Qualitative text analysis tools

In this review, we did not include documents using law enforcement data because – even though this data is used by academics and is contained in official reports published by government organisations – the full methods underlying the functioning of law enforcement data are often absent from the studies reporting the data. A complete and meaningful assessment of cross‐country law enforcement data measuring hate crimes (including state level data collection in federal countries) would require a design and data collection methodologies that are outside the scope of this systematic review, such as qualitative interviews, content analysis of guidelines and training materials, and potentially ethnographic methods and/or quantitative surveys. However, as part of this review, we provide a general overview of the main characteristics of law enforcement hate crime data in the countries under investigation, based on recent reports (European Union Agency for Fundamental Rights, 2018a), private correspondence with law enforcement officials and recent official resources on hate crime data collection protocols such as the OSCE website on hate crime (hatecrime.osce.org).

Most incidents reporting tools data are collected by government and nongovernment organisations. Although the data is sometimes used in academic research, in this review we only included documents describing relevant data and methods published by government agencies in grey literature. All other measurement tools are mostly found in academic research. We include documents describing relevant data and methods. The next table provides a list of the eight types of measurement tools that we mapped in this project, alongside a description and an example (Table 3).

**Table 3:** Types of measurement tools, descriptions and examples.

Types of measurement tools	Descriptions	Examples
Law enforcement data	Data collected by police or other law enforcement agencies who either respond to a crime report by a victim or a witness, or actively investigate a crime.	Hate crimes reported as part of the National Incident‐Based Reporting System (NIBRS) in the USA
Incidents reporting tools	A web‐based form, sometimes in conjunction with an email and/or a phone number, that victims or witnesses can use to report a hateful act.	True vision UK (https://www.report-it.org.uk/)
Case records and open source databases	Repositories of information about hate behaviours compiled by researchers using a variety of open and classified sources.	BIAS data set compiled by START (https://www.start.umd.edu/research-projects/pathway-approach-study-bias-crime-offenders)
Quantitative survey questionnaires	Sets of questions used in survey research to measure the prevalence of a hate behaviour.	Hate crime questions collected as part of the Crime Survey for England and Wales (CSEW)
Manual quantitative text analysis tools	Guidelines to analyse text with the aim to measure the frequency of a hate behaviour.	Hameleers et al.'s (2021) study, which analyses false statements to identify the presence or absence of hate speech
Automated or semi‐automated text analysis tools	Instruments that automate all or part of the identification of hate speech found in text or other multimedia content using a model.	Hatemeter (http://hatemeter.eu), a an ICT tool that automatically monitors and analyses Internet and social media data on anti‐Muslim hatred online
Qualitative interviews schedules	Lists of interview questions and/or protocols aiming to understand the different manifestations of a hate behaviour in the eye of a victim or a witness.	Sandhu's (2019) study, which provides a list of questions for semi structured interviews to investigate Sikh Americans’ experiences of racial and religious discrimination
Qualitative text analysis tools	Guidelines to analyse text with the aim to understand the different manifestations of a hate behaviour.	Brindle's (2016) study, which uses discourse and linguistic analysis to analyse text produced by a hate group and identify different manifestations of Islamophobia

### 4.4 Selection of studies

We used EndNote to manage all documents retrieved throughout the search process. All academic documents were imported into Endnote. We then imported the results of our academic and grey literature searches from EndNote into EPPI Reviewer Web. We removed duplicates in EPPI Reviewer Web and categorised references by language (English, German, French, Spanish, Italian) before beginning title and abstract screening. Because we included documents in English, French, German, Italian and Spanish, we progressed through title and abstract screening in stages. First, we completed title and abstract screening for references in English before in turn completing title and abstract screening for references in French, German, Italian and Spanish.

#### 4.4.1 Title and abstract screening

Inclusion and exclusion criteria for objective 1 and 2 were identical for title/abstract screening and are provided in the table below (Table 4).

**Table 4:** Inclusion criteria for title/abstract screening.

Inclusion criteria
Document was published in or after 1990.
Document explicitly focuses on hate crime, hate incidents and/or hate speech or a surrogate for these terms.
Document focuses on hate crime, hate incidents and/or hate speech or a surrogate for these terms in a geographical context within the scope of this review (Canada, USA, UK, Ireland, France, Germany, Spain, Italy, Australia and New Zealand).
Document focuses on behaviours that can be classified as hate crime, hate incidents and/or hate speech (examples are: murder, physical aggressions, property damage, vandalism, graffiti, offensive gestures, offensive or abusive social media posts).
Document can be classified as academic literature, grey literature, legal literature or federal and state statutes.

Following a training and induction session, we conducted two rounds of title and abstract screening of English‐language references to gauge intercoder reliability. In each round, screeners screened the titles and abstracts of the same 49 references. We retrieved the references as a random sample of all references remaining in EPPI Reviewer Web following the removal of duplicates. Intercoder reliability for the first round was 0.77 and 0.85 for the second. Subsequently, we reconciled disagreements by majority decision in EPPI Reviewer Web. The 98 references used for intercoder reliability calculations served as the initial input into EPPI Reviewer Web's machine learning functionality ‘Priority Screening’ that we used to support title/abstract screening (EPPI‐Centre, n.d.). Previous research found that EPPI Reviewer Web's ‘Priority Screening’ functionality had the potential to reduce the screening burden by up to 60% (Tsou et al., 2020). It orders references based on their likelihood to be included based on previous screening decisions, with those references deemed more likely to be included presented to screeners ahead of those deemed less likely to be included. In short, the more references had been screened, remaining references were less likely to be included.

**Figure 1:**
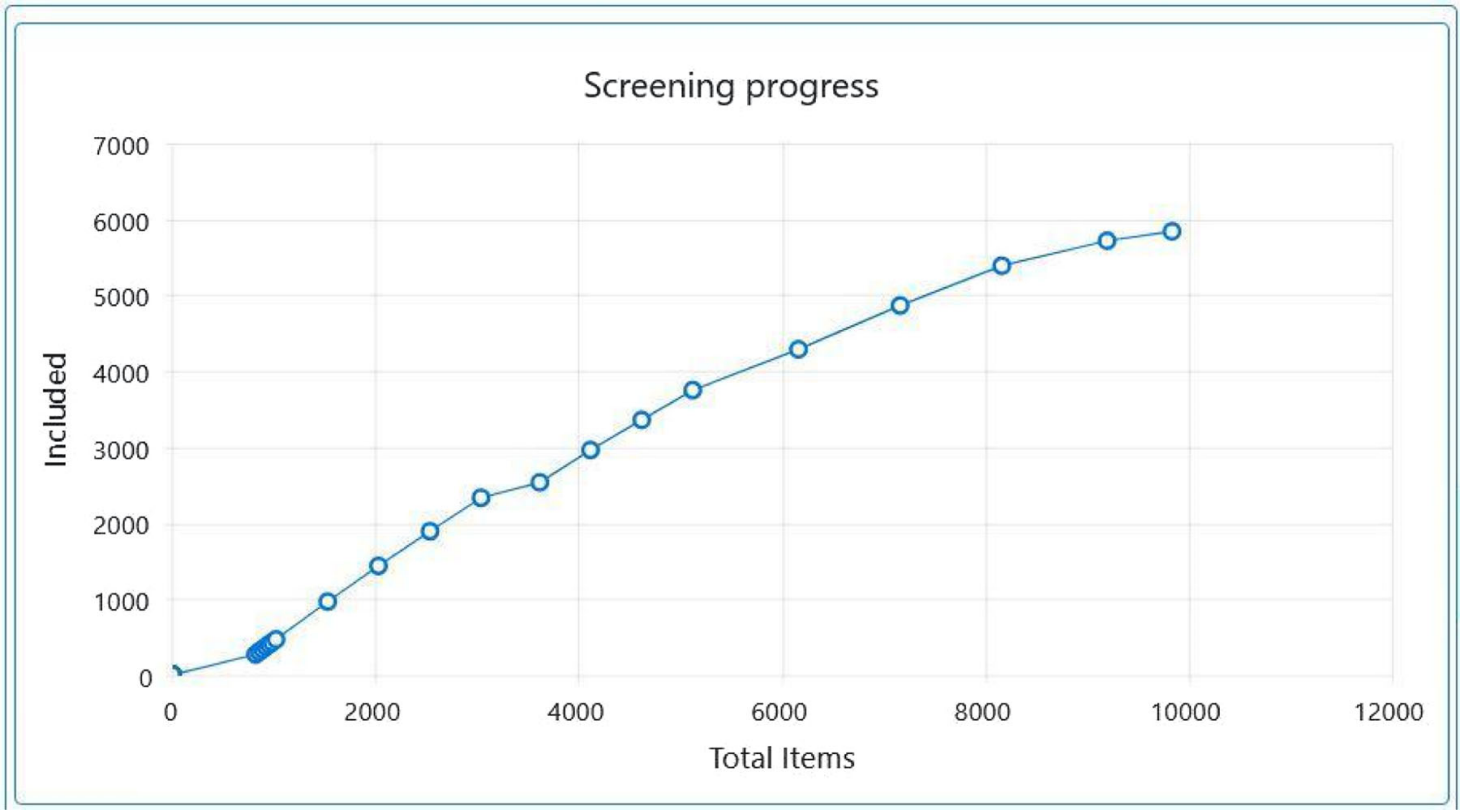
Line plot of the ratio of included items to total items during title and abstract screening.

Thus, throughout title and abstract screening of English‐language references, we monitored screening progress as the ratio of included items to total items screened to determine a ‘stopping point’ at which we would stop screening titles and abstracts of remaining references. We identified this ‘stopping point’ as the point at which the curve of a line plot of the ratio of included items to total items would flatten, indicating that that ratio was decreasing. We determined, following team consultations, that we had reached a suitable ‘stopping point’ after having screened titles and abstracts for 9839 English‐language references (see Figure 1).

Subsequently, we used another EPPI Reviewer Web's machine learning functionality (EPPI‐Centre, n.d.) to build a model that could classify remaining English‐language references as either ‘relevant’ or ‘irrelevant’ based on previous title‐and‐abstract‐screening decisions. This classifier calculates and applies a score ranging from 0 to 99, with a higher score indicating that the reference was more likely to be deemed ‘relevant’. The classifier groups references into deciles based on their assigned score. In our case, there were no references in the 70–79, 80–89 and 90–99 deciles. We manually screened the titles and abstracts of all references in the 0–9, 40–49, 50–59 and 60–69 deciles. We manually screened the titles and abstracts of 100 references each in the 10–19, 20–29 and 30–39 deciles. We included 1 out of 100 manually screened references in the 30–39 decile and thus proceeded to code all references in this decile manually. Because we did not include any of the manually screened references in the 10‐19 and 20‐29 deciles, we decided to exclude all references in these deciles without title and abstract screening. In total, we excluded 7943 references in this way.

Having completed title and abstract screening for English‐language references, we proceeded with title and abstract screening for references in French, German, Italian and Spanish. References in each of these languages were screened manually applying the same exclusion criteria outlined above by a native speaker.

#### 4.4.2 Full text screening

After completing title and abstract screening, we proceeded with full text screening. In addition to the 5 exclusion criteria for title and abstract screening above, we used two more exclusion criteria to assess eligibility for inclusion in the review based on the full text:

1. Exclude if full text of reference does not contain any definition nor measurement tool of hate crime, hate speech or hate incident, or any surrogate term.
2. Exclude if full text of reference does not contain an *original* definition nor an *original* measurement tool of hate crime, hate speech or hate incident, or any surrogate term.

In cases in which screeners could not unambiguously exclude or include a reference based on these criteria, we asked them to discuss it with one of the chief investigators on the project and come to a joint decision. Following a training and induction session, we conducted 3 rounds of full‐text screening to ascertain intercoder reliability. Each round consisted of 20 references. Intercoder reliability for the first round was 0.78, 0.82 for the second and 0.86 for the third.

Given the large volume of documents that needed to be screened for full text, we devised (in conjunction with the Campbell Collaboration Crime and Justice Coordinating Group and the Campbell Collaboration Methods Groups) an ad hoc methodology to avoid the second screening of all full texts, which would have been impracticable within the time and budget constraints of this project. Following training and intercorder reliability sessions, each full text was initially screened by one trained screener. Upon completion of this first round of full‐text screening, we conducted second screening of:

- all the included references. If the second screener wanted to exclude the document (i.e., the first decision was to include, but the second decision was to exclude), we asked them to directly resolve their disagreement with the screener who made the initial inclusion decision.
- a random sample of 10% of excluded full texts were re‐screened by a second screener. For measurement tools in the discipline of computer science, the computer scientist in our team performed the second screening of all the included documents, as well as the randomly selected 10% of excluded documents. Similarly, for legislation documents, the legal scholar in our team assessed the eligibility and inclusion criteria of all legislation, aided by a small team of 2 research assistants and 2 chief investigators. Less than 3% (17/596) of the excluded references re‐screened were included following the second round of screening. Based on this result, we did not deem it necessary to re‐screen all excluded items.

### 4.5 Data extraction and management

We used Qualtrics forms for data extraction. We conducted training and induction sessions for all coders. We instructed reviewers to resolve any questions about particular references or definitions in discussion with other chief investigators. A second coder reviewed all coding decisions. The full list of variables coded is available in the Supporting Information: Appendix.

#### 4.5.1 Review objective 1: Definitions

To achieve objective 1, for each document we extracted information about the components of the definition of hate crime, hate speech, hate incidents and surrogate terms. *Components* include:

(1) how the motivation is named (e.g., racist, Anti‐Semitic, hate, etc.);
(2) how the nature of the behaviour is named (e.g., crime, incident, speech, act, etc.);
(3) how the target is described (e.g., a person, a property, a person and property, etc.);
(4) whether animus or other approaches are used to identify the hateful motivation; and
(5) the protected characteristics (if any).

Additionally, we coded variables not contained in the document, such as the uptake of the definition (based on Google Scholar citations of the document). For definitions in legal documents, we created two different data extraction forms. The legal expert in our team completed data extraction for legislation categories we deemed more technical in nature (e.g., type of legislation). For less technical data (e.g., protected characteristics), we provided training and induction session to two reviewers. This involved having reviewers independently code the same 5 pieces of legislation to ensure reliability and providing detailed feedback. Chief investigators and the legal expert reviewed the data upon completion of data extraction to ensure its accuracy.

#### 4.5.2 Review objective 2: Measurement tools

To achieve objective 2, we extracted information about: the type of measurement tool, defined as a vehicle or an aid to collect information and data (e.g., an online module to collect data about hate incidents, or an automated text analysis algorithm, or a survey), and the metrics used to measure hate, defined as parameters (measures) or indices used for measurement, comparison or tracking performance (e.g., bias indicators). We coded categorical variables looking at information about the target identities that the measurement tool encompasses, the variables collected about the incident, the victims, and the offender. To be more precise in the data extraction process, we created different tailored data extraction forms for different types of measurement tools. For example, computer science measurement tools focused on extracting unique features such as coefficients (e.g., F1 scores) and processes (e.g., pre‐processing of the data). Qualitative measurement tools focused on retrieving specific information about the interview or text analysis process. The data extraction for the measurement tools was performed by team members with relevant expertise in the methodology covered. For example, the data scientist in our team extracted the data for computer science measurement tools, a researcher with quantitative expertise for survey data and databases, and a researcher with qualitative expertise for qualitative measurement tools.

### 4.6 Assessing the methodological limitations of included studies

We propose the following criteria to assess the operationalisability of definitions (for objective 1) and the theoretical robustness and transparency of measurement tools (for objective 2).

#### 4.6.1 Review objective 1: Definitions

In the context of this review, we assessed the operationalisability of definitions, that is, how capable a definition is to operationalised. We appraised operationalisability by looking at three key indicators:

1. ambiguity of language;
2. conceptual sophistication;
3. uptake.

These three indicators were operationalised using different sources of data as explained in the following table (Table 5).

**Table 5:** Indicators, rationale, variables and measure used to assess the operationalizability of the included definitions.

Indicator	Rationale	Operationalisation	Measure (normalised)
Ambiguity	Does the definition contain unambiguous concepts (Ritzer, 1991)?	LIWC (Linguistic Inquiry and Word Count) category of emotional language	Between 0 and 1 (where 1 indicates less emotional language and 0 more emotional language)
Sophistication	Is there a justification or rationale for inclusion of a certain element or component of the definition?	Definitions are coded to identify discussion of conceptual issues	Between 0 and 1 (where 1 indicates more sophistication, and 0 less sophistication)
Uptake	Is the document containing the definition cited by other documents?	Google Scholar citations for each document containing the original definitions	Between 0 and 1, where 1 indicates the highest number of citations in the sample, and 0 indicates zero citations or missing information

To assess ambiguity (which we operationalised as the use of ‘emotional’ language) in the included definitions, we used the 2022 version of the software Linguistic Inquiry and Word Count (LIWC‐22) (Boyd et al., 2022). We used LIWC‐22 to calculate the percentage of words expressing emotions within each definition. The ‘emotion’ dictionary has been validated and tested in multiple research projects over 30 years, and it contains 1030 English words (e.g., ‘bad’, ‘hurt’, ‘worry’, ‘fear’, ‘afraid’, etc.). We used Google Translate to translate definitions in Italian, German, Spanish and French into English. The percentage of ‘emotion’ words ranged between 0% and 16.67% in the sample. We then reverse coded and normalised the scores between 0 and 1 (when 0 indicates less emotional language, and 1 more emotional language).

To assess sophistication in the included definitions, we coded whether the definition and the surrounding text (including the paragraphs before and after the definition) discussed at least one of the following conceptual issues, which we regard as key conceptual elements of the definition of any hate behaviour: interchangeability of the victim, degrees of motivation, bias indicators, whether only minorities are legitimate or main targets. If the definition discussed at least one of these issues, we assigned the score 1. If it didn't, we assigned the score 0. Additionally, we looked at how precise each definition was in defining the following elements:

- The ‘hate element’ (e.g., whether the definition used an explicit approach to appraise whether a behaviour is hateful). We assigned the score 1 if the definition contained a discussion of the hate element, and 0 if it didn't.
- The protected characteristic (e.g., race, ethnicity, religion). We assigned the score 1 if the definition listed one or more protected characteristics, 0 if it didn't.
- The targets of the hate behaviour (e.g., groups, individuals, property). We assigned the score 1 if the definition contained at least two targets (e.g., ‘individuals and groups’ or ‘individuals and properties’), and score 0 if it contained only one target (e.g., only ‘groups’).
- The behaviours included in the scope of the definition (e.g., murder, harassment, cyber‐abuse). We assigned the score 1 if the definition listed at least one relevant behaviour.

We then created a scaled item ranging between 0 and 5, and we normalised the scores between 0 and 1 (when 0 indicates less sophistication, and 1 more sophistication).

To assess uptake, we counted the Google Scholar citations for each document. About a quarter (*N* = 104) of the documents containing our original definitions were not in Google Scholar, so we could not assess their uptake. The remaining documents were cited between 0 and 1372 times. We normalised the scores between 0 and 1 (when 0 indicates less uptake, and 1 more uptake).

Finally, we created a composite quality score by averaging the normalised scores across the three indicators. For the definitions that were not in Google Scholar and we could not assess ‘uptake’, we only averaged two indicators ‘ambiguity’ and ‘sophistication’. We then used this score to assign four categories to each definition: definitions with an average quality score between 0 and 0.25 were rated as ‘low operationalisability’, between 0.26 and 0.50 ‘medium‐low operationalisability’, between 0.51 and 0.75 ‘medium‐high operationalisability’, and between 0.76 and 1 ‘high operationalisability’.

Importantly, the three indicators (ambiguity of language, conceptual sophistication, uptake) are relevant to appraise only the definitions found in the academic and grey literature. Definitions found in legislation should be appraised using a different approach, which includes the analysis of the cases in which the legislation has been applied, as well as interviews with stakeholders (e.g., prosecutors, judges) to understand the practical use of a definition and – more generally – a legislation. This methodological approach is not practicable within the scope of this project.

#### 4.6.2 Review objective 2: Measurement tools

To appraise the theoretical robustness and transparency of the included measurement tools, we used six indicators of quality. The first three are adapted from the OMERACT filter: ‘truth’, ‘discrimination’, and ‘feasibility’. Truth refers to whether the measure's scores can be shown to be truthful, measuring what was intended. In our case, we looked at the completeness of the information collected (i.e., whether the document discusses at least one of the key issues related to the truthfulness of the data, such as bias, reliability and validity, data and research quality). Discrimination asks whether the measure discriminates between situations of interest, such as between hate and non‐hate behaviours. We looked at whether the documents included a discussion of how the hate element was identified and operationalised, whether the questions and guidelines about how to identify the hate element were reported, and whether the tool captured stability in context of change. Finally, as information about costs and burden linked to each instrument was unavailable, we operationalised feasibility as whether the document included discussions about either the ethics or replicability of the instrument.

To tailor and complete our appraisal of the theoretical robustness and transparency of the measurement tools, we added three more indicators: ‘uptake’, ‘theoretical robustness’ and ‘transparency’. We operationalised uptake looking at whether instruments are at different stages of development, which include: (1) concept development; (2) pilot; and (3) wide‐spread application. This indicators of uptake was used to assess measurement tools in previous Campbell cap gap analyses (Sparling et al., 2019). To measure theoretical robustness, we looked at whether the measurement tool was underpinned by a definition of the relevant hate behaviour. To measure transparency, we looked at whether the measurement tool provided a link to raw data collected, including coded or fully anonymised data. Together, these six indicators describe a set of standards which, when met, answer one question: Is there enough evidence to support the use of this instrument to measure the prevalence and the manifestations of a hate behaviour? The next table summarises the indicators and variables that we used to assess the theoretical robustness and transparency of all measurement tools (Table 6).

**Table 6:** Indicators and variables used to assess the theoretical robustness and transparency of all measurement tools.

Indicator	Variable
Truth (from OMERACT)	The document containing the measurement tool discusses at least one of the following issues: under‐reporting, bias in data and research process, reliability, validity
Discrimination (from OMERACT)	The document containing the measurement tool discusses at least one of the following issues: how the hate element is measured, the questions or annotation guidelines used to detect the hate element, stability in situation of no change
Feasibility (from OMERACT)	The document containing the measurement tool discusses at least one of the following issues: replicability of the procedures, ethical issues related to the measurement process
Uptake	The measurement tool is at ‘wide‐spread’ application stage (i.e., used more than once across different geographical locations or time periods)
Theoretical robustness	The measurement tool explicitly refers to a definition of the relevant hate behaviour
Transparency	The document containing the measurement tool provides a link to the raw data (including anonymised or coded data)

### 4.7 Description of data mapped

For conceptual clarity, we distinguish between definitions and measurement tools of hate crime, hate speech and hate incidents by adopting a clear conceptual framework. In the context of this review, we see ‘hate crime’ as a crime recognised by the law which is committed with an additional hate element, and ‘hate speech’ as an expression act that – without the hate element – would not constitute a criminal behaviour and that might (or might not) be criminalised in a certain jurisdiction (Schweppe, 2021). In sum, a ‘hate crime’ – as opposed to ‘hate speech’ – presumes a predicate offence that would be criminal even without the hate element. We coded as ‘hate incidents’ the definitions with a specific focus on non‐criminal behaviours that contain a hate element but do not meet the threshold for a crime and are not framed as ‘hate speech’ (e.g., micro‐aggressions). Finally, we had a residual category for the definitions that captured behaviours potentially across multiple categories (e.g., both ‘hate crime’ and ‘hate incidents’). Table 7 provides examples of definitions in each category.

**Table 7:** Examples of hate crime, hate speech, hate incidents definitions.

Category	Example
Hate crime	Hate crime refers to crime, most commonly violence, motivated by prejudice, bias or hatred towards a particular group of which the victim is presumed to be a member (Mason, 1993).
Hate speech	Hate speech refers to acts of speech with an expressive‐communicative content of hatred or prejudice of the author towards a certain person due to a personal condition, or that generate a discriminatory effect in a group characterised by a personal condition (translated from Spanish language using Google Translate; de Botton et al., n.d.).
Hate incidents	Any non‐crime incident which is perceived by any person to, in whole or in part, be motivated by hostility or prejudice, based on actual or perceived age, disability, race, colour, nationality, ethnicity, religion, sexual orientation or gender (An Garda Síochána, 2019).
Multiple categories	For the purposes of this study, hate crimes and discrimination are defined as: physical and attempted physical violence on people or property; threats and intimidation; insulting behaviour; interruption of religious services, lectures, and celebrations; or vandalism, theft, and loss of employment and other opportunities based upon conversion to Islam (Singleton, 2017).

#### 4.7.1 Review objective 1: Definitions

Given the qualitative textual nature of the information extracted we attributed mostly categorical codes to the text extracted. The unit of analysis are the definitions. For each definition, we code components (e.g., attribute, behaviour, protected characteristics, etc.) and other additional qualitative categories (e.g., how the hate element is identified).

To understand the components of for each definition, we coded: the hate qualifier, the behaviour, the target and the protected characteristics. The next table provides an example of how we categorised the components of a definition (Table 8).

**Table 8:** Example of coding of a definition's components.

Full text of the definition		Hate speech is intentional or unintentional public discriminatory and/or defamatory statements; intentional incitement to hatred and/or violence and/or segregation based on a person's or a group's real or perceived race, ethnicity, language, nationality, skin colour, religious beliefs or lack thereof, gender, gender identity, sex, sexual orientation, political beliefs, social status, property, birth, age, mental health, disability, disease. (International Network Against Cyber Hate, n.d.)
Components	Hate qualifier	Hate
	Behaviour	Speech
	Targets	Person, group
	Protected characteristics	Race, ethnicity, language, nationality, skin colour, religious beliefs or lack thereof, gender, gender identity, sex, sexual orientation, political beliefs, social status, property, birth, age, mental health, disability, disease

The identification of the ‘hate element’, that is, the criteria used to distinguish between a ‘hate’ and ‘non‐hate’ behaviour, is a fundamental aspect of all definitions of hate crimes, hate speech and hate incidents. We used two different models to categorise and map the identification of the ‘hate element’ in our corpus of original definitions, because for hate crimes the ‘hate element’ is an additional component of a crime (e.g., a murder, a property damage, an assault), while for hate speech and hate incidents the ‘hate element’ is the act itself (e.g., a racially motivated insult or a hateful social media post). This means that there are different processes involved in the identification of the ‘hate element’: for example, in relation to hate speech, a key approach is about demonstrating that the behaviour is harmful. This is meaningless in relation to hate crime, because hate crime criminal offences (e.g., assault, theft, murder) don't need a demonstration of harm.

To categorise hate crimes, we distinguished between the ‘discriminatory selection’, ‘animus’, and ‘demonstration’ models (Lawrence, 1999; Walters, 2020).

- The ‘discriminatory selection’ model, which defines the hate element in terms of the offender's discriminatory selection of the victim. Under this model, ‘it is irrelevant why an offender selected his victim on the basis on race. It is sufficient that the offender did so’ (Lawrence, 1994, p. 324).
- The ‘animus’ model, which defines the hate element on the basis of the perpetrator's animus or intent to the victim's identity and the centrality of this animus in the perpetrator's motivation for committing the crime (Lawrence, 1994).
- The ‘demonstration’ model, which is adopted in some countries (e.g., England, Wales, Malta) where there is no requirement to evidence a hate motivation. A *demonstration* of hatred is sufficient to move a crime across the threshold to become a hate crime (Walters, 2020).

To categorise hate speech, hate incidents and acts across multiple boundaries, we distinguished between ‘teleological’, ‘consequentialist’ and ‘formal’ models (Hietanen & Eddebo, 2022).

- ‘Teleological’ models define hate behaviours by their intention and tendency of the act, which tend towards specific negative effects. This category overlaps with the ‘animus’ model.
- ‘Consequentialist’ models define hate behaviours by their effects or perceived effects, regardless of their perceived intent.
- ‘Formal’ models define hate behaviours by character of the act and ideas involved that are described as unethical, immoral, derogatory or using other attributes. We consider Hietanen and Eddebo's (2022) category of ‘consensus’ as a subcategory of ‘formal’ definitions.

To make our categorisation transparent and replicable, we used linguistic markers to identify the definitions potentially using these models. Subsequently, we screened manually all references to understand the context of the use of the linguistic marker. Table 9 presents the linguistic markers that we used, and an example of one definition that was coded in each category.

**Table 9:** Linguistic markers and examples of one definition per each category (animus, discriminatory selection, demonstration, teleological, consequentialist, formal).

Models	Linguistic markers	Example
Discriminatory selection	Based on, on the basis on, on grounds of, because of, due to, directed to, targeting, select*	[hate crime is] violence against individuals enacted on the basis of their membership in a social group as opposed to personal animus (Robertson, 2012)
Demonstration	Demonstrated, expressed, manifested	[hate crimes are] crimes that manifest prejudice based on certain group characteristics (Davis & Graham, 1995)
Animus	Motivated by, intended to, aiming to, designed to, hostility, hatred, prejudice, bias	[hate crime is] a criminal offence that is at least partially motivated by some form of identity‐based prejudice (Jensen et al., 2021)
Teleological		[hate speech is] a type of communication (not just verbal) which, addressing a large audience, intends to undermine the status of already fragile individuals or groups (translated from Italian language using Google Translate; Fumagalli, 2019)
Consequentialist	Harm*, damag*, inflict*	[hate speech is] statements that (re)produce prejudices and discriminate against marginalised groups. In contrast to (cyber) bullying, hate speech is always group‐related: the consequences of hate speech affect not only the people who have been harmed, but also entire social groups (e.g., Jews, migrants, people with disabilities and the like) (translated from German language using Google Translate; Quent, 2018)
Formal	Encompass*, expressed, manifested	[hate speech has] has the following main characteristics: ■ identifies public and denigrating expressions of thought intended to arouse a hostile, discriminatory or violent reaction or action on the part of the interlocutors; ■ incites discrimination, hostility or violence against an individual or a specific social group, identified on the basis of prejudices and negative stereotypes used as elements of differentiation that make inferiority with respect to the aggressor's group; ■ violates some fundamental human rights: the right to equality, to human dignity, to freedom, to participation in political and social life (translated from Italian language using Google Translate; Lunaria, 2019)

Importantly, the models are not mutually exclusive: one definition can include references to more than one model. The following is an example of a definition that we coded as containing ‘discriminatory selection’, ‘animus’ and ‘demonstration’ approaches:

Anti‐Roma hate crimes are criminal offences *motivated by* the bias of racism against Roma and Sinti, as well as various other people and groups considered associated with, or perceived as, Roma and Sinti, due to their actual or perceived ‘race’, ethnicity, language or migration status. The prejudice manifests itself either in the *selection of the target* (e.g., a Roma settlement) or in anti‐Roma racist hostility *expressed* during the crime (OSCE ODIHR, 2021a).

In relation to objective 1, we present the analysis of definitions contained in legislation in a separate section because they fundamentally differ from the definitions in academic and grey literature on important variables that affect our data coding, analysis and data interpretation. Firstly, definitions in legislation look different from definitions in academic and grey literature documents because some of them are implicit or use references to previous legislation, which require a different analytical approach. An example is: ‘No person shall violate section 2903.21, 2903.22, 2909.06, or 2909.07, or division (A)(3), (4), or (5) of section 2917.21 of the Revised Code by reason of the race, colour, religion, or national origin of another person or group of persons. (B) Whoever violates this section is guilty of ethnic intimidation’. (Ohio Revised Code § 2927.12, 2021). Secondly, for legislation, we coded unique variables such as the source of the legislation (e.g., whether civil, criminal, soft law), which are not comparable with academic or grey area documents. Thirdly, for academic and grey literature documents, we coded variables (which were key in our analytical approach) that are absent from legislation documents. Examples are: Google Scholar citations (as a proxy measure of uptake), publication date (which is less clear‐cut in legislation because of amendments that can subsequently change the content of a legislation. For these reasons, many of the analyses that we conducted for definitions contained in academic and grey literature documents are not replicable for definitions contained in legislation, which undermines the possibility of a full comparison and require a separate presentation of the results.

#### 4.7.2 Review objective 2: Measurement tools

The majority of the information that we collected about the measurement tools were qualitative and textual, and we attributed categorical codes to the text extracted. For each instrument, we coded tools, metrics and methods, stage of development and additional qualitative information (e.g., whether the instrument is adopted by any government or non‐government organisation).

Additionally, when reported, we extracted additional information about feasibility, efficacy, reliability and validity of the instrument. Broadly, validity and reliability refer to how well a measurement tool is able to measure a hate behaviour. Validity refers to the accuracy of a measurement tool. Reliability refers to the reproducibility of the results under the same conditions. Given the differences between the measurement tools under consideration, we selected different proxies to verify the validity and reliability of each measurement tool (see the next table; Table 10).

**Table 10:** Proxies of validity and reliability used in the data analysis.

Type of measurement tool	Validity proxy	Reliability proxy
Law enforcement data	Under reporting estimate (usually via comparison of victimisation survey data).	% of participation of local police forces in data collection system
Incidents reporting tools	Verification of the incidents reported.	Variation in reporting practices
Case records and open source databases	Bias present in the original data source (e.g., police data).	Bias in data collection process
Quantitative survey questionnaires	Validation analysis of the questionnaire used (e.g., face validity, content validity, predictive validity). Bias present in the data (e.g., sample bias).	Reliability analysis of the questionnaire used (e.g., test retest reliability)
Manual quantitative text analysis tools	Bias present in the original data source (e.g., social media data).	Inter rater reliability of coders (e.g., kappa)
Automated or semi‐automated text analysis tools	Bias present in the original data source (e.g., social media data)Precision, Recall, F1.	Inter rater reliability of coders (e.g., kappa)
Qualitative interviews schedules	Narrative discussion of the quality of the data collected (e.g., bias in the data and interview process).	Narrative discussion of replicability of the procedures used
Qualitative text analysis tools	Narrative discussion of the quality of the data collected (e.g., bias in the data and data collection process).	Narrative discussion of replicability of the procedures used

### 4.8 Data mapping

Tables and figures include a PRISMA diagram, as well as visual and numeric summaries of definitions and measurement tools. The report includes a narrative section where we interpret and contextualise the existing literature.

#### 4.8.1 Review objective 1: Definitions

The unit of analysis are the definitions. We present univariate analysis of the categorical data, as well as bivariate analysis of definition components and additional qualitative categories with document type, country, language, year. We conduct a social network analysis of the references in the documents containing the included definitions, to map disciplinary clusters and their relationships. We present a narrative description of definition components and additional qualitative categories.

#### 4.8.2 Review objective 2: Measurement tools

The unit of analysis are the instruments to measure hate speech, hate incidents, hate crimes or surrogate terms. We present univariate analysis of the data that we collected and coded as part of this review, which includes categorical data, as well as bivariate analysis of instruments, metrics, tools, methods and additional qualitative categories with document type, country, language, year. We present a narrative report describing instrument's characteristics, stage and additional qualitative categories, which is used to provide an assessment of the definitions, together with any available information about the instrument's reliability and validity.

### 4.9 Assessment and investigation of heterogeneity

The different types of definitions and measurement tools components are coded as described in Section 4.5. We devised a tailored data extraction approach to adapt and tailor our indicators to each type of definition (legislation vs. academic and grey area) and measurement tool.

### 4.10 Review author reflexivity

The team has a variety of disciplinary backgrounds that reflect different positions and approaches in the field of hate crime studies, including qualitative and theoretical sociology (Perry), quantitative criminology (Chermak and Freilich), mixed methods sociology and social psychology (Vergani), quantitative social psychology (Iqbal), mixed methods online research (Scrivens), data science (Betts), legal studies (Kleinsman). The team is well versed in relevant theory and in the study of subfields relevant to this review, such as Islamophobia, antisemitism, terrorism, and violent extremism. This review's aim is to map – not summarise or meta‐analyse – the existing definitions and measurement tools across different disciplines. The review team has been maintaining a reflexive position throughout all the stages of the review process, and decisions have been discussed critically and regularly among the team members with regular debriefing sessions to support with decision‐making and coding. The findings reveal a combination of approaches and disciplinary contributions across the whole spectrum of sciences relevant to the study of hate behaviours. The team remained mindful of conscious and unconscious presuppositions and supported each other to minimise the risk of these skewing our analysis or the interpretation of our findings.

## 5 RESULTS

### 5.1 Description of studies

#### 5.1.1 Results of the search

The systematic searches conducted between 28th March 2021 and 12th April 2022 yielded 35,191 references. We identified these references by searching across 19 databases (*N* = 32,443), experts' interviews (*N* = 115), websites (*N* = 2575) and citation searching (*N* = 58). A total of 13,366 duplicate references were removed before screening the titles and abstracts, which left 21,825 references. Following title and abstract screening, 14,621 references were excluded for varying reasons, such as not addressing hate crime, hate speech or hate incidents, or not focusing on a relevant country context or time period. This left 7204 references eligible for full‐text retrieval. Of these 7204 references, we could not retrieve 438 documents. These are mostly grey literature documents or theses published in the 1990s, which in most cases were not digitalised and were not available via our university libraries. We assessed the full text of 6766 references and we excluded a total of 6197 references because they did not meet our eligibility criteria (e.g., studies that did not contain an original definition or measurement tool). A total of 569 references were considered eligible for full‐text coding, containing 423 original definitions and 168 measurement tools. Importantly, our review identified 5267 documents addressing a relevant hate behaviour: of these, 41% (*N* = 2172) contained no definition or measurement tool, 48% (*N* = 2526) contained a definition and/or measurement tool that was not original, 8% (*N* = 402) contained an original definition, and 3% (*N* = 167) an original measurement tool in absence of an explicit definition. In parallel, we identified 714 legislation documents regulating hate behaviours in relevant contexts. They were all screened to search for relevant definitions: 631 documents were excluded, and 83 included. These documents contained 83 original definitions of relevant hate motivated behaviours.

**Figure 2:**
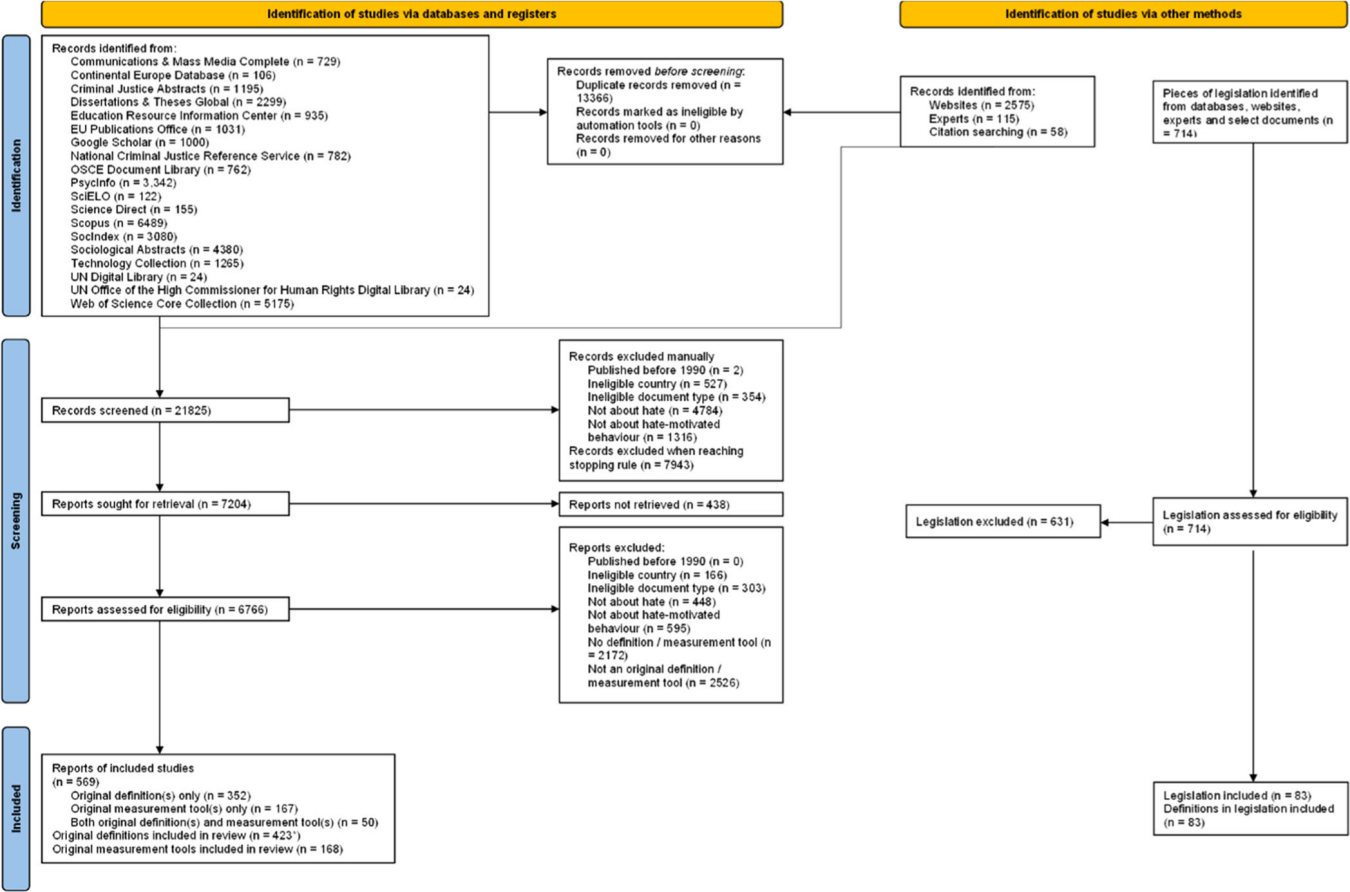
PRISMA flowchart. Data extraction of definitions from all reports of included studies yielded a total of 441 original definitions. Manual review of a consolidated dataset of all definitions extracted from academic and grey literature as well as legislation identified 18 duplicate definitions. Definitions extracted from legislation were kept over those extracted from academic and grey literature.

The PRISMA flowchart is in Figure 2.

#### 5.1.2 Included studies

We included 506 original definitions (of which 423 in academic and grey literature, and 83 in legislation documents) and 168 original measurement tools. The next table provides a comparative overview of the language of the included definitions and measurement tools (Table 11).

**Table 11:** Included original definitions and measurement tool by document language.

	Definitions	Measurement tools
English language	428	134
French language	10	12
German language	24	5
Italian language	26	8
Spanish language	18	9
Total	506	168

When considering country contexts, the USA are the focus of the largest group of documents where our definitions and measurement tools are found (Table 12).

**Table 12:** Country focus of the documents where our included definitions and measurement tools are found.

	Definitions	Measurement tools
USA	167	45
Canada	20	11
UK	67	26
Ireland	15	7
Spain	22	21
Italy	31	15
Germany	30	17
France	13	15
Australia	34	9
New Zealand	12	5
Other (e.g., internet, theoretical, UN, EU)	132	0

*Note*: One document can focus on multiple country contexts.

Each type of measurement tool included in the analyses was analysed separately to reflect the fundamental differences in their methodological approaches. The next table reports the key differences, aims and data used by each type of measurement tool (Table 13).

**Table 13:** The types of measurement tools considered in this review, their data sources and aims.

Type of measurement tool	*N* of included documents	Data source	Main aim
Law enforcement data	0	Law enforcement agencies	Measure the frequency of a hate behaviour
Incidents reporting tools	21	Victims' or witnesses' reports	
Case records and open source databases	26	Secondary data sourced from media or police accounts	
Quantitative survey questionnaires	53	Survey data	
Manual quantitative text analysis tools	14	Text and/or images from various sources	
Automated or semi‐automated text analysis tools	21	Text and/or images from digital media	
Qualitative interviews schedules	15	Semi structured interviews	Understand the different manifestations of a hate behaviour
Qualitative text analysis tools	18	Text and/or images from various sources	

#### 5.1.3 Excluded studies

As explained in the methods section, the main substantive exclusion criteria that we adopted in this review were originality, relevance and transparency. Firstly, we excluded documents that were not explicit in proposing an original definition of a relevant hate behaviour because, among the thousands of documents containing definitions of such behaviours, the majority merely replicate or slightly modify existing definitions from prior work, a common practice in academic and scientific literature. Consequently, in our mapping, we aimed to concentrate solely on original definitions in order to accurately map the range of different definitions and provide a comprehensive account of their extent and diversity.

Secondly, we excluded documents that did not focus on relevant behaviour because terms commonly encountered in this field, such as racism, antisemitism, or homophobia, are often used to describe both attitudes and behaviours. While acknowledging the relationship between attitudes and behaviours, we had to narrow our scope to behaviours due to feasibility considerations.

Thirdly, we excluded measurement tools that did not provide a transparent description of the research methods (i.e., data collection and data analysis) because, without a clear discussion of the methodology employed, we were unable to review and assess these tools effectively and determine if they met the criterion of originality.

Finally, we excluded official law enforcement data, as well as many incidents reporting tools run by government and nongovernment organisations, because the information about their data collection methodology available in open source documents is not enough for us to conduct a meaningful comparative assessment. As we explain in the methods section, a comprehensive analysis and comparative assessment of these measurement tools is much needed, but it would need a different methodological approach and research design than the systematic review methods (e.g., interviews with key stakeholders to retrieve information not in the public domain, content analysis of guidelines used by police officers and frontline workers capturing the hate behaviour, assessment of training materials).

### 5.2 Methodological limitations of included studies

The main methodological limitation of many of our included documents containing both original definitions and measurement tools is the low transparency in explaining how they distinguish between hate and non‐hate behaviours. While academic articles are usually required to provide a detailed methodological note – although reporting practices may vary significantly between disciplines and journals – grey area documents often lack the same level of detail. In some cases, we know anecdotally that certain government and nongovernment organisations possess detailed internal guidelines and methodological standards for the measurement of hate behaviours. However, these standards are absent – or only mentioned in passing – in the published reports that we included in our analysis. For this reason – especially in relation to measurement tools used by government and non‐government organisations – we are limited in in our ability to fully conduct a rigorous quality assessment as part of the scope and methodology of this systematic review.

#### 5.2.1 Limitations affecting the quality of the data collected by measurement tools

It is important to highlight some general methodological limitations that affect the quality of the data collected by most of the measurement tools that we included in our review. These limitations are usually not reported or discussed in the documents that we assessed, but they do affect the measurement of all hate behaviours. All the data originating from victims' and witnesses' reports (i.e., official data, incidents reporting tools, open source databases and case records) are hindered by under‐reporting and by variation of reporting practices within and across communities (Pezzella et al., 2019; Vergani & Navarro, 2023). The perceptions of what constitutes hate crime, hate speech or bias motivation varies by individual demographics and community contexts: the same behaviour can be interpreted differently depending on an individual's understanding of legislation and other individual and group factors. This is further exacerbated by limitations specific to types of measurement tools and country contexts. For example, in the USA local agencies report their hate crime data to the FBI on a voluntary basis, which means that the data are incomplete and uneven across states and local jurisdictions.

Other biases common to all social research activities apply to all the measurement tools (e.g., sample bias, coding bias, interviewer bias). However, these biases are rarely discussed and acknowledged in the documents that we reviewed. For example, victimisation surveys often fail to collect comprehensive data among some relevant groups of the population that are more likely to experience hate behaviours, such as young people (especially people younger than 18), minority groups, temporary visa migrants, asylum seekers and refugees, disabled people, the homeless, among others. Some surveys do not include questions about key demographic characteristics that preclude consideration of hate crimes against particular groups. For instance, the Department of Justice's Bureau of Justice Statistics conducts a regular National Crime Victimisation Survey (NCVS). The NCVS's exclusion of information on immigrant status precludes consideration of immigrant bias victimisation (Sheppard et al., 2021).

### 5.3 Mapping results

In this section we present our mapping of definitions and measurement tools. We structure the reporting of the results around the key research questions that we outlined for each objective (see Section Search Methods, 3).

#### 5.3.1 Objective 1: Mapping definitions of hate crime, hate incidents, hate speech, and surrogate terms

As outlined in out Methods section, we map separately definitions found in academic and grey literature (*N* = 423) and definitions found in legal documents (*N* = 83).

##### RQ1. How are hate crimes, hate speech and hate incidents defined in the academic, legal, policy, and programming literature?

To reflect the title and aims of our review, we coded whether the 423 included definitions in grey area and academic literature capture a behaviour that we could define as a ‘hate crime’, ‘hate speech’ or a ‘hate incident’. Around 38% of our included definitions (*N* = 159) define ‘hate crime’, 27% (*N* = 116) ‘hate speech’, 30% (*N* = 127) behaviours across multiple boundaries, and 5% (*N* = 21) ‘hate incidents’. This distribution reflects the fact that a large portion of our sample included documents informed by statutory and related regulatory mechanisms, which by definition only look at criminal behaviours. It may also reflect some of the academic materials that tend to define crime generally and hate crime specifically as ‘social harms’ that extend beyond legal definitions. The comparatively large group of definitions focusing on behaviours that are situated across the boundaries of hate speech, hate incidents and hate crime suggests that there is a significant part of the literature that aims to understand the dynamic relationships between different manifestations of hate including criminal and non‐criminal behaviours like microaggressions and hate speech. Schweppe and Perry (2022) have defined this field as being the study of the ‘continuum of hate’.

##### RQ2. What are the concepts, parameters and criteria that qualify a behaviour as being hate crime, hate incident or hate speech?

*Hate qualifier (e.g., ‘hate’, ‘bias’, ‘prejudice’, ‘racist’)*. The most used term to define the ‘hate’ qualifier in our original definitions is ‘hate’ (used in about 58% of the definitions, *N* = 246). This confirms that – although the term is ambiguous, normative and emotive – it is the most widely adopted. The second most used term is ‘racist’ (10%, *N* = 40), which shows that a sizeable portion of the literature focuses exclusively on racist behaviours. The rest of the literature is highly fragmented with many different terms used in a small number of definitions. Examples are: ‘bias’ (*N* = 15), ‘antisemitic’ (*N* = 15), ‘gender‐based’ (*N* = 12), ‘Islamophobic’ (*N* = 11), ‘homophobic’ (*N* = 6), ‘transphobic’ (*N* = 4), ‘misogynistic’ (*N* = 3), ‘afrophobic’ (*N*‐ = 2), ‘xenophobic’ (*N* = 2), among others.

*Behaviour (e.g., ‘crime’, ‘speech’, ‘incident’, ‘violence’)*. The most used terms to capture the behaviour defined in our original definitions are ‘crime’ (36%, *N* = 151) and ‘speech’ (23%, *N* = 98), which reflect the two main foci of our definitions (i.e., ‘hate crime’ and ‘hate speech’). The other terms reflect a high fragmentation of the terminology adopted in the literature, which was already captured in previous work (Vergani & Link, 2021). This fragmentation of terminology also reflects both community‐specific research (using ‘isms’ and ‘phobias’ like racism, antisemitism, homophobia, transphobia), as well as the debate in the hate crime literature about whether hate crime definitions should only capture criminal acts or not. For example, although most definitions of hate crime adopted by government organisations only include criminal acts, academics like Chakraborti and Garland (2015a) propose to define hate crimes as ‘acts of violence’, hostility and intimidation directed towards people because of their identity or perceived ‘difference’ (Chakraborti & Garland, 2015a, p. 5), which includes both criminal and non‐criminal behaviours.

*Targets (e.g., ‘groups’, ‘persons’, ‘properties’, ‘organisations’, or a combination of those)*. About 67% of our original definitions (*N* = 282) listed explicitly the targets of the hate behaviour as either ‘groups’, ‘persons’ or ‘individuals’, ‘properties’, ‘organisations’ or a combination of those. About 17% (*N* = 72) listed only groups, 20% (*N* = 83) only individuals or persons, 18% (*N* = 74) individuals and groups, and 13% (*N* = 53) a more precise combination of targets including properties, organisations, institutions, symbols, among others.

*Protected characteristics (e.g., ‘race’, ‘religion’, ‘sexual orientation’)*. We counted over 30 different protected characteristics named in our original definitions, which we grouped into six categories: ‘ethnic and religious identities’, ‘gender and sexual identities’, ‘disability, body and health’, ‘social class’, ‘ideology and occupation’. We acknowledge that within each of these categories there are significant differences in the target groups: for example, misogyny and anti‐ LGBTQIA+ hatred are merged into ‘gender and sexual identities’, ageism and ableism into ‘disability, body and health’, among others. Our choice was guided by the attempt to identify categories with as little overlap as possible. For example, many definitions list ‘gender’ as a protected characteristic, covering both anti‐women and anti‐transgender hate, making it impossible to distinguish neatly between the two. They are summarised in the following table (Table 14).

**Table 14:** Protected characteristics by group.

Category	Percentage of definitions protecting the category	Examples of terms used in the definitions
Ethnic and religious identities	59% (*N* = 249)	Race, ethnicity, nationality, religion, colour, descent, migrants, culture, language, Roma, Jews, Muslims, caste
Gender and sexual identities	47% (*N* = 200)	Gender identity, gender expression, sexual orientation, gay, lesbian, bisexual, transgender, sex, gender, misogyny, women
Disability, bodies and health	25% (*N* = 105)	Disability, mental and physical disability, health status, physical appearance, age
Ideology and occupation	4% (*N* = 15)	Political ideology, political identity, opinion, occupations (police, judge, journalists)
Social class	3% (*N* = 14)	Class, social status, poverty, homelessness

Some of the terms used to define the protected characteristics have overlapping areas of meaning, but they are conceptually different (e.g., ‘race’, ‘skin colour’, ‘ethnicity’, ‘nationality’, ‘culture’). About 29% (*N* = 121) of our definitions did not list any protected characteristic, and about 23% (*N* = 98) listed only one protected characteristic. The remaining group of definitions (48%, *N* = 204) listed two or more protected characteristics. About 59% (*N* = 58) of the definitions listing only one protected characteristic focus on ethnic and religious identities, 33% (*N* = 32) on gender and sexual identities, 8% (*N* = 8) on disabilities, bodies and health, 2% (*N* = 2) on social class and 2% (*N* = 2) on ideology and occupation. About 94% (*N* = 191) of the definitions listing two or more protected characteristic focus on ethnic and religious identities, 83% (*N* = 169) on gender and sexual identities, 49% (*N* = 100) on disabilities, bodies and health, 8% (*N* = 17) on social class and 9% (*N* = 18) on ideology and occupation. We compared the protected characteristics between hate crime and hate speech definitions listing 2 or more protected characteristics (*N* = 204), and found a few statistically significant differences. Specifically, 90% of hate crime but only 80% of hate speech definitions include gender and sexual identities; 60% of hate crime but 39% of hate speech definitions include ‘disability’.

###### How is the ‘hate element’ conceptualised in the original definitions?

Among definitions of hate crime (*N* = 159), about 74% (*N* = 117) used the ‘animus’ model, 42% (*N* = 66) the ‘discriminatory selection model’, and 9% (*N* = 14) the ‘demonstration’ model.

Among definitions of hate speech, hate incidents and behaviours across multiple boundaries (*N* = 275), about 36% (*N* = 94) adopt a ‘teleological’ model, 14% (*N* = 37) a ‘consequentialist’ model, and 53% (*N* = 140) a ‘formal’ model.

##### RQ3. What are the most common concepts, parameters and criteria found across definitions? What are the differences between definitions and the elements they contain?

We detected trends over time, across geographical areas and between bodies of literature, which help us understand the most common concepts, parameters and criteria found across definitions.

###### Trends over time

There was an overall increase in the number of original definitions that we found in the literature. Only about a third (31%) originate from documents published between 1990 and 2009. About two thirds are found in documents published between 2010 and 2021. This suggests an increasing interest in the topic of hate behaviours over time. We see the growth of definitions as a good proxy for growth of research and policy interest in hate behaviours.

We detected an increase over time in the focus on new protected characteristics. Only 7% of the definitions including ‘social class’ as protected characteristics are in documents published in the decade 1990–1999, and 21% are in documents published in 2020 and 2021. Only 20% of definitions including ‘ideology and occupation’ identities as protected characteristics are in documents published in the decade 1990–1999, and 33% are in documents published in 2020 and 2021. Caste emerged as a protected characteristic in 2020 and 2021 (in 3 definitions). We found an increasing number of definitions of ‘hate speech’ published in recent years, which demonstrates a growing global interest about this concept. About 10% of hate speech definitions are found in documents in the decade 1990–1999, 14% in documents published between 2000 and 2009, 47% between 2010 and 2019, and 28% in 2020 and 2021.

###### Geographical and language differences

About a third of the definitions (33%, *N* = 138) originated from documents focusing on – and for the most part, originating from – North America (i.e., United States or Canada). The majority of the definitions originate from documents in English language (about 83%, *N* = 349). Importantly, we found that the geographical distribution of the documents containing original definitions changed over time. Of the 54 original definitions published between 1990 and 1999, 85% (*N* = 46) originated from North America. Of the 105 original definitions published in 2020 and 2021, only 12% (*N* = 13) originated from North America. This suggests that the contributions to this body of literature from regions other than North America became more significant over time. While 42% (*N* = 66) of the original definitions of ‘hate crime’ are found in documents focusing on North America, only 23% (*N* = 27) of definitions of ‘hate speech’ and 27% (*N* = 34) of definitions of behaviours covering multiple boundaries are found in documents focusing on North America. We found a significant difference between Anglophonic countries (US, Canada, UK, Ireland, Australia, New Zealand) and continental Europe (Italy, Spain, Germany and France). Specifically, 82% of hate crime definitions, but only 69% of definitions focusing on hate speech, incidents and multiple categories, originate from documents focusing on Anglophonic countries.

###### Differences between academic and grey literature

About 56% (*N* = 237) of our definitions are found in the academic literature and 44% (*N* = 186) in the grey literature. This result confirms that hate behaviours are a ‘policy domain’ (Farrell & Lockwood, 2023) of great interest for government and nongovernment organisations. Definitions in the grey literature – compared to the ones in the academic literature – contain more protected characteristics. On average, academic definitions list 2.5 and grey literature definitions 3.5 protected characteristics. Definitions in the grey literature also provide a more precise descriptions of the targets of the hate behaviours (e.g., ‘individuals’, ‘properties’ and ‘organisations’). About 30% (*N* = 72) of academic definitions, but only 26% (*N* = 49) of grey literature definitions, do not identify any protected characteristics. Definitions adopting an ‘animus’ model (i.e., based on the malicious ‘intent’ of the offender) are more common in the grey (80%, *N* = 71) than in the academic literature (66%, *N* = 46). This possibly reflects a ‘culture’ or ‘habit’ in government and non‐government organisations to consider only the animus approach to identify the hate element, and to rely on bias indicators and victims' perceptions. About 56% (*N* = 89) of hate crime definitions are found in the grey literature. However, only 25% (*N* = 29) of the definitions of hate speech are found in the grey literature. This difference is likely a reflection of the interest of government organisations such as law enforcement agencies in hate crime, which make up a significant part of the grey literature. We interpret our findings as identifying a tension between the need to operationalise definitions into measurable components (found mainly in the grey literature) and the need to discuss drivers and motives of hate behaviours (found in the academic literature).

###### Social network analysis

To map with more precision the fragmentation of the literature that ewe mapped, we conducted a social network analysis of the references cited in the documents containing our original definitions as well as our original measurement tools. We used the Semantic Scholar API to obtain information about the documents that had a DOI. The Semantic Scholar API returns a wide variety of information about a particular document, but we were primarily interested in the list of authors, field of study, and list of references. For documents that did not have a DOI, we manually searched their titles on the Semantic Scholar website, noting down their Corpus ID, before using to the Semantic Scholar API to search for these papers by Corpus ID. We then repeated the above processes to obtain information on the references, primarily to obtain their field of study. We found referencing data for a total of 325 documents (out of the total 569).

Of these 325 documents, 98 are categorised as social sciences (e.g., sociology, political science, criminology), 47 are health (including psychology and medicine), 23 are STEM (e.g., computer science, Mathematics, Engineering, etc.), 2 are humanities (e.g., history, philosophy), and 14 are in other. In terms of referenced documents, the top five discipline groupings are social science with 2786 documents cited within the network, followed by health (1612 documents), STEM (993documents), humanities (289 documents), and other (252 documents). In terms of citation counts (as opposed to the number of documents), the top 5 most cited fields follow a similar pattern. First is social science (3279 citations), followed by health (1860 citations), STEM (1393 citations), humanities (311 citations), and other citations.

**Figure 3:**
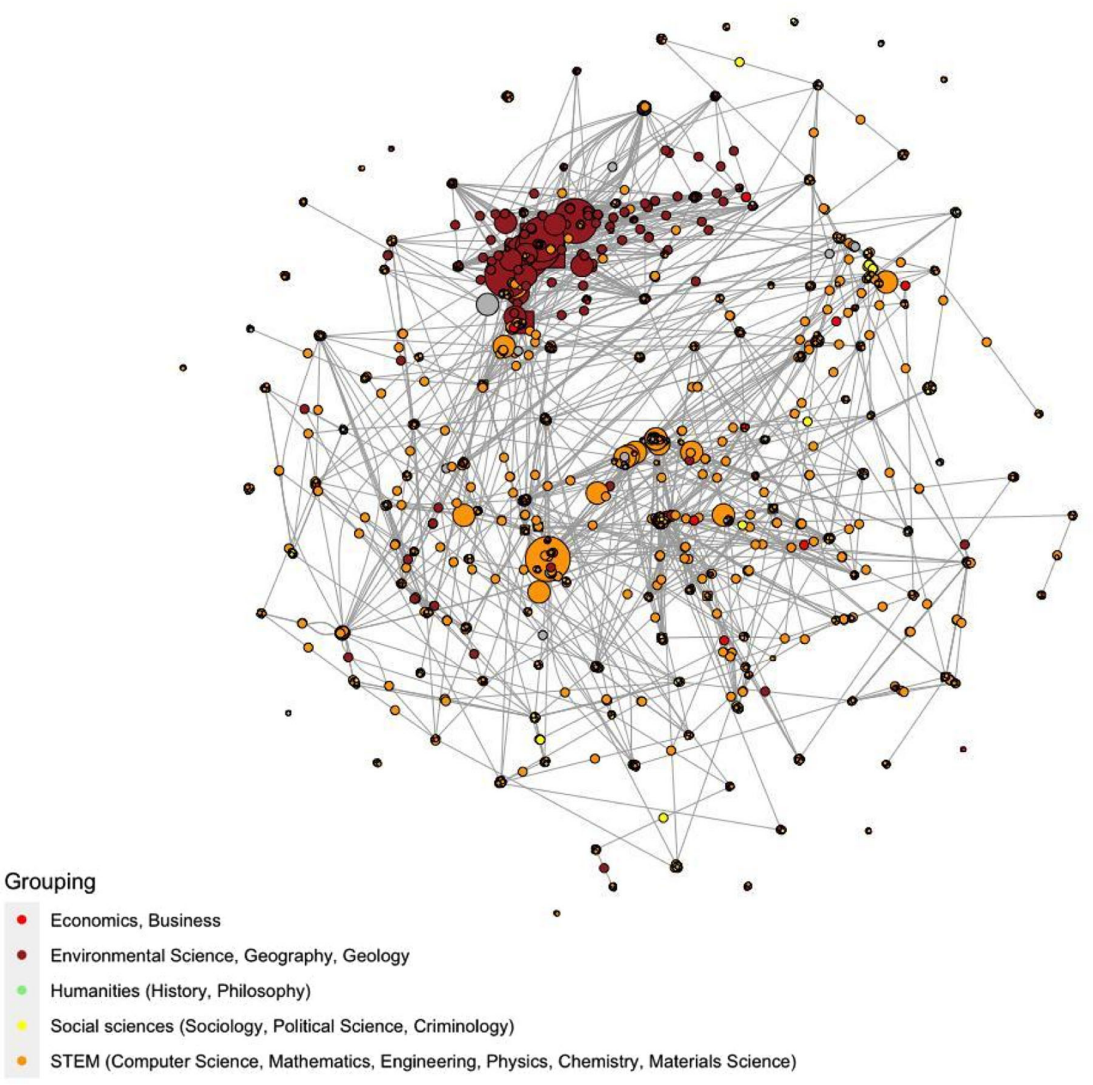
Social network analysis of the documents included in our review.

The next figure shows a social network map of documents within our literature review and their citations. The size of the nodes indicates how often a document is cited within the network, with bigger nodes meaning more citations. Square nodes are papers originally included within the literature review (henceforth referred to as original documents), while circle nodes are documents that were cited by the original documents within the literature review (henceforth referred to as references) (Figure 3).

Within the social science citations, 67% (2211) of those came from social science, while 20% came from health (psychology, medicine). Five percent came from STEM and 8% came from other. In the health grouping, 49% came from social sciences, 39% from other health sources, 7% from STEM, and 5% from other. For STEM, 55% of the citations came from STEM, while 19% are from social science documents, followed by health (16%), other (10%).

Out of the total 325 literature review documents that we had referencing data for, the most cited paper was ‘Automated Hate Speech Detection and the Problem of Offensive Language’ (2017), a computer science paper that was cited 15 times by original documents, 12 of which are by other computer science documents. The most cited literature review document was ‘Deep Learning for Hate Speech Detection in Tweets’ (2017), which was a computer science paper cited 11 times, 8 of which are citations by other computer science documents.

Further highlighting the fragmented nature of the network, on average a STEM original document will reference 33 other computer science documents, followed by 9 social science and 7 health and 2 humanities papers each. Similarly social science, will reference an average of 24 other social science papers, 11 medicine papers, five humanities papers, and five STEM papers. The trend of citing mostly other papers within the same field is also true of medicine (13 papers) and humanities (7 papers).

##### Mapping definitions found in legislation

We identified 714 pieces of legislation regulating hate behaviours in relevant country contexts. After screening all these documents, we identified 83 definitions of behaviours that we classified as being either hate crime (60%, *N* = 50) or hate speech (40%, *N* = 33). About 63% (*N* = 52) are found in criminal law, 21% (*N* = 17) in civil law, 8% (*N* = 7) in soft law (e.g., recommendations and frameworks), 7% (*N* = 6) in supplementary materials (e.g., Camden principles), and one in international law (i.e., conventions that create obligations against which states can be held accountable). In the next table, we provide examples of hate crime and hate speech definitions found in different repositories (Table 15).

**Table 15:** Examples of hate crime and hate speech definition by source (e.g., criminal law, civil law).

	Hate speech definition (example)	Hate crime definition (example)
Criminal law	Hate propaganda means any writing, sign or visible representation that advocates or promotes genocide or the communication of which by any person would constitute an offence under section 319; (propagande haineuse) (R.S.C. Criminal Code 1985, s. 320)	For the purposes of this section, an offence is aggravated by religious prejudice if – (a) at the time of committing the offence or immediately before or after doing so, the offender evinces towards the victim (if any) of the offence malice and ill‐will based on the victim's membership (or presumed membership) of a religious group, or of a social or cultural group with a perceived religious affiliation; or (b) the offence is motivated (wholly or partly) by malice and ill‐will towards members of a religious group, or of a social or cultural group with a perceived religious affiliation, based on their membership of that group. (Criminal Justice (Scotland) Act, 2003, s 74(2A))
Civil law	Transgender vilification unlawful (1) It is unlawful for a person, by a public act, to incite hatred towards, serious contempt for, or severe ridicule of – (a) a person on the ground that the person is a transgender person, or (b) a group of persons on the ground that the members of the group are transgender persons. (2) Nothing in this section renders unlawful – (a) a fair report of a public act referred to in subsection (1), or (b) a communication or the distribution or dissemination of any matter on an occasion that would be subject to a defence of absolute privilege (whether under the Defamation Act 2005 or otherwise) in proceedings for defamation, or (c) a public act, done reasonably and in good faith, for academic, artistic, scientific, research or religious discussion or instruction purposes or for other purposes in the public interest, including discussion or debate about and expositions of any act or matter (Anti‐Discrimination (NSW) Act, 1977, s. 38S)	N/A
Soft law	The term ‘hate speech’ shall be understood as covering all forms of expression which spread, incite, promote or justify racial hatred, xenophobia, anti‐Semitism or other forms of hatred based on intolerance, including: intolerance expressed by aggressive nationalism and ethnocentrism, discrimination and hostility against minorities, migrants and people of immigrant origin (Recommendation No. R (97) 20 of the Committee of Ministers to Member States on ‘Hate Speech’)	Hate crimes – i.e., violence and crimes motivated by racism, xenophobia, anti‐Gypsyism, anti‐Semitism or religious intolerance, or by a person's sexual orientation, gender identity or membership of a minority group, or on the basis of the non‐exhaustive grounds listed in Article 21 of the Charter of Fundamental Rights (European Parliament Resolution of 14 March 2013 on Strengthening the Fight Against Racism, Xenophobia and Hate Crime (2013/2543(RSP)))
Supplementary materials	In the context of this document, the term hate speech is understood as any kind of communication in speech, writing or behaviour, that attacks or uses pejorative or discriminatory language with reference to a person or group on the basis of who they are, in other words, based on their religion, ethnicity, nationality, race colour, descent, gender or other identity factors (UN Strategy and Plan of Action on Hate Speech)	Hate crimes are criminal acts committed with a bias motive. It is this motive that makes hate crimes different from other crimes. A hate crime is not one particular offence. It could be an act of intimidation, threats, property damage, assault, murder or any other criminal offence. The term ‘hate crime’ or ‘bias crime’, therefore, describes a type of crime, rather than a specific offence within a penal code. (OSCE Office for Democratic Institutions and Human Rights, 2009)
International law	Hatred' should be understood as referring to hatred based on race, colour, religion, descent or national or ethnic origin (Council Framework Decision 2008/913/JHA of 28 November 2008 on Combating Certain Forms and Expressions of Racism and Xenophobia by Means of Criminal Law)	N/A

About 34% (*N* = 17) of hate crime definitions uses an ‘animus’ approach, and about 58% (*N* = 29) uses a ‘discriminatory selection’ approach, about 26% (*N* = 13) use a ‘demonstration’ and ‘animus’ approach combined. About 69% (*N* = 22) of hate speech legislation adopt a teleological approach, about 31% (*N* = 10) a consequentialist approach, and about 28% (*N* = 9) a formal approach.

While the discriminatory selection model is most commonly used in criminal law (*N* = 28), there are also a number of definitional provisions using the animus model (*N* = 16), and some using both the demonstration and motivation iterations of that model (*N* = 12), which appears to create the greatest scope for application, by providing for the explicit demonstration of animosity, as well as for a motivation of this nature to be inferred. Interestingly, 10 of these provisions (using both the demonstration and motivation iterations of the animus model) are from the United Kingdom; one from Australia and one from the United States.

We coded the protected characteristics covered in the 83 original definitions included in our analysis. About 82% (*N* = 68) included race, 70% (*N* = 58) religion, 46% (*N* = 38) sexual orientation, 42% (*N* = 35) disability, 31% (*N* = 26) gender identity, 19% (*N* = 16) sex, 16% (*N* = 13) age, 5% (*N* = 4) political ideology, 4% (*N* = 3) occupation, and 4% (*N* = 3) homelessness.

There are statistically significant differences in the protected characteristics covered by definitions of hate crime and hate speech. About 90% (*N* = 45) of the definitions of hate crime, but only 70% (*N* = 23) of hate speech include race. About 82% (*N* = 41) of the definitions of hate crime, but only 52% (*N* = 17) of hate speech include religion. About 60% (*N* = 19) of the definitions of hate crime, but only 24% (*N* = 7) of hate speech include sexual orientation. About 58% (*N* = 29) of the definitions of hate crime, but only 18% (*N* = 6) of hate speech include disability.

The legislation in which the definitions are found addresses in 52% of the cases (*N* = 43) a physical criminal act, in 40% (*N* = 33) incitement to violence or hostility (or other negative behaviours), in 37% (*N* = 31) harassment, in 36% (*N* = 30) expressions or communications of hatred, in 8% (*N* = 7) use of hate symbols (e.g., swastikas, cross‐burning), in 7% (*N* = 6), denial of crimes against humanity, in 6% (*N* = 5) bestial depictions of protected groups, in 5% (*N* = 4) vandalism or disturbance, in 4% (*N* = 3) membership of hate groups.

**Figure 4:**
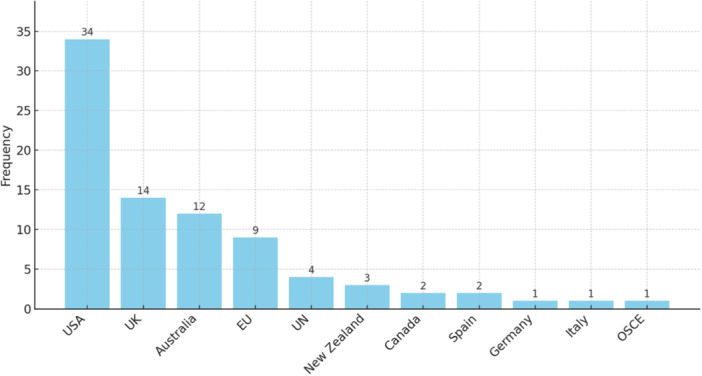
State or multinational body of the legislation where the definitions are found.

The next figure illustrates the origin of the legislation where the included definitions are found (Figure 4).

By looking at the differences between Anglophonic countries (USA, UK, Canada, Australia, New Zealand, Ireland) and continental Europe (Spain, Germany, Italy, and the EU), we found that about 90% (*N* = 45) of hate crime definitions, but only 60% (*N* = 20) of hate speech definitions, originate form Anglophonic countries. Conversely, only 8% (*N* = 4) of hate crime definitions, but 27% of hate speech (*N* = 9) definitions originate from continental Europe.

#### 5.3.2 Objective 2: Mapping the tools used to measure the prevalence of hate crime, hate incidents, hate speech, and surrogate terms

##### RQ1. How are definitions operationalised to measure hate crimes, hate speech, and hate incidents?

We included 168 original measurement tools. Of them, 24% (*N* = 41) measure hate crimes, 41% (*N* = 69) measure hate speech, 2% (*N* = 4) measure hate incidents, and 32% (*N* = 54) measure behaviours including hate crime, hate speech and hate incidents.

###### Law enforcement data

As detailed in the methods section, our 168 original measurement tools do not include documents capturing law enforcement data because the information about this data source is often not in the public domain. Therefore, the methodology of a systematic review is not suited for a comprehensive and comparative mapping and assessment of law enforcement data. However, we find it important to provide a brief narrative contextualisation of law enforcement data in this section of the report, before discussing our original findings about other measurement tools. The information presented here is found in official reports (e.g., European Union Agency for Fundamental Rights, 2018a, 2018b) as well as private communications with law enforcement officials.

Official law enforcement data are collected by police or other law enforcement agencies. Usually, police officers code and label the offence and determine whether or not a crime presents a ‘hate element’ (e.g., through a ‘discriminatory selection’, ‘demonstration’ and/or ‘animus’ approach), and indicate the type of motivation based on information gathered during the investigation and (when available) guidelines for case classification. Often, the coding is based on victims' perceptions and objective facts and circumstances indicating a bias motivation (i.e., bias indicators). All official law enforcement data sources capture hate crimes, that is, a crime recognised by the law that is committed with an additional hate element. Some law enforcement official data sources capture hate speech, that is, an expression act that is criminalised in a certain jurisdiction and that – without the hate element – would not constitute a criminal behaviour.

The next table presents an overview of the protected characteristics covered by law enforcement data collection systems in the countries under investigation. A more detailed narrative discussion of each county model follows the table (Table 16).

**Table 16:** Protected characteristics covered by official law enforcement data collection by country.

Country	Protected characteristics covered by official law enforcement data collection
USA	Race/ethnicity/ancestry, religion, disability, sexual orientation, ethnicity, gender, gender identity
Canada	Race, colour, national or ethnic origin, religion, sexual orientation, gender identity or expression, language, sex, age, or mental or physical disability
Germany	Nationality, ethnic origins, skin colour, religion, beliefs, physical and/or psychological disability or impairment, sexual orientation and/or sexual identity, political position, political views and/or political involvement, appearance, or status in society
UK	Race or ethnicity; religion or beliefs; sexual orientation; disability; and transgender identity
France	Religion, racism, xenophobia, sexism and homophobia
Ireland	Race, sexual orientation, nationality, religion, ethnicity, colour, gender, disability, age
Italy	Race/skin colour; ethnicity; nationality; language; anti‐Semitism; bias against Roma and Sinti; bias against Muslims; and bias against members of other religions, sexual orientation and transgender identity, and bias against people with a disability
Spain	Racism/xenophobia; ideology; sexual orientation or gender identity; sex/gender discrimination; religious beliefs or practices; anti‐Semitism; disability; aporophobia; anti‐Roma; illness; and general discrimination
New Zealand	Race, religion, sexual orientation, gender identity, disability or age
Australia	Religion, race, sex, age, disability, sexual identity, gender identity, homelessness, political activity (Victoria). Race, religion or faith, ethnic/national origin, sex or gender, LGBTQA+, mental or physical disability, political, homelessness, age, HIV/AIDS status (New South Wales)

*USA*. In the USA, in 1990 Congress passed the Hate Crime Statistics Act, modified by the Matthew Shepard and James Byrd, Jr., Hate Crimes Prevention Act. The act mandated the Federal Bureau of Investigation's (FBI's) Uniform Crime Reporting (UCR) Programme to compile aggregate hate crime data submitted by local police agencies. The UCR Programme defines hate crime as a criminal offence motivated, in whole or in part, by the offender's bias against the protected characteristics (Federal Bureau of Investigation, 1998). In 2021, the FBI finalised the process of transitioning to the National Incident‐Based Reporting System (NIBRS), which captures details about every crime incident – as well as about separate offences within the same incident – including information on victims, known offenders, relationships between victims and offenders, arrestees, and property involved in crimes. The NIBRS data is available in a web‐based platform that allows users to interrogate and download hate crime data. To our knowledge, there is no comparable initiative globally that achieves the same level of detail, data availability and transparency. However, because of Covid and other issues the transition has been affected by reduced reporting, which in 2022 triggered substantial public discussion and criticism of the NIBRS system (e.g., Miller‐Idriss, 2022).

*Canada*. In Canada since 2004 hate crimes collected by law enforcement appear in the Uniform Crime Reporting (UCR) Survey, which contains three databases– the incident file, the accused, and the victim file. Hate crimes refer to criminal incidents that, upon investigation by police, are found to have been motivated by hatred toward an identifiable group, as defined in subparagraph 718.2(a)(i) of the Criminal Code of Canada. Additionally, in Canada there are four specific offences listed as hate propaganda offences in the Criminal Code of Canada: advocating genocide, public incitement of hatred, wilful promotion of hatred and mischief motivated by hate in relation to religious property, and wilful promotion of antisemitism. These offences are also recorded in Canadian law enforcement data sources.

*UK*. In the UK, hate crime data collection is also compulsory and incorporated in the general crime recording system. Since 2007 there is one shared definition of hate crime shared by all agencies that make up the criminal justice system (including police, Crown Prosecution Service [CPS], Prison Service; Crown Prosecution Service, n.d.). According to this definition, which underpins federal data collection efforts, a hate crime is ‘any criminal offence which is perceived, by the victim or any other person, to be motivated by hostility or prejudice towards someone based on a personal characteristic’. The Police and two civil society organisations involved in monitoring hate incidents (Community Security Trust and Tell MAMA) have entered into Information Sharing Agreements, thereby enabling the exchange of data about incidents recorded by each organisation and providing a more holistic picture of hate crime (Feldman & Littler, 2014).

*Ireland*. In Ireland, law enforcement hate crime data collection is compulsory and incorporated in the general crime recording system. In the absence of a hate crime legislation, the current approach to recording hate crime in Ireland is through the identification of a base offence, which is then assigned to one or more bias motivations. Speech acts regulated by the Prohibition of Incitement to Hatred Act of 1989 are also recorded. The data is provided to the Central Statistics Office by the An Garda Síochána (the National Police Force of Ireland; An Garda Síochána, n.d.).

*Germany*. Germany's law enforcement agencies categorise hate crimes under the umbrella of politically motivated crimes (Bundesministerium des Innern, für Bau und Heimat, 2018). For statistical purposes, criminal offences are classified as hate crimes if there are indications that they are directed against a person on the basis of a list of protected characteristics. Using this definition, the police collects data nationwide using a shared code of practice, guidelines and instructions. In January 2018, the first Länder Judicial Administration began collecting statistical data on hate crimes. On 1 January 2019, the data collection was implemented nationwide.

*France*. Depending on the type of offence regulated in the criminal code, bias motivations can be recorded by law enforcement agencies. Since 2008, antisemitic and anti‐Muslim offences are also collected. The Ministry of the Interior is responsible for data collection processes. All security forces are connected to a central registration system, and data on hate crimes can be extracted from this database using the criminal qualification code under which they have been recorded (European Union Agency for Fundamental Rights, 2018a).

*Spain*. In Spain, law enforcement hate crime data collection is not compulsory, but incorporated in the general crime recording system as part of the Statistical Crime System (SEC). The SEC allows the recording of the presence of a discriminatory motivation of a crime. In addition to their criminal qualification or alleged violation of administrative rules, officers describe the criminal context when recording the crime, including ‘polarisation indicators’ pointing at a discriminatory motivation behind the criminal conduct (Secretaria de Estado de Seguridad, 2013).

*Italy*. In Italy, law enforcement hate crime data collection is not incorporated in the general crime recording system. All crime reports, including victim information and information about police action and legal qualification, are entered into and stored in the Sistema di Indagine (SDI) database. However, in this database it is possible to register only hate crime strands mentioned in the law, including ethnicity, nationality, race, religion, or crime against national linguistic minorities. Crimes committed on discriminatory grounds against gender identity or sexual orientation are entered into the SDI as ordinary offences but there is no way to mark them as bias motivated. In 2010 a separate system called Observatory for Security against Discriminatory Acts (OSCAD) has been established to monitor such crimes, but not all reports collected by OSCAD are included in the SDI database.

*Australia*. In Australia, there is no law enforcement data collection of hate crime at the federal level, because there is no relevant federal legislation and no definition of hate crime shared by different states (Vergani & Link, 2021). In some states, law enforcement agencies collect hate crime data, but they use different definitions, terminologies and approaches. For example, the New South Wales police force collects data about bias crimes, and Victoria Police collect data about prejudice‐motivated crimes. Data collection is uneven and patchy within states. For example, in New South Wales, under‐resourcing of the bias crime unit, inconsistencies in coding and changes in the way bias crime is recorded limit the quality of the data and preclude detailed analyses of trends over time (Mason, 2019).

*New Zealand*. In New Zealand, since 2019 police have been using flags in their IT systems to identify potential reports of hate crime. The markers of prejudice can be assigned either at the point of report or when those reports are recorded in the National Intelligence Application (NIA) as offences (hate crime) or non‐crime matters (hate incident). There are no specific legislative provisions in New Zealand to regulate hate crime offences, and data collection operates using a perception based approach. Hate crime data is currently not available for the public, but only for internal purposes. Following the terrorist attack that took place in Christchurch on 15 March 2019, the police conducted extensive consultations to improve its hate crime data collection practices as recommended in the Royal Commission of Inquiry Report (Royal Commission of Inquiry into the Attack on Christchurch Mosques on 15 March 2019, 2020).

In the next table, we provide a comparative overview of the main elements of hate crime data collection frameworks (Table 17). For federal countries, we consider the federal level frameworks. For example, in the USA the reporting of hate crime via the crime reporting system is voluntary, and many local police forces don't participate, so we consider the system not compulsory. In Australia, some states collect hate crime data (e.g., Victoria, New South Wales), but others don't, and there is no federal mechanism of reporting, and therefore hate crime data at Australian level is missing.

**Table 17:** Hate crime data collection country frameworks (adapted from European Union Agency for Fundamental Rights, 2018a).

	Flagging of hate crime/bias motivation is compulsory and integrated in the general crime recording system	Flagging of hate crime/bias motivation is not compulsory but incorporated in the general crime recording system	Flagging of hate crime/bias motivation is not incorporated in the general crime recording system	Flagging of hate crime/bias motivation is recorded separately from the general crime recording system	Police hate crime data are collected and published	Police hate crime data are collected by not published	Hate crime data are not collected in all states
USA		x			x		
Canada	x				x		
UK	x				x		
Ireland	x				x		
Germany				x	x		
France				x	x		
Spain					x		
Italy			x			x	
Australia			x				x
New Zealand			x			x	

###### Incident reporting tools (IRTs)

IRTs are usually a web‐based form, sometimes in conjunction with an email and/or a phone number, that victims or witnesses can use to report a hateful act to government and/or nongovernment organisations. The web forms usually have various open‐ended and multiple choice questions that structure and standardise the reporting process, which mirror the categories asked by operators via email or via phone. We found over 90 IRTs in the geographical areas under investigation. We propose that these IRTs can be divided into four categories, based on two dimensions:

1. whether they capture exclusively an online hate behaviour or a mix of offline and online hate behaviours;
2. whether they are the initiative of a government or nongovernment organisation.

**Figure 5:**
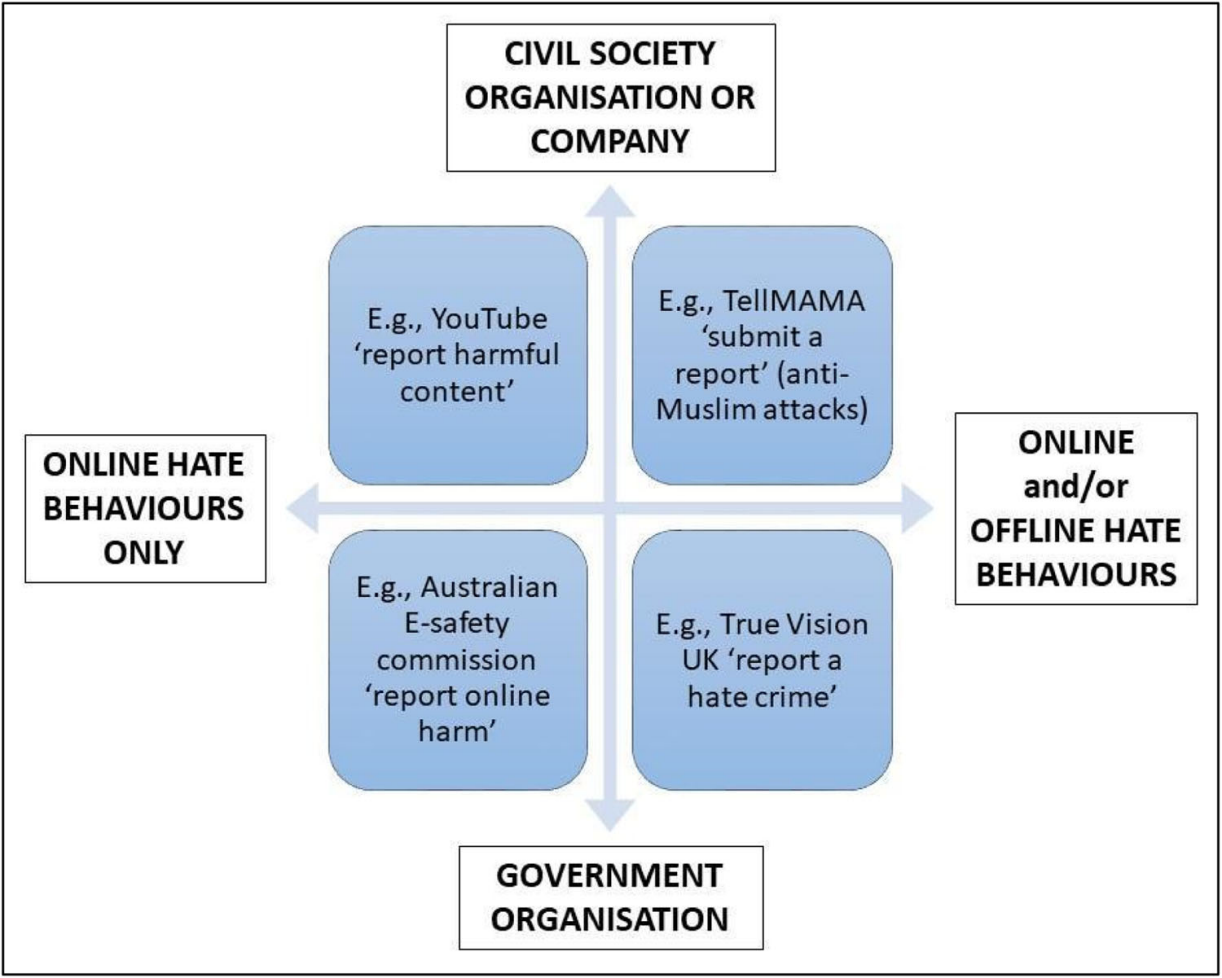
Visual representation of the four types of incidents reporting tools.

Figure 5 provides a visual representation of the four types with one example per type.

Private entities controlling IRTs can be either non‐profit (e.g., civil society organisations) or for profit (e.g., tech companies like Meta, Google, LinkedIn, Microsoft, among others) organisations. Most IRTs focusing exclusively on online hate behaviours are initiatives of tech companies. Examples of the platforms that provide the opportunity to report a variety of hate behaviours are YouTube, WordPress, WhatsApp, Twitter, Tumblr, Pinterest, Instagram, Facebook, Discord, Amazon, Airbnb (among others). Each platform has specific policies, terminologies and types of content that are not allowed. For example, YouTube has policies on hate speech, violent or graphic content, violent extremist or criminal organisations, harassment and cyberbullying. Individual users can report different types of content for violating one or more of these guidelines. In YouTube it is possible to report a video, a playlist, a thumbnail, a link, a comment, a live chat message, a channel or an ad (YouTube, n.d.). Additionally, all these platforms have automated algorithms – informed by human coders – to scan and identify content that potentially violates the policies. Although tech companies publish reports where they present the percentage of hateful content that they delete from each platform in a given period of time, there is no independent and transparent process to verify in detail how the policies are implemented and potential pitfalls. To our knowledge, tech companies do not publish datasets containing the content removed for violating guidelines regulating hate behaviours.

Most civil society organisations managing IRTs allow third parties (either victims or witnesses) to report a variety of hate behaviours, including criminal and noncriminal acts taking place both online and offline. Some civil society organisations collect incidents reports from one community. Examples are TellMAMA (collecting reports of anti‐Muslim hate in the UK; Feldman & Littler, 2014), B'nai Brith of Canada (collecting antisemitic incidents in Canada; B'nai Brith of Canada, 2021), Call it out (collecting reports of hatred against First Nations people in Australia) (Call It Out, n.d.). Other civil society organisations collect incident reports from multiple communities. Examples are Plataforma Khetane (collecting racist, xenophobic, antisemitic, Islamphobic, LGBTIfobic and anti‐Roma hate speech in Spain; Plataforma Khetane, n.d.), Stop Hate Alberta (collecting hate incidents reports based based on race, national or ethnic origin, language, colour, religion, sex, age, mental or physical disability, sexual orientation, or any other similar factor; Alberta Hate Crime Committee, 2021). Each organisation has different fields and categories of relevant hate behaviours, different procedures to verify the incident, and different approaches to the publication of data. For example, the Executive Council of Australian Jewry publishes an annual report that contains a brief narrative and deidentified description of all antisemitic incidents reports received during the year. This allows researchers to use the data for advancing the understanding of hate crime in the Australian context (Vergani et al., 2022).

Some IRTs controlled by government organisations focus on online incidents only. For example, the Australian e‐Safety commission has a web page that allows users to report a range of online harms, including cyberbullying of children, adult cyber abuse, image‐based abuse (sharing, or threatening to share, intimate images without the consent of the person shown) and illegal and restricted content (Office for Democratic Institutions and Human Rights, n.d.). The commission uses the report to investigate potential infringements of online regulations, as well as for research purposes. Many of the IRTs controlled by government organisations allow the reporting of both criminal and noncriminal acts taking place online and offline. An example is True Vision UK (True Vision, n.d.), which is a reporting platform owned by the National Police Chiefs' Council that allows to report a wide range of hateful behaviours demonstrating prejudice or hostility towards a person's disability, race or ethnicity, religion or belief, sexual orientation, transgender identity.

Our ability to fully assess and map IRTs is limited because of the same problem that we discussed in relation to official law enforcement data: oftentimes, civil society and government organisations controlling IRTs do not disclose the guidelines, training of frontline workers and methodological processes that they use to categorise and collect the data. For the majority of the IRTs that we identified, we were unable to retrieve a document (e.g., a report, a book or a journal article) discussing transparently the methodological approach underpinning the IRT. After completing our screening, we included only 21 IRTs for which we could assess their methodology, 18 in the grey literature, and 3 in the academic literature. Most IRTs (*N* = 16) that we included capture both criminal and noncriminal hate behaviours. Two capture hate incidents only, two hate crime and one hate speech only. In terms of geographical scope the next table illustrates the name and country of origin of our original IRTs (Table 18).

**Table 18:** Documents containing the incidents reporting tools included in this study by country context.

Document	Country
B'nai Brith of Canada (2021)	Canada
Alberta Hate Crime Committee (2021)	Canada
Asian Australian Alliance et al. (2020)	Australia
Nathan (2017)	Australia
Iner et al. (2017)	Australia
SOS Racismo (2015)	Spain
Michael (2021)	Ireland
TENI (2014)	Ireland
MacDonald et al. (2017)	UK
Feldman and Littler (2014)	UK
RIAS Berlin (2016)	Germany
RIAS Berlin (2017)	Germany
Benček and Strasheim (2016)	Germany
RIAS Berlin (2018)	Germany
Bundesverband RIAS (2020)	Germany
Stop AAPI Hate (2020)	USA
Nicolosi et al. (2020)	USA
Miller & Werner‐Winslow (2016)	USA
ADL (2018b)	USA
ADL (2018a)	USA
NCAVP (2017)	USA

We looked at the protected characteristics/target groups covered by our IRTs, and we found that 8 looked specifically at antisemitism, 3 at Islamophobia, 1 at anti‐Black, 2 at anti‐Asian, 1 at anti‐women incidents. Other protected characteristics covered by our included IRTs were race (*N* = 5), sexual orientation (*N* = 5), migration status (*N* = 4), gender identity (*N* = 4), ethnicity (*N* = 3), disability (*N* = 2), gender (*N* = 2), religion (*N* = 2), ideology (*N* = 1).

The following table reports the questions that appear at least once in the full database of the over 90 IRTs that we identified in our initial search, which we divided in three categories: information about the incident, about the victim, about the offender (Tables 19 and 20).

**Table 19:** Questions that are present in at least one of the analysed IRTs.

Incident	Victim	Offender
Time/date (ADL, 2018b; Albera Hate Crime Committee, 2021)	Age (Iner et al., 2017; Michael, 2021)	Age (Feldman & Littler, 2014; Michael, 2021)
Location/digital platform (website url) (ADL, 2018a, 2018b)	Gender/What pronouns do you prefer? (Asian Australian Alliance et al., 2020; Michael, 2021)	Gender (Feldman & Littler, 2014; Michael, 2021)
Incident description (ADL, 2018a; Asian Australian Aliance et al., 2020)	Protected characteristic (e.g., race, ethnicity, sexual orientation, disability) (NCAVP, 2017; RIAS Berlin, 2017)	Number of offenders (Michael, 2021)
Incident type (categories) (ADL, 2018a, Asian Australian Aliance et al., 2020)	Person/property (RIAS Berlin, 2016)	Affiliation/group of offenders (ADL, 2018b)
Online/offline (Iner et al., 2017; RIAS Berlin, 2017)	Relationship with offender (Iner et al., 2017; Michael, 2021)	Drug/alcohol usage (Michael, 2021)
Motivation type (e.g., antisemitic, homophobic) (Alberta Hate Crime Committee, 2021; Miller & Werner‐Winslow, 2016)	Physical injuries (Michael, 2021)	
Reported to police or other organisation (TENI, 2014)		

*Note*: Each cell also includes references for up to two example IRTs where the respective question is featured.

Abbreviation: IRT, Incident reporting tool.

**Table 20:** Summary of the protected characteristics covered by the IRTs identified in this review.

Protected characteristic	Number of IRTs covering it	Document
Antisemitism	8	B'nai Brith of Canada (2021); ADL (2018a); Bundesverband RIAS (2020); Miller and Werner‐Winslow (2016); Nathan (2017); RIAS Berlin (2016); RIAS Berlin (2017); RIAS Berlin (2018)
Race	5	Macdonald et al. (2017); Michael (2021); Nicolosi et al. (2020); SOS Racismo (2015); Alberta Hate Crime Committee (2021)
Sexual orientation	5	Macdonald et al. (2017); Miller and Werner‐Winslow (2016); NCAVP (2017); Nicolosi et al. (2020); Alberta Hate Crime Committee (2021)
Nationality/migration status	4	Benček and Strasheim (2016); Miller and Werner‐Winslow (2016); Nicolosi et al. (2020); Alberta Hate Crime Committee (2021)
Gender identity	4	Miller and Werner‐Winslow (2016); NCAVP (2017); Nicolosi et al. (2020); TENI (2014)
Islamophobia	3	Feldman and Littler (2014); Iner et al. (2017); Miller and Werner‐Winslow (2016)
Ethnicity	3	Michael (2021); Nicolosi et al. (2020); Alberta Hate Crime Committee (2021)
Religion	2	Nicolosi et al. (2020); Alberta Hate Crime Committee (2021)
Gender	2	Nicolosi et al., 2020; Alberta Hate Crime Committee, 2021)
Disability	2	Macdonald et al. (2017); Nicolosi et al. (2020)
Anti‐Asian hate	2	Asian Australian Alliance et al. (2020); Stop AAPI Hate (2020)
Anti‐Black hate	1	Miller and Werner‐Winslow (2016)
Anti‐women	1	Miller and Werner‐Winslow (2016)
Ideology	1	Miller and Werner‐Winslow (2016)

Abbreviation: IRT, Incident reporting tool.

###### Databases

Databases contain descriptors of incidents compiled by researchers using a variety of open and classified sources. They contain a range of variables about each incident, the victims and the offenders. We identified 26 databases that met our inclusion criteria, of which 19 used open sources and 7 used case records compiled by law enforcement agencies. Open source databases contain information about hate behaviours (hate crime, hate speech and hate incidents) collected from newspaper articles and other media reporting, sentences and other publicly available legal documents, reports by government and nongovernment organisations. Usually, researchers collect and code the information to create a database that can be used to perform statistical and qualitative analyses. Eleven of our included open source databases are found in the academic literature, and eight in the grey literature. Eleven documents measure exclusively hate crimes, six both criminal and noncriminal hate behaviours, and two hate speech.

An example of an open source database is found in Gruenewald (2012), where the author studied similarities and differences in anti‐LGBT homicides and average homicides in the United States between 1990 and 2008. The study identified all known anti‐LGBT homicides occurring in the United States between 1990 and 2008 from open‐sources. Homicides were identified by systematically searching sources, such as crime chronologies located in existing advocacy group reports (e.g., Human Rights Campaign, National Gay & Lesbian Task Force, Southern Poverty Law Centre, National Centre for Anti‐Violence Programmes [NCAVPs]), as well as systematic print news media searches using the LexisNexis search engine. Some of the key information on anti‐LGBT homicide events was gathered during the homicide identification process of each event from open‐sources. To supplement this information, an open‐search protocol was developed and used to search for additional information on each homicide in reports by watch‐group organisations, advocacy groups, major newspapers and search engines.

Case records contain information about hate behaviours (hate crime, hate speech and hate incidents) collected from police files, court records and other official data that is not publicly accessible. Researchers are usually given access to this data for research purposes, and publish only aggregate analyses that do not allow to identify the individual cases. Our included case records (*N* = 7) are all found in the academic literature. Five measure hate crime, and two measure both criminal and noncriminal hate behaviours.

An example of a case records approach is Krueger and Pischke (1997), where the author used police files and reports on more than 6500 suspects and offenders of criminal offences against foreigners in Germany between 1991 and 1993, along with court records on 154 perpetrators of criminal offences against foreigners. This rich data allowed him to code many personal characteristics of offenders, which are often missing from other data sources, alongside information about the incident type and the victim characteristics.

Table 21 reports the documents containing the databases and the country context that the databases capture. As the documents containing the German databases are in English language, only three documents are in Spanish and three in Italian language.

**Table 21:** Documents containing the databases included in this study by country context.

Document	Country context
Janoff (2005)	Canada
Provost‐Yombo et al. (2020)	Canada
Plataforma Ciudadana contra la Islamofobia (2017)	Spain
Giménez‐Salinas Framis et al. (2018)	Spain
Durán González and Pardo García (2008)	Spain
Lunaria (2020b)	Italy
Osservatorio Antisemitismo (2020)	Italy
Osservatorio Antisemitismo (2019)	Italy
Kielinger and Paterson (2007)	UK
Willems (1995)	Germany
Braun and Koopmans (2008)	Germany
Krueger and Pischke (1997)	Germany
Voigtländer and Voth (2012)	Germany
Waters and Yacka‐Bible (2017)	USA
Sank Davis (2019)	USA
Garafolo and Martin (1993)	USA
Gruenewald (2009)	USA
Smångs (2016)	USA
Gruenewald (2012)	USA
Howell et al. (2018)	USA
Jensen et al. (2021)	USA
Aguirre and Messineo (1997)	USA
Intersections of Sexual Violence: The Rape of Black Women by White Men (2013)	USA
D'Alessio et al. (2002)	USA
Jacobson and Royer (2011)	USA
Lee (2022)	USA

The protected characteristics/target groups covered by the databases are race (*N* = 14) – with a few databases focusing exclusively on Blacks (*N* = 2) – migration status (*N* = 14), sexual orientation (*N* = 10), religion (*N* = 7), ethnicity (*N* = 5), disability (*N* = 4), Antisemitism (*N* = 4), gender identity (*N* = 2), age (*N* = 2), Islamophobia (*N* = 1), ideology (*N* = 1), language (*N* = 1), sex (*N* = 1), and aporophobia (*N* = 1). Table 22 reports the variables included to describe the incident, the victim and the offenders.

**Table 22:** Variables that appear at least once to describe incidents, victims and offenders in the included databases.

Incident	Victim	Offender
Time/date	Age	Age
Location/digital platform	Gender	Gender
Type	Protected characteristics	Race/religion/ethnicity
Use of weapon	Person vs. property	Number of offenders
Public vs. private	Relationship with offender	Drug/alcohol usage
Online vs. offline	Employment	Education
Motivation type	Marital status	Employment
Planned vs. spontaneous	Income	Criminal record
Data source	Injuries	Group affiliation
Drug/alcohol involved		Family structure
Police response		Marital status

###### Quantitative survey questionnaires

Quantitative survey questionnaires are one or more questions used in survey research to measure the prevalence of a hate behaviour in a population that is relevant to the review. We included 53 quantitative survey questionnaires, 18 from the academic literature, and 35 from the grey literature. Broadly, we identified three types of quantitative survey questionnaires: official victimisation surveys, academic surveys, and other surveys run by a range of nongovernment and government organisations and departments.

Official victimisation surveys are tools that governments use to know about crime in the community. Usually they use large representative samples of a country's population, and they are therefore able to estimate how much crime there is in a certain community. By looking at the difference between the number of crimes that are reported to the police and the number of crimes from victimisation surveys, it is possible to estimate the size of under reporting of certain crimes – including hate crimes. In some countries, victimisation surveys collect data about the perceived motivation of the crime, that is, whether it is perceived to be motivated by bias or prejudice. For example, in the USA the Department of Justice's Bureau of Justice Statistics conducts a regular National Crime Victimisation Survey (NCVS), which includes hate crime data. Langton et al. (2021) developed and tested improvements to the NCVS survey questions, making the language clearer and more concise via qualitative interviews and a quantitative survey. Other countries have questions about prejudice motivation in official victimisation surveys, including Canada (in its General Social Survey – Canadians' Safety) and France (via its crime victimisation survey). In France, in 2022, a new questionnaire on the Experience and Feeling of Safety (VRS) has improved the collection of data on discriminatory phenomena.

Some countries have conducted specific reports about hate crime victimisations, such as, for example, Germany's Federal Criminal Police Office, which published a report on a country‐wide hate crime victimisation survey conducted between 2012 and 2017 (Birkel et al., 2018). Following the Christchurch terrorist attack, in the 2019 New Zealand Crime and Victims Survey, respondents were asked if they thought that incidents they had experienced were motivated by discrimination – that is, motivated by the offender's attitude towards the victim's race, sex, gender identity, sexual orientation, age, religion or disability. In 2021, Spain's Ministry of Interior published a hate crime victimisation survey report, which was carried out between 18 December 2020 and 31 March 2021. Some federal countries have state‐based data collection systems. In the UK, for example, experiences of hate crime are measured by the Crime Survey for England and Wales (CSEW), and by the Safe Community Survey in Northern Ireland. In Germany, the Criminal Police Offices of the federal states of Lower Saxony and Schleswig‐Holstein, respectively, conducted two regional‐level victimisation surveys on hate crimes. We found no official victimisation surveys containing country level hate crime data in Ireland, Italy and Australia.

Academic surveys can be found both in the academic and grey literature. For example, survey questionnaires are integral part of large research projects such as the Sussex Hate Crime Project (Paterson et al., 2018) and the Leicester Hate Crime Project (Chakraborti et al., 2014), and appear in the respective reports. Although the most comprehensive description of the methodology used appears in grey literature documents (i.e., research reports), they underpin a number of academic publications. Surveys are also used to measure online hate behaviours, as in Ortuno (2017), which conducted a survey to assess the prevalence of different kinds of online hate speech behaviours among 1502 Spanish Internet users. Herek et al. (1999) is an important survey published in an academic journal: the survey was designed to map criminal victimisation experiences with 2259 Sacramento‐area lesbians, gay men and bisexuals including psychological impacts such as depression, anger, anxiety, and post‐traumatic stress.

Other surveys run by a range of nongovernment and government organisations and departments often look at specific manifestations of hate in certain local contexts. An example are the numerous surveys administered by the European Union Fundamental Rights Agency (FRA), such as the EU‐wide survey on migrants and minorities (EU‐MIDIS II) that includes questions about hate crime victimisation (European Union Agency for Fundamental Rights, 2018b, 2019, 2020a). Non‐government and community organisations often run survey and design their own instruments. For example, the ADL conducts regularly victimisation surveys Jewish Americans to explore their experiences with antisemitism both online and offline (Anti‐Defamation League, 2020b).

Of the 53 original quantitative survey questionnaire tools that we collected, 23 collect data about both criminal and noncriminal behaviours, 15 about hate crime, 14 about hate speech, and 1 about hate incidents only. The largest shares of quantitative survey questionnaires are implemented in the USA context (*N* = 16) and in the UK context (*N* = 13). The remaining are implemented in Germany (*N* = 5), Ireland (*N* = 5), New Zealand (*N* = 5), Spain (*N* = 4), Italy (*N* = 3), France (*N* = 4), Canada (*N* = 4), Australia (*N* = 1). The vast majority of the survey questionnaires are contained in English language documents (87%, *N* = 46), three in Spanish, two in French, one in Italian and one in German language documents. The protected characteristics/target groups covered by the quantitative survey questionnaires are race (*N* = 16), sexual orientation (*N* = 12), gender identity (*N* = 9), religion (*N* = 5), disability (*N* = 5), sex (*N* = 3), nationality (*N* = 5), ethnicity (*N* = 4), social status (*N* = 3), ideology (*N* = 2), age (*N* = 3), language (*N* = 1), and other minority identity (i.e., Roma *N* = 1, Muslim *N* = 1; Jew *N* = 5). Table 23 provides the full list of documents containing the survey questionnaires included in this review by country.

**Table 23:** Documents containing the survey questionnaires included in this study by country context.

Documents	Country
Office of the eSafety Commissioner et al. (2019)	UK, Australia and New Zealand
European Commission (2017)	Germany, France, UK, Ireland, Italy, Spain, New Zealand
Pacheco and Melhuish (2019)	New Zealand
Coggan et al. (2003)	New Zealand
Netsafe (2021)	New Zealand
Ortuno (2017)	Canada, Spain
López Gutiérrez et al. (2021)	Spain
Gil‐Borrelli et al. (2020)	Spain
European Union Agency for Fundamental Rights (2019)	Germany, France, UK, Italy
Centro Risorse LGBTI (2020)	Italy
Jarman and Tennant (2003)	Ireland
Coughlan (2006)	Ireland
The Irish Immigrant Support Centre (2012)	Ireland
Kennedy (2013)	Ireland
Keipi et al. (2017)	USA, Germany, UK
Jansson (2006)	UK
Staetsky and Boyd (2015)	UK
Hubbard (2020)	UK
Broadstock (2013)	UK
Dick (2009)	UK
All Party Parliamentary Group on British Muslims (2018)	UK
Chakraborti et al. (2014)	UK
Paterson et al. (2018)	UK
Frazer (2005)	UK
Williams and Tregidga (2014)	UK
Ofcom (2023)	UK
Ofcom (2022)	UK
Soullez (2017)	France
Centre Hubertine Auclert (2018)	France
Álvarez‐Benjumea and Winter (2020)	Germany
Birkel et al. (2018)	Germany
Herek et al. (1999)	USA
Motley (2021)	USA
Herek (1993)	USA
Harriman et al. (2020)	USA
Glaser et al. (2002)	USA
Barnidge et al. (2019)	USA
Wachs et al. (2021)	USA
Simich and Kang‐Brown (2018)	USA
ADL (2022)	USA
ADL (2020b)	USA
Yellow Horse et al. (2021)	USA
Jones et al. (2019)	USA
Chiang (2021)	USA
Von Schulthess (1992)	USA
Statistics Canada (2019)	Canada
Baker (2017)	Canada
Commission des Droits de la Personne et des Droits de la Jeunesse (2019)	Canada
He et al. (2021)	Global
European Union Agency for Fundamental Rights (2018a, 2018b)	EU states
European Union Agency for Fundamental Rights (2020a)	EU states
Oakley (2006)	EU states
European Commission (2016)	EU states
Turner et al. (2009)	EU states
Langton et al. (2021)	USA
Assimakopoulos et al. (2017)	EU states

Table 24 reports the variables that are collected at least once in our quantitative survey questionnaires, which we divided in three categories: information about the incident, about the victim and about the offender.

**Table 24:** Variables that appear at least once to describe incidents, victims and offenders in the included surveys.

Incident	Victim	Offender
Time/date	Age	Age
Location/digital platform	Gender	Gender
Incident type	Protected characteristic	Number of offenders
Weapons use	Employment	Race
Motivation type	Relationship with offender	Drug/alcohol usage
Reported to police	Changes in behaviour	Stranger vs. known
Reported to others	Knowledge hate crime law	
Number of witnesses	Social media use and behaviour	
Description open ended	Income	
Link between online and offline events	Physical injuries	
	Psychological impact	

###### Manual quantitative text analysis tools

Manual quantitative text analysis tools are guidelines to analyse text with the aim to measure the frequency of a hate behaviour. As opposed to computer science automated or semi‐automated tools, the coding and processing of the data is completely manual, that is, conducted by one or more researchers. We included 14 manual quantitative text analysis tools, of which 9 in the academic and 5 in the grey literature. All of them aim to capture hate speech except for one (Bérubé & Campana, 2015), which analyses the discourses conveyed in the commission of acts of hate violence in Canada between 1977 and 2010. A typical example of manual quantitative text analysis tool focusing on hate speech is found in Hameleers et al. (2021), which presents a content analysis of fact‐checked statements in the USA (*N* = 894) to assess to what extent and how different forms of incivility and hate speech are present in different degrees of false information. Two coders assessed each statement and coded the presence of four categories of ‘incivility’: general hostility and negativity, negative or hateful sentiments targeted at political opponents (partisan attacks), attacks against mainstream media, and hateful speech targeted at minority groups (hate speech). The coded data was then used in quantitative analysis (e.g., logistic regression models) (Hameleers et al., 2021).

Table 25 reports the documents containing the manual quantitative text analysis tools and the country context where they were used. Five documents are in English, four in French, three in Italian and two in Spanish language.

**Table 25:** Documents containing the manual quantitative text analysis tools included in this study by country context.

Document	Country
Losada‐Díaz et al. (2021)	Spain
Paz et al. (2021)	Spain
Muyor Rodriguez and Segura Sanchez (2020)	Spain
Bartlett and Krasodomski‐Jones (2015)	France, UK, Italy
Amnesty International Italia (2020)	Italy
Belluati (2018)	Italy
Associazione 21 luglio (2013)	Italy
Janto‐Petnehazi (2018)	UK
Larchet (2018)	France
Baider (2019)	France
Hanzelka and Schmidt (2017)	Germany
Hameleers et al. (2021)	USA
ADL (2020a)	USA
Bérubé and Campana (2015)	Canada

The manual quantitative text analysis tools focus on a range of protected characteristics/target groups, specifically: religious and ethnic communities (being Jewish *N* = 1, Roma *N* = 2), sexual orientation (*N* = 8), migration status (*N* = 6), gender (*N* = 5), religion (*N* = 6), ethnicity (*N* = 6), race (*N* = 4), women (*N* = 3), disability (*N* = 3), sex (*N* = 2), ideology (*N* = 1), age (*N* = 1), being a detainee (*N* = 1). In addition to collecting data about the frequency and types of a hate behaviour, our tools collected other information, specifically: the source of the text, the outlet in which the original statement appeared, the topic of the statement, the type of verification, measures of uptake (number of likes, comments divided between positive and negative), and all the available biographic information about the authors (e.g., user name, image, gender, age, ethnicity), location and time of the posting.

###### Automated or semi‐automated text analysis tools

The automated or semi‐automated text analysis tools are instruments that automate all or part of the identification of hate speech found in text or other multimedia content using a model. They fall into two groups based on how the model is trained. They are either *supervised*, where the model is ‘taught’ to recognise the characteristic to be identified, or *unsupervised*, where the model aims to identify patterns or groups in the data without prior training. The supervised learning methods can be divided into two groups, those based on Artificial Neural Networks (ANNs), and all other methods. Overall, we included 21 documents containing 68 individual studies (i.e., instances) where different models were tested. Table 26 provides the full list of documents containing the included automated or semi‐automated text analysis tools by language used.

**Table 26:** Variables that appear at least once to describe incidents, victims and offenders in the included automated and semi‐automated text analysis tools.

Document	Language analysed
Charitidis et al. (2020)	English, Italian, German, Spanish, French, Greek languages
Burnap and Williams (2016)	English language
Burnap and Williams (2015)	English language
Alzamzami and El Saddik (2021)	English language
Ayo et al. (2021)	English language
Lee (2021)	English language
Pamungkas et al. (2020)	English and Spanish languages
ElSherief et al. (2018)	English language
Sadiq et al. (2021)	English language
Pereira‐Kohatsu et al. (2019)	Spanish language
Lindenmayr et al. (2021)	German language
Mohapatra et al. (2021)	English language
Miller (2017)	English language
Khatua and Nejdl (2022)	English language
Siapera et al. (2018)	English language
Pérez‐Landa et al. (2021)	English language
Mollas et al. (2022)	English language
Kapil and Ekbal (2020)	English language
Di Nicola et al. (2020)	English, Italian and French languages
Agarwal and Chowdary (2021)	English language
Chiril (2021)	English and French languages

*Artificial Neural Network‐based methods* are the most popular methods currently for classifying hate speech from text, with 76% (16/21) of papers evaluating an ANN‐based model and 58% (40/68) instances overall reported as being of this type. These models exploit the ability of the classic ANN to effectively create a mapping from high dimensional textual input to a classification of whether or not the text string represents hate speech via a number of ‘Hidden’ Layers that effectively create connections between the words in text strings to be classified. These classical models are extended by Convolutional Neural Networks (CNNs) that effectively increase the complexity of the learning model by increasing the connections between inputs by increasing the number of layers in the model – giving rise to the concept of ‘Deep Learning’. Long Short‐Term Memory and Gated Recurrent CNNs further improve classification by enabling the model to selectively ‘remember’ and ‘forget’ rules when it improves the accuracy of the model. Bidirectional Encoder Representations from Transformers (BERT) was used by 28% (6/21) of the included documents to either optimise the representation of the text to be classified in vector space for classification by another CNN, or to perform the actual classification in 8 instances.

*Other supervised learning methods* accounted for 36% (25/68) of the instances reported. These included decision tree‐based methods 12% (8/68 instances), in which the model encapsulates a set of sequential (branching) decisions based on attributes that best distinguish between hate/non‐hate, and so forth. Gradient Boosted Decision Trees and Random Forests both improve on the basic tree. Other supervised learning methods included Naïve Bayes' (4/68 instances) which performs a probabilistic classification through comparison to a labelled (‘hate/not‐hate’ corpus of examples; Support Vector Machines (7/68 instances) in which classification is performed by dividing the classes (‘hate/non‐hate’) in high dimensional space; and regression‐based models.

*Unsupervised learning* (where the model is not ‘taught’ to recognise a target, but instead clusters the data according to a similarity measure) was reported in 4% (3/68) of the instances. The two methods reported, k‐Nearest Neighbours (one instance) is a method of clustering data into groups having similar properties. Latent Dirichlet Allocation (2 instances) is used for topic modelling, that is, to find the most commonly occurring topics in a corpus of documents by probabilistically associating each document with a given topic. In this approach, a document (tweet, e.g.) would be probabilistically associated with a specified hate‐related topic, to make the classification of hate or not (Table 27).

**Table 27:** Proportion (number of instances) of principal methods implemented in papers reviewed.

Artificial neural network‐based (40/68)	Other supervised learning (25/68)	Unsupervised Learning (3/68)
Artificial Neural Network (ANN) (8) Bidirectional Encoder Representations from Transformers BERT (7) Convolutional Neural Network (CNN) (10) Long Short‐Term Memory (LSTM) (11) Recurrent Neural Network (RNN) (4)	Decision Tree (DT) Gradient Boosted Decision Tree (GBT) Naïve Bayes' (Bayes) (4) Quadratic Discriminant Analysis (QDA) Random Forests (RF) (6) Regression (4) Rule‐Based Support Vector Machine (SVM) (7)	k‐Nearest Neighbours, kNN Latent Dirichlet Allocation (LDA) (2)

###### Qualitative interview schedules

Qualitative interview schedules are lists of interview questions and/or protocols aiming to understand the different manifestations of a hate behaviour in the eye of a victim or a witness: 15 interview schedules met our inclusion criteria, and they were all found in the academic literature. Seven of them focus on behaviours across criminal and noncriminal thresholds, and six on hate crime. Only one focuses on hate speech, and one on hate incidents. Qualitative interview schedules can be structured or unstructured. Sandhu (2019) used semi‐structured interview schedules to interview seven Sikh Americans about racial and religious discrimination. The author reports a full list of questions asked during the interview, including questions aiming to explore the experiences and manifestations of hate such as ‘Research has shown that there was a rise in Islamophobia/discrimination against individuals that “appear Muslim” since 9/11. Can you share what your experiences have been like since 9/11?’ (Sandhu, 2019, p. 242).

Bell and Perry (2015) provide an example of an unstructured approach to conduct focus groups – which they argue have the advantage of their informal nature that encourages free participation and a naturalistic research setting. The study included a sample of 15 participants who identified as being lesbian, gay, bisexual or pansexual. The interview focused on topics such as anti‐LGB crimes and their effects on the community, and was conducted with a nondirective approach ‘to encourage participants to speak freely about issues of importance to them’ (Bell & Perry, 2015, p. 105). Table 28 reports the documents containing the qualitative interviews schedules and the country context where they were implemented. Fourteen documents are in English language and one in French language.

**Table 28:** Documents containing the qualitative interview schedules included in this study by country context.

Citation	Country context
Block et al. (2021)	Australia
Rawlings (2019)	Australia
Assimakopoulos et al. (2017)	UK, Italy, Spain
Langarita Adiego et al. (2019)	Spain
Baghdadi (2014)	UK
Flax (2018)	UK
Zaman (2009)	USA
Sandhu (2019)	USA
Ndiaye (2020)	USA
Ghafur (2021)	USA
Zhang et al. (2019)	USA
Herek et al. (2002)	USA
Agrawal et al. (2019)	USA
Bell and Perry (2015)	Canada
Mercier‐Dalphond and Helly (2021)	Canada

The qualitative interview schedules covered a small range of protected characteristics, specifically: religion (being Muslim *N* = 6, Jewish *N* = 1, Sikh *N* = 1), sexual orientation (*N* = 3), migration status (*N* = 1), gender identity (*N* = 2). To describe the incident, in all cases the interviewees were asked to describe freely the experiences that they regard as a hate behaviour. In case of interviews with children, coders were trained to identify hate behaviours even when the child was not aware that they were. In interviews with adults, the interviewee is asked to explain why they believe that the incident was hate motivated. Data about the victims were collected to screen interview participants (e.g., if the interviewee was a refugee or their religious affiliation). Additionally, all interviews report that they collected age and gender of the interviewee. Five articles collected additional variables such as education, profession and income. Only one article collected information about the offender, specifically: number of offenders, offenders' gender, and relationship with the victim.

###### Qualitative text analysis tools

Qualitative text analysis tools are guidelines to analyse text with the aim to understand the different manifestations of a hate behaviour: 18 qualitative text analysis tools met our inclusion criteria: 14 of them were found in the academic literature, and 4 in the grey literature. The majority of them focus on hate speech (*N* = 17), with the exception of Bartle (2000) that focuses on hate crime and analyses congress hearings text to understand the experiences of victims of anti‐lesbian hate crime in the USA. An example of a qualitative text analysis tool is Brindle (2016), which uses discourse and linguistic analysis to analyse text produced by the English Defence League (EDL). The analysis identified a list of terms that were most used in the group's online pages (e.g., Islam, radical, militant) and then conducted a qualitative discourse analysis to understand the context and meanings associated with the terms. The analysis uncovered different manifestations of Islamophobia. Table 29 reports the documents containing the qualitative text analysis tools and the country context where they were used. One document is in Italian language, 5 in French language, and 12 documents are in English language.

**Table 29:** Documents containing the qualitative text analysis tools included in this study by country context.

Document	Country context
Richardson‐Self (2019)	Australia
All Together Now (2019)	Australia
All Together Now (2021)	Australia
Assimakopoulos et al. (2017)	UK, Italy, Spain
Lunaria (2019)	Italy, Spain
Rodríguez‐Darias and Aguilera‐Ávila (2018)	Spain
Ben‐David and Matamoros‐Fernández (2016)	Spain
Bernardez‐Rodal et al. (2022)	Spain
Brindle (2016)	UK
Allington (2018)	UK
Goodman and Rowe (2014)	UK
Asquith (2010)	UK
Napieralski (2018)	France
Seoane et al. (2020)	France
Vernet and Määttä (2021)	France
Bibié and Goudet (2018)	France
Napieralski (2017)	France
Bartle (2000)	Canada

The qualitative text analysis tools focus on a small range of protected characteristics/target groups, specifically: religious and ethnic communities (being Muslim *N* = 2, Jewish *N* = 2, Roma *N* = 2, Black *N* = 1), sexual orientation (*N* = 5), migration status (*N* = 5), ethnicity (*N* = 3), race (*N* = 3), religion (*N* = 2), gender (*N* = 2), gender identity (*N* = 1), women *N* = 1, and feminists (*N* = 1). In addition to collecting data about the different manifestations of a hate behaviour, the included qualitative text analysis tools collected other information, specifically: publicly available biographical information of the author of the text (e.g., gender, age, political affiliation, ethnicity), the links and resources cited in the text, the uptake of the text (measured by looking at reactions, shares, followers of the page/group where it was shared).

##### RQ2. How valid and reliable are these measures?

Overall, our research shows that the data about the validity and reliability of the measurement tools is generally not reported. The only corpus of study that allowed us to collect and analyse comparatively the validity of the included measurement tools is computer science. For all other measurement tools, the lack of data made it impossible for us to conduct a systematic and comparative reliability and validity assessment.

###### Law enforcement data sources

No official data source report that we included in our data set provides estimates of under reporting (i.e., our validity proxy). Under reporting figures are reported in other official documents: for example, Sandholtz et al. (2013) compared data from UCR and hate crime victimisation surveys, and found that only about one‐third of hate crimes are reported to the police. However, the underreporting estimates are not reported consistently in official hate crimes reports – with few exceptions like Canada's General Social Survey. In USA (Federal Bureau of Investigation, 1998) and Canada (Statistics Canada, 2022), the number of police agencies that submitted hate crime data is reported (i.e., our reliability proxy). This is an important indication, because it can lead to a significant public discussion about the reliability of the data reported by police. For example, when the FBI transitioned officially from the UCR to the NIBRS system, there was a substantial drop in participation from police agencies (from 15,138 in 2020 to 11,883 in 2021), which invalidates comparisons with previous – and possibly future – years. In other reports (e.g., Bundesministerium des Innern, für Bau und Heimat, 2018) the number of submitting police agencies is not reported. We did not include in the analysis countries that do not have a unitary country‐level reporting system (UK, Australia). The lack of standard reporting of reliability and validity data, as well as the different data collection systems and regulatory frameworks across different countries, impedes a cross‐country comparison.

###### IRTs

Only 8 of 21 IRTs that we included in our analysis provide a narrative description of the data verification process. For example, Anti‐Defamation League (2018a) writes:

All Incidents are assessed by ADL staff for credibility. Wherever possible, ADL staff obtain independent verification of incidents. Where verification is unavailable, incidents may still be included if ADL staff consider the reports to be credible using their best professional judgment.

Similarly, reporting practices are discussed in a narrative way in 8 of 21 documents. For example, Transgender Equality Network Ireland (TENI; Transgender Equality Network Ireland, 2014) writes:

Despite a communications strategy and extensive outreach, TENI received less reports than initially expected. This is likely due to a variety of factors. As in many countries in Europe, there is not a culture of reporting transphobic or homophobic crimes in Ireland. While this relates specifically to the police, there are ramifications for this project as trans people will often choose not to report the incident and instead seek support from friends or family.

Only 5 reports (Bundesverband RIAS, 2020; Iner et al., 2017; Nathan, 2017; NCAVP, 2017; TENI, 2014) discuss some aspects related to validity and reliability. However, the standards of discussion vary greatly, and no comparison across different tools is possible.

###### Case records and open source databases

Only 7 of 26 documents including case records and open source databases report a narrative description of the bias present in the original data source (i.e., our validity proxy). For example, Willems (1995, p. 506) writes:

Using data collated by the police raises a number of questions in relation to the assessment of its quality and validity. Firstly, only the criminal and violent xenophobic acts registered with the police, i.e., reported, are included. The number of crimes that are not reported for various reasons remains an open question. It must be presumed that the actual number of xenophobic crimes and acts of violence is higher than the figure obtainable from police statistics. Secondly, the criteria according to which criminal and violent acts are categorised by the police as xenophobic are by no means unequivocal; the definition and categorisation can be different for each individual county borough and state (Bunderland). In some cases all crimes in which foreigners, refugees, or even other victims (gays, handicapped) are harmed are included in the statistics, even if it is not clear whether rightist, racist, or other xenophobic motives were actually the underlying cause.

Similarly, only 5 of 21 documents report a narrative description of bias in the data collection process (i.e., our reliability proxy). For example, Waters and Yacka‐Bible (2017) writes:

NCAVP knows that the number of homicides is likely higher as some homicides of LGBTQ and HIV‐affected people are not documented because of misidentification of victims' sexual orientation or gender identity in media and other reports.

Importantly, only two documents (Waters & Yacka‐Bible, 2017; Willems, 1995) report a narrative discussion of both reliability and validity.

###### Quantitative survey questionnaire

Validity is discussed in 13 of 51 documents containing original measurement tools. The precision of the discussion varies across the sample. Some documents reported discussions of sample bias. For example, European Union Agency for Fundamental Rights (2018b, p. 66) writes:

the survey aspired to national coverage of the target groups in each country, but in some cases this was not feasible. In multi‐stage sampling, areas with low densities of the target population were excluded because screening of the target population would not have been possible in an efficient manner. In most countries, areas with target population densities below a certain thresh old had to be excluded. These limitations were unavoidable due to the need for labour‐intensive screening of respondents in most countries. Weighting The survey results presented in this report are based on weighted data to reflect the selection probabilities of each household and individual based on the sampling design.

Other studies present a more standard validation process of the survey instrument, for example Simich and Kang‐Brown (2018, p. 22) write:

For the purpose of initial content validation, Vera interviewed three experts (New Jersey key informants in bias crime law enforcement and Latino and Muslim community leadership positions) who had reviewed the BCAT and Guidelines. We asked them to answer a general validation question, does the BCAT adequately capture a reasonable operational definition of hate crime? […] As a result of this development process and preliminary validation process, the BCAT is more victim‐centered than standard hate crime reporting tools.

Replicability of findings is discussed in general terms in 9 documents, but no document in our sample presented a test‐retest reliability study (i.e., our proxy of reliability). Given the lack of consistent metrics used in this literature, it is impossible to compare the validity and reliability of the included studies.

###### Automated or semi‐automated text analysis tools

In the computer science literature, although no document reported a discussion of the bias in the data sources (e.g., sample bias), coefficients of precision, recall and F1 (i.e., our validity proxies) are reported in 18 out of 20 documents. Precision is the proportion of instances identified as ‘is hate’ that are actually hate speech. Recall is the proportion of hate speech that was identified in the corpus. F1, the harmonic mean of precision and recall, is calculated as (2*(precision * recall)/(precision + recall)). Table 30 shows results for the best performing classifier in each paper. As well, results were not given for LDA (Miller, 2017) since it is unsupervised learning, nor for the multivariate regression (ElSherief et al., 2018), which only assessed the key factors in predictability but did not test the model on unsighted data.

**Table 30:** Precision, recall and F1 measures for best method reported.

Document	Classifiers evaluated. The actual classifier or method reported in bold.	Precision	Recall	F1
Charitidis et al. (2020)	ANN, CNN, LSTM (all methods as an ensemble)	0.8	0.82	0.81
Burnap and Williams (2016)	RF, SVM	0.79	0.59	0.68
Burnap and Williams (2015)	Bayes, RF, SVM (all methods as an ensemble)	0.89	0.69	0.78
Alzamzami and El Saddik (2021)	BERT, CNN, LSTM	0.85	0.89	0.87
Ayo et al. (2021)	Bayes, BERT, CNN, CNN + GRU, LSTM, RL, RNN, Rule‐Based‐Clustering	0.93	0.92	0.93
Lee (2021)	ANN, BERT, LSTM, RNN	0.94	0.92	0.93
Pamungkas et al. (2020)	ANN, BERT, LSTM, RNN + GRU	0.7	0.66	0.68
ElSherief et al. (2018)	RM	NA	NA	NA
Sadiq et al. (2021)	MLP, CNN + LSTM	0.9	0.9	0.9
Pereira‐Kohatsu et al. (2019)	ANN, LDA, LSTM + MLP, QDA, RF, RR, SVM	0.91	0.89	0.90
Lindenmayr et al. (2021)	CNN, Lexicon, SVM	NA	NA	NA
Mohapatra et al. (2021)	Bayes, RF, SVM	0.76	0.73	0.73
Miller (2017)	LDA	NA	NA	NA
Khatua and Nejdl (2022)	BERT, CNN BERT + CNN	0.79	0.76	0.76
Siapera et al. (2018)	ANN, LSTM	NA	NA	NA
Pérez‐Landa et al. (2021)	DT, kNN			0.76
Mollas et al. (2022)	ANN, Bayes, BERT, CNN, GBT, LSTM, RF, RL, SVM	0.77	0.78	0.77
Kapil and Ekbal (2020)	ANN, CNN, LSTM			0.89
di Nicola et al. (2020)	ML, not described	NA	NA	NA
Agarwal and Chowdary (2021)	CNN, LSTM, RL, RNN + LSTM + (ensemble of GBT, SVM, MLP, kNN), SVM	0.73	0.73	0.74
Chiril (2021)	BERT, CNN+Lexicon, LSTM, SVM	0.86	0.80	0.83

Note that in some cases the methods and results obtained by some researchers have been superseded by newer methods with improved results. For example, Burnap and Williams (2015, 2016) have obtained improved hate classification using a two‐stage fuzzy classifier (Liu et al., 2019a) having the best reported performance as (*p* = 0.88, *R* = 0.71 and *F* = 0.79) and a multi‐task fuzzy classifier identifying whether or not a text is hate speech, the type of hate, and the topic or context (Liu et al., 2019b) having an average *p* = 0.93 over four types of hate speech (religion, race, disability, sexual orientation). Looking at the predominant classifiers by year, it can be seen that the types of models used, and evolution of ML classifiers over time is reflective of the development of Statistical Learning/Artificial Intelligence as a whole. For example, the earliest models are Bayesian, decision tree‐based or Support Vector Machine, including ensembles of these. ANNs and the related LSTM models are present from 2018, with BERT and CNNs present from 2020, and still currently reflecting the state‐of‐the‐art. It should be noted that even some of the earlier classifiers reviewed produce satisfactory results (Burnap & Williams, 2015). There is a high degree of variability between results, even when similar methods are used, due to differences in datasets, training regime, protected characteristics and the prevalence of that characteristic in the data set to be classified. This makes it impossible to propose a single best method. Notwithstanding, a few features of these results can be observed. A key feature of the data is that the best performing classifiers, say those with an F1 score > 0.8 are all ANN or CNN‐based, with the exception of (Ayo et al., 2021), which used a fuzzy logic, rule‐based clustering method. As well as these ‘best’ classifiers, our results shows that the majority of the competitor classifiers, those the best is compared against, are also of various deep learning types. This does indicate the superiority, and hence prevalence, of these methods for hate speech classification at the current time. Inter rater agreement and/or strategies to reduce bias in labelling data (i.e., our reliability proxy) was addressed in 8/21 of documents. This included reporting the agreement between coders (Cohen/Fleiss' Kappa, e.g.) in 4 cases.

###### Manual quantitative text analysis tools

Validity (i.e., bias in the original data source) was discussed in only 1 of 10 included measurement tools. Specifically, Hanzelka and Schmidt (2017, p. 150) write:

a possible distortion has to be taken into account. In the space of social media and the internet generally, the instability of information is a great problem for the research. Users can delete their content or modify it over time, and this generates changes which cannot be registered. This problem is especially connected with the measurement of the real number of hateful comments. When a page administrator, some third party or users themselves delete these comments, there is no option for backtracking.

Inter rater reliability (i.e., our validity proxy) was discussed and reported in 4 out of 10 documents as the % value of the agreement between coders and in some cases the Krippendorff's alpha coefficient. For example, Paz et al. (2021, p. 3) simply write:

we reached 94.8% of intercoder reliability.

Hameleers et al. (2021, p. 8) write:

Even though most variables were coded with a sufficient reliability (Krippendorff's alpha.63, agreement 82%), all differences between coders were discussed until complete agreement was reached.

###### Qualitative interviews schedule

Issues related with the quality of the data collected (i.e., our validity proxy) are discussed in 8 of 14 documents containing qualitative interviews schedules. The key discussions relate to small sample size and inability to generalise the findings (Agrawal et al., 2019; Bell & Perry, 2015; Ghafur, 2021; Herek et al., 2002; Zaman, 2009; Zhang et al., 2019), sample bias due to recruitment strategy (Ghafur, 2021; Ndiaye, 2020; Sandhu, 2019; Zaman, 2009; Zhang et al., 2019), desirability and interviewer bias (Agrawal et al., 2019; Ghafur, 2021; Herek et al., 2002; Ndiaye, 2020; Zhang et al., 2019). Replicability (i.e., our reliability proxy) were not discussed in any of the documents included.

###### Qualitative text analysis tools

Only one of the documents containing a qualitative text analysis tool discussed potential bias in the data and data collection process (i.e., our proxy of validity). Assimakopoulos et al. (2017) write:

the qualitative analyses […] provided the discursive context, both in terms of the characteristics of the newspaper (e.g., tabloid or broadsheet, political orientation) and the interactional status of the comment (e.g., direct or tangential response to the article, response to another contributor). In addition, the reasons for the polarity categorisations of expressions as more or less negative or positive (or ambiguous) were stipulated by each group of analysts. In this way, what could be taken as subjective categorisations were given a degree of transparency. The shared analytical approach resulted in lists of expressions with their categorisations that permit cross‐country comparisons at a general level.

None of the documents discussed replicability of the procedures used (i.e., our reliability proxy).

## 6 DISCUSSION

### 6.1 Summary of main results

Our review has two objectives that are fundamentally connected: mapping (1) definitions and (2) measurement tools of hate crime, hate speech, hate incidents and surrogate terms. We provide as annex complete lists of the original definitions and measurement tools that met our inclusion criteria, for the use of researchers and policy makers worldwide. They are 423 definitions and 168 measurement tools in academic and grey literature, and 83 definitions in legislation documents. To support future research and policy work in this area, we included a synthetic assessment of the (1) the operationalisability of each definition and (2) the theoretical robustness and transparency of each measurement tool (see Section 6.3). The annexes providing the lists of definitions and measurement tools are the key outcome of this project, because they provide a useful toolbox for the next generation of policy and research in this area.

We mapped a fragmented terrain, where key definitional elements such as the protected characteristics of the victims, the types of behaviours (e.g., whether criminal vs. noncriminal acts), the targets of the behaviours (e.g., whether groups, individuals, properties, organisations) are named and conceptualised differently. Importantly, we found that 41% of the documents addressing a relevant hate motivated behaviour do not provide any definition. This is a concerning finding that raises questions about the overall quality of the research in this policy domain.

This review presented in Section Data Collection and Analysis, 5 a detailed mapping of the features of the relevant definitions and measurement tools. Our mapping revealed numerous significant trends, clusters and differences between and within definitions and measurement tools focusing on hate crime, hate speech and hate incidents. For example, definitions and measurement tools tend to focus more on ethnic and religious identities (e.g., racism, antisemitism, Islamophobia) compared to sexual, gender and disability‐related identities. This gap is greater in the definitions and measurement tools of hate speech than hate crime. Also, our analysis showed geographical patterns: hate crime definitions and measurement tools are more likely to originate from Anglophonic countries, especially the USA, but hate speech definitions and measurement tools are more likely to originate from continental Europe. In terms of disciplinary fragmentation, our social network analysis revealed that the collaboration and exchange of conceptual frameworks and methodological tools between social sciences and computer science is limited, with most definitions and measurement tools clustering along disciplinary lines. More detailed findings are presented in Section Data Collection and Analysis, 5.

### 6.2 Overall completeness and applicability of the evidence

The fragmentation of terminology in the fields of research and practice that we mapped is such that we may have inadvertently excluded some relevant definitions or measurement tools. In our search strategy, we searched for all the terms – like ‘hate’, ‘prejudice’ and ‘bias’ – that are central to our aims, as well as all the terms capturing neighbouring concepts – like ‘racism’, ‘homophobia’, ‘sexism’ – that had a higher chance of retrieving relevant results. However, we excluded search terms that, in isolation, would have retrieved large bodies of literature irrelevant to our aims: examples are ‘discrimination’, ‘harassment’, ‘sex crimes’, ‘domestic violence’. Documents including these terms were retrieved and screened only if they contained one or more of our hate‐related search terms (see our Protocol document for the full list of search terms used in our searches). Although the vast majority of documents using these keywords would have addressed topics outside the scope of our review, we acknowledge that we might have missed a few relevant items.

However, we are confident that – given the large number of original definitions and measurement tools retrieved using our search strategy, including in documents looking at discrimination, harassment and gender‐based violence – we have reached saturation of themes and types of definitions and measurement tools. Importantly, our search strategy did not exclusively rely on the keyword searches of academic databases: in searching for legislation and grey literature documents, we interviewed subject matter experts in each country contexts and we reviewed the references of many relevant reports and academic reviews. This additional search strategies allowed us to complement and overcome some of the limitations of the keywords search.

#### 6.2.1 Limitations in the assessment of definitions found in legal documents

We acknowledge that we are not able to assess the quality of definitions found in legislation documents using the methodologies of a systematic review. Legal definitions should be assessed using a different set of methods, including the analysis of sentences and case law (in common law jurisdictions), and potentially interviews with prosecutor, judges and other legal stakeholders to understand the practical pitfalls of how definitions are applied in court cases and within specific legal systems.

#### 6.2.2 Limitations in the assessment of measurement tools used by government and nongovernment organisations

A comprehensive and systematic comparative assessment of measurement tools used by government and nongovernment organisations (i.e., law enforcement data, incidents reporting tools used by government and nongovernment organisations) across country contexts is impracticable in the context of this work because the systematic review methods are not suited for conducting such assessment. As much of the information about the guidelines used by frontline workers to record hate behaviours, the training materials available to them, as well as other country‐specific issues (e.g., country‐specific legislative frameworks), a meaningful assessment would require a different design including interviews, content analysis, ethnographic methods.

#### 6.2.3 Limitations of social network analysis

It is important to acknowledge the limitations of the network map and analysis presented here. Out of the 145 grey literature documents containing original definitions, 120 (83%) did not have any referencing data on Semantic Scholar. Similarly out of the 50 grey literature documents containing original measurement tools, 36 (72%) did not have referencing data. This is in contrast to academic documents, which respectively only had 36 (19%) and 12 (15%) documents containing definitions and measurement tools missing its referencing data. This means that our map is biased towards a greater representation of academic literature. We believe that this does not weaken our interpretation about the disciplinary clusters that emerge from our analysis.

### 6.3 Quality of the evidence

In the context of this review, our assessment of the quality of the definitions and measurement tools does not aim to give a value judgement of their absolute worth. Rather, we conceptualise ‘quality’ as operationalisation for measurement (see Section Search Methods, 3). Specifically we assess: the extent to which a *definition* is capable of being operationalised into a measurement tool (objective 1); and the extent to which a *measurement tool* has solid theoretical foundations and uses transparent and replicable procedures (objective 2).

#### 6.3.1 How operationalisable are definitions?

As explained in Section Inclusion Criteria, 4 of this report, we created a composite score to assess the level of operationalisability of definitions along four categories. In our sample, 3% (*N* = 13) of definitions were coded low operationalisation level, 55% (*N* = 231) medium‐low operationalisation level, 34% (*N* = 144) medium‐high operationalisation level, and 8% (*N* = 35) high operationalisation level. The following table reports examples of definitions in each category (Table 31).

**Table 31:** Examples of definitions by operationalisability level.

Category	Definitions (example)
Lowest operationalisation level	Hate incidents are an encounter in which difference is perceived but is responded to with violence rather than care. Hate incidents are rooted in a confrontation with an other that seeks to violently reaffirm boundaries and identities through a refusal to become with and respond to that other's alterity (Gatehouse, 2020). I define ‘hate speech’, understood as words and expressions uttered with the aim of mortifying, denigrating, dehumanising and inferiorizing the people to whom they refer, as well as encouraging and fomenting prejudice, hostility, if not gratuitous violence against the chosen victims (translated from Italian language using Google Scholar; Lunaria, 2020a).
Medium‐low operationalisation level	Islamophobia is a form of intolerance that entails rejection, disrespect and contempt for Islam and, by extension, for Muslims. It feeds behaviours of hate, discrimination, hostility and even aggression and violence; It is expressed through prejudiced speeches, insults, messages of aversion and also fanatics who build scenarios where hate crimes or crimes can be committed, including crimes against humanity (translated from Spanish language using Google Scholar; Plataforma Ciudadana contra la Islamofobia, 2015). Racist hate propaganda is an organised dissemination of a malevolent doctrine of vilification and detestation of a group of individuals based on racial identification (Somers, 1993).
Medium‐high operationalisation level	Anti‐Christian hate crimes. Attacks or threats against people because of their actual or perceived Christian identity, or targeting persons or property associated with Christian people or communities, constitute anti‐Christian hate crimes. Such crimes can target both majority and minority Christian denominations. ODIHR's hate crime reporting suggests that minority Christian groups may be more often subjected to physical violence, while property may be the prime target where Christians are a majority group (Office for Democratic Institutions and Human Rights, 2018). Gender‐based hate crimes are criminal offences motivated by bias against a person's gender. Such crimes target people, property or associations connected with people or groups due to their actual or perceived gender (ODIHR, 2021b).
Highest operationalisation level	Hate speech is intentional or unintentional public discriminatory and/or defamatory statements; intentional incitement to hatred and/or violence and/or segregation based on a person's or a group's real or perceived race, ethnicity, language, nationality, skin colour, religious beliefs or lack thereof, gender, gender identity, sex, sexual orientation, political beliefs, social status, property, birth, age, mental health, disability, disease. You can find hate speech online, or in real life (International Network Against Cyber Hate, n.d.). Hate crime, then, involves acts of violence and intimidation, usually directed toward already stigmatised and marginalised groups. As such, it is a mechanism of power and oppression, intended to reaffirm the precarious hierarchies that characterise a given social order. It attempts to re‐create simultaneously the threatened (real or imagined) hegemony of the perpetrator's group and the ‘appropriate’ subordinate identity of the victim's group (Perry, 2002, 2009).

We found that hate crime definitions tend to more operationalisable than definitions of hate speech and behaviours across multiple categories). Specifically, 48% (*N* = 76) of hate crime definitions are either low or medium‐low level of operationalisability, compared to 68% (*N* = 79) of hate speech definitions, and 62% (*N* = 79) of definitions of behaviours across multiple categories. Conversely, 52% (*N* = 83) of hate crime definitions are either high or medium‐high level of operationalisability, compared to 32% (*N* = 37) of hate speech and 38% (*N* = 48) of definitions of behaviours across multiple categories.

#### 6.3.2 How theoretically solid and methodologically transparent are measurement tools?

In Table 32, we present a comparative view of the theoretical robustness and transparency of the eight types of measurement tools that we analysed in this project. This table maps strengths and weaknesses of different types of measurement tools, which are broadly associated with different disciplinary approaches, users and aims.

**Table 32:** Average percentage of measurement tools by indicator of theoretical solidity and methodological transparency.

	Truth	Discrimination	Feasibility	Uptake	Theoretical robustness	Transparency
Incidents reporting tools	52% (*N* = 11)	48% (*N* = 10)	5% (*N* = 1)	71% (*N* = 15)	62% (*N* = 13)	10% (*N* = 2)
Case records and open source databases	39% (*N* = 10)	46% (*N* = 12)	4% (*N* = 1)	27% (*N* = 7)	50% (*N* = 13)	15% (*N* = 4)
Quantitative survey questionnaires	26% (*N* = 14)	74% (*N* = 39)	30% (*N* = 16)	30% (*N* = 16)	57% (*N* = 30)	6% (*N* = 3)
Manual quantitative text analysis tools	43% (*N* = 6)	86% (*N* = 12)	7% (*N* = 1)	21% (*N* = 3)	86% (*N* = 12)	7% (*N* = 1)
Automated or semi‐automated text analysis tools	76% (*N* = 16)	43% (*N* = 9)	10% (*N* = 2)	0% (*N* = 0)	100% (*N* = 21)	43% (*N* = 9)
Qualitative interviews schedules	60% (*N* = 9)	60% (*N* = 9)	47% (*N* = 7)	13% (*N* = 2)	13% (*N* = 2)	0% (*N* = 0)
Qualitative text analysis tools	6% (*N* = 1)	83% (*N* = 1)	0% (*N* = 0)	11% (*N* = 2)	33% (*N* = 6)	1% (*N* = 1)

### 6.4 Potential biases in the review process

This is an unusual systematic review because its aim is not to synthetise research evidence (i.e., to synthetise the results of several studies). Rather, its aims are to identify and map the qualities of a string of text (i.e., a definition) and a methodological approach (i.e., a measurement tool), both of which can exist in absence of empirical data and research findings. This raised important challenges for the research team in terms of devising a new and more flexible templates, guidelines and review processes tailored for the aims of this project, in collaboration with the Campbell Collaboration Crime and Justice Coordinating Group and the Campbell Collaboration Methods Groups. The strategies that we adopted include:

- a flexible search strategy that includes an array of methodologies ranging from traditional systematic review techniques (e.g., keyword‐based searches of databases, citation and backward citation searching) and research methods techniques (e.g., interviews with experts);
- an original template of criteria to operationalise for the first time concepts like the ‘originality’ of a definition (see Section 5.1);
- tailored data extraction process to identify and extract definitions and measurement tools in a document (e.g., we analysed the paragraph before and after the definition, in order to capture additional important elements where present);
- tailored analysis and quality assessment criteria to appraise the quality of definitions and measurement tools (see Section 6.3);
- a new reporting template that reflects the original aims of this review.

This flexible and innovative approach allowed us to reduce the review bias and to achieve a more comprehensive mapping of definitions and measurement tools.

However, despite the strategies to limit bias in the review process that we thoroughly described in Section Inclusion Criteria, 4 of this report, we acknowledge some potential limitations of our review that could have biased our findings. Firstly, by defining our search strategy, we in some ways defined the scope of what we understand as a hate behaviour. For example, we only retrieved a marginal number of definitions looking at misogyny and sexism because we didn't search comprehensively the literature on domestic and sexual violence. We only found marginal overlaps between hate crimes and other forms of targeted violence (e.g., fixated violence, grievance‐fuelled violence) because we did not include these terms in our review. We didn't have the resources to do it as part of this project, but we believe that there is urgent need for more conceptual and empirical work on the relationships between hate crime, domestic violence and sexual violence. Secondly, some of our findings are limited by the availability of grey literature published in the 1990s. We were unable to retrieve 6% (*N* = 438) of the 7205 documents eligible for full text screening, most of which were research reports and theses published in the 1990s. However, given the large quantity of definitions and measurement tools collected, and given the long‐standing expertise and presence of our team members in this field of research, we are confident that we reached saturation of the types of definitions and measurement tools in this field.

### 6.5 Agreements and disagreements with other studies or reviews

Due to the innovative type and scope of this review, the findings do not directly reaffirm or contradict any existing review. A limited number of reviews looked at portions of the field that we mapped. For example, Schweppe (2021) reviewed definitions of hate crimes, Walters (2022) analysed hate crime legislation comparatively, and Hietanen and Eddebo (2022) reviewed definitions of hate speech in legislation and academic literature. We incorporated the conceptual frameworks of these studies in our work. However, we are not aware of any review or study providing a comprehensive comparative analysis of definitions and measurement tools between different bodies of literature and hate behaviours.

## 7 AUTHORS' CONCLUSIONS

### 7.1 Implications for practice

This project has numerous implications for practice, as it allows to formulate recommendations for future policy and programming related to the prevention of hate behaviours.

Firstly, there is an urgent need to close the gap between the protections of ‘ethnic and religious identities’ and other (less) protected characteristics. Our review shows that definitions and measurement tools used in research, legislation and grey area, are less likely to list characteristics included in the categories ‘gender and sexual identities’ and ‘disability, bodies and health’ (see 5.3.1.2 for a detailed list). This gap is significant in relation to hate crime, but even more pronounced in relation to hate speech definitions and measurement tools. Importantly, new categories of protected characteristics have started to emerge in recent years – including ‘social class’, ‘ideology and occupation’ – which need attention, assessment and review in future policy and programming work. This is vital to make sure that all victims of hate crime are treated equitably across jurisdictions.

Secondly, there is a need to overcome a siloed approach amongst government and nongovernment organisations working in this field. Despite increasing attention to hate crime across both sectors, the only example of formal and documented collaboration between government and nongovernment organisations within a single jurisdiction in collecting hate crime data is True Vision UK, which published the signed agreements that regulate data sharing between the government and two community organisations. We propose that more collaborations of this kind should be implemented globally. Also, there is urgent need to step up dialogue across borders amongst government, practitioners, and academic sectors, to cross‐pollinate best practices and lessons learned.

Thirdly, it is crucial that more data collected by government and nongovernment organisations is made available for research purposes. Our review identified only limited measurement tools that were made available to research either privately (e.g., via allowing researchers to study case records) or publicly (e.g., via publishing the de‐identified data collected via an IRT). Pursuing these collaborations would create valuable opportunities to enhance the quality of data; enabling for researchers to contribute to the streamlining of data collection processes and improvement of reliability and validity of measurements. This would result in numerous flow‐on effects including increased ability to evaluate the impact of policy and programmes and the creation of more responsive legislative tools to protect communities.

Fourthly, our review points to the need to conduct a more thorough assessment of definitions found in legislation and of country‐level measurement tools used by government and nongovernment organisations (e.g., official police sources, IRTs used by government and nongovernment organisations) using a different research methodology. This project provides an important baseline for conducting a formal evaluation, but other methodologies such as extensive interviews, surveys, content analysis of guidelines used by frontline workers and training materials, and ethnographic methods should be employed to assess legal definitions and measurement tools used by government and nongovernment organisations.

### 7.2 Implications for research

Based on our review, we offer general recommendations for future research.

Firstly, as in the recommendations for practice, we suggest that future research should increase the focus on hate against communities that are comparatively under‐researched, including, for example, people with disability, gender‐diverse people, women, and LGBTQIA+ people. This does not mean that we need less research on racism, antisemitism and Islamophobia; especially as they remain the most prevalent form of hate crime in most jurisdictions. However, it is crucial that we develop a greater understanding of the forms of hate targeting other key protected identities, in order to understand the effects of hate in society as a whole, and to contextualise existing knowledge (for instance, by examining whether the aetiology and effects of other forms of hate, are similar to racist and religious hate). Also, there is a need for more research and conceptual work to analyse emerging forms of hate against identities defined by social class, ideology and occupation in the contemporary context, to understand whether and how these emerging identities should be more widely included in protected characteristics in legislation and policy.

Secondly, there is an urgent need to improve the quality of methodological and reporting standards in research examining hate behaviours, including transparency in methodology and data reporting, and discussion of limitations (e.g., bias in data). Many of the measurement tools found in the academic literature were excluded because they did not report transparently how they collected and analysed the data. Further, 41% of documents presenting research on hate behaviours did not provide a definition of what they were looking at. This lack of empirical and conceptual rigour and transparency affects the quality of the results produced by academics in this field, potentially contributing to the delegitimisation of academic work in public opinion. Given the importance of this policy domain, it is vital to raise the quality and trustworthiness of research in this area.

Thirdly, there is strong need for mixed methods research assessing the quality, validity and reliability of different measurement tools. We found only a very limited number of studies trying to assess existing measurement tools or appraise the quality of different data sources, comparing them (e.g., police data and civil society organisation data, police data and victimisation survey data) to understand differences, strengths and limitations.

Fourthly, our results show that academic research is affected by a disciplinary fragmentation and silo mentality. We identified hundreds of definitions that – in most cases – are just slight variations on a few themes: they might refer to slightly different protected characteristics, or use a slightly different term to qualify the hate element (e.g., ‘hate’ vs. ‘bias’ vs. ‘prejudice’). Moreover, our review found that researchers in different disciplinary areas (e.g., social sciences and computer science) rarely collaborate. Future research should attempt to build on existing definitions and measurement tools (instead of duplicating efforts), and engage in more interdisciplinary collaborations. These recommendations might be used by funding agencies in devising future grant schemes on this important policy area.

In conclusion, this review has mapped current scientific approaches to defining and measuring hate across academic, government, and non‐government sectors. It has provided recommendations for practice and research, and put forward a comprehensive list of conceptual and empirical tools that can be used by future generations of research. It is our hope that that this review can provide a solid foundation for researchers, government, and other bodies to build cumulative knowledge and collaboration in this important field.

## CONTRIBUTIONS OF AUTHORS

- Content: Vergani, Perry, Freilich, Chermak, Scrivens, Link, Betts, Iqbal, Kleinsman
- Legislation data extraction and analysis: Kleinsman
- Social network analysis: Iqbal
- Computer science data extraction and data analysis: Betts, Iqbal
- Systematic review methods: Vergani, Perry, Freilich, Chermak, Scrivens
- Information retrieval, screening, data extraction: authors and research assistants

## DECLARATIONS OF INTEREST

None.

## PRELIMINARY TIMEFRAME

The final report was submitted 28 February 2023.

## PLANS FOR UPDATING THE REVIEW

None.

## SOURCES OF SUPPORT

This review was funded by a Campbell Collaboration grant awarded to Vergani, Perry, Freilich, Chermak, Scrivens via Public Safety Canada.

## Supporting information

APPENDIX 1 Definitions data.

APPENDIX 2 Measurement tools data.

APPENDIX 3 Legislation data.

APPENDIX 4 Overview data extraction tools.

APPENDIX 5 Full search strategy.

APPENDIX 6 Complementary search methods.
