# Supplementary material for: Mapping the scientific knowledge and approaches to defining and measuring hate crime, hate speech, and hate incidents: A systematic review
Source: Campbell Syst Rev. 2024 Apr 28;20(2):e1397. doi: 10.1002/cl2.1397 (PMC11056620; doi:10.1002/cl2.1397)
Supplement: Supplementary file 5 — APPENDIX 5 Full search strategy. [file CL2-20-e1397-s005.docx]

Appendix: Full search strategy and results

# Group 1: Databases that allow for complex searches

## EBSCOhost databases

- Communications and Mass Media Complete
- Criminal Justice Abstracts
- SocIndex

| Set# | Search syntax |
| --- | --- |
| S1 | (TI((hate OR prejudice* OR bias* OR racis* OR xenophobi* OR sinophobi* OR anti-foreigner OR anti-migrant* OR anti-immigrant* OR anti-refugee* OR “anti-asylum seeker” OR anti-Roma OR anti-traveller OR anti-Gypsy OR “anti-First Nations” OR anti-Indigenous OR anti-Maori OR anti-Aboriginal OR islamophobi* OR antisemiti* OR anti-semiti* OR anti-Jew* OR anti-Amish OR anti-Sikh OR anti-Buddhis* OR anti-Muslim* OR anti-Islam* OR anti-Christian* OR homophobi* OR transphobi* OR lesbophobi* OR biphobi* OR anti-gay OR anti-lesbian* OR anti-bisex* OR anti-transgender OR anti-LGBT* OR ableis* OR disableis* OR sexis* OR mysogyn* OR misandr* OR gender-based OR “gendered” OR incel OR invcel OR “involuntary celibate” OR anti-feminis* OR anti-abortion OR anti-doctor OR “anti-sex worker” OR anti-politician) N2 ((crim* OR speech OR incident* OR conduct OR act OR abus* OR vilif* OR language* OR violen* OR rape* OR murder* OR harass* OR terroris* OR narrative* OR discourse* OR propaganda OR “targeted violence” OR incite* OR extremis* OR hostil* OR micro-aggression or microaggression OR group*))) OR (AB((hate OR prejudice* OR bias* OR racis* OR xenophobi* OR sinophobi* OR anti-foreigner OR anti-migrant* OR anti-immigrant* OR anti-refugee* OR “anti-asylum seeker” OR anti-Roma OR anti-traveller OR anti-Gypsy OR “anti-First Nations” OR anti-Indigenous OR anti-Maori OR anti-Aboriginal OR islamophobi* OR antisemiti* OR anti-semiti* OR anti-Jew* OR anti-Amish OR anti-Sikh OR anti-Buddhis* OR anti-Muslim* OR anti-Islam* OR anti-Christian* OR homophobi* OR transphobi* OR lesbophobi* OR biphobi* OR anti-gay OR anti-lesbian* OR anti-bisex* OR anti-transgender OR anti-LGBT* OR ableis* OR disableis* OR sexis* OR mysogyn* OR misandr* OR gender-based OR “gendered” OR incel OR invcel OR “involuntary celibate” OR anti-feminis* OR anti-abortion OR anti-doctor OR “anti-sex worker” OR anti-politician) N2 ((crim* OR speech OR incident* OR conduct OR act OR abus* OR vilif* OR language* OR violen* OR rape* OR murder* OR harass* OR terroris* OR narrative* OR discourse* OR propaganda OR “targeted violence” OR incite* OR extremis* OR hostil* OR micro-aggression or microaggression OR group*))) OR (KW((hate OR prejudice* OR bias* OR racis* OR xenophobi* OR sinophobi* OR anti-foreigner OR anti-migrant* OR anti-immigrant* OR anti-refugee* OR “anti-asylum seeker” OR anti-Roma OR anti-traveller OR anti-Gypsy OR “anti-First Nations” OR anti-Indigenous OR anti-Maori OR anti-Aboriginal OR islamophobi* OR antisemiti* OR anti-semiti* OR anti-Jew* OR anti-Amish OR anti-Sikh OR anti-Buddhis* OR anti-Muslim* OR anti-Islam* OR anti-Christian* OR homophobi* OR transphobi* OR lesbophobi* OR biphobi* OR anti-gay OR anti-lesbian* OR anti-bisex* OR anti-transgender OR anti-LGBT* OR ableis* OR disableis* OR sexis* OR mysogyn* OR misandr* OR gender-based OR “gendered” OR incel OR invcel OR “involuntary celibate” OR anti-feminis* OR anti-abortion OR anti-doctor OR “anti-sex worker” OR anti-politician) N2 (crim* OR speech OR incident* OR conduct OR act OR abus* OR vilif* OR language* OR violen* OR rape* OR murder* OR harass* OR terroris* OR narrative* OR discourse* OR propaganda OR “targeted violence” OR incite* OR extremis* OR hostil* OR micro-aggression or microaggression OR group*))) OR (SU((hate OR prejudice* OR bias* OR racis* OR xenophobi* OR sinophobi* OR anti-foreigner OR anti-migrant* OR anti-immigrant* OR anti-refugee* OR “anti-asylum seeker” OR anti-Roma OR anti-traveller OR anti-Gypsy OR “anti-First Nations” OR anti-Indigenous OR anti-Maori OR anti-Aboriginal OR islamophobi* OR antisemiti* OR anti-semiti* OR anti-Jew* OR anti-Amish OR anti-Sikh OR anti-Buddhis* OR anti-Muslim* OR anti-Islam* OR anti-Christian* OR homophobi* OR transphobi* OR lesbophobi* OR biphobi* OR anti-gay OR anti-lesbian* OR anti-bisex* OR anti-transgender OR anti-LGBT* OR ableis* OR disableis* OR sexis* OR mysogyn* OR misandr* OR gender-based OR “gendered” OR incel OR invcel OR “involuntary celibate” OR anti-feminis* OR anti-abortion OR anti-doctor OR “anti-sex worker” OR anti-politician) N2 (crim* OR speech OR incident* OR conduct OR act OR abus* OR vilif* OR language* OR violen* OR rape* OR murder* OR harass* OR terroris* OR narrative* OR discourse* OR propaganda OR “targeted violence” OR incite* OR extremis* OR hostil* OR micro-aggression or microaggression OR group*))) |
| S2 | (TI(“United States” OR “US” OR “USA” OR Australia* OR “New Zealand*” OR Aotearoa OR France OR French OR German* OR Irish OR Ireland OR Ital* OR Spain OR Spanish OR “UK” OR “United Kingdom” OR Brit* OR Engl* OR “Northern Ireland” OR “Northern Irish” OR Scot* OR Wales OR Welsh OR Canad*)) OR (AB(“United States” OR “US” OR “USA” OR Australia* OR “New Zealand*” OR Aotearoa OR France OR French OR German* OR Irish OR Ireland OR Ital* OR Spain OR Spanish OR “UK” OR “United Kingdom” OR Brit* OR Engl* OR “Northern Ireland” OR “Northern Irish” OR Scot* OR Wales OR Welsh OR Canad*)) OR (KW(“United States” OR “US” OR “USA” OR Australia* OR “New Zealand*” OR Aotearoa OR France OR French OR German* OR Irish OR Ireland OR Ital* OR Spain OR Spanish OR “UK” OR “United Kingdom” OR Brit* OR Engl* OR “Northern Ireland” OR “Northern Irish” OR Scot* OR Wales OR Welsh OR Canad*)) OR (SU(“United States” OR “US” OR “USA” OR Australia* OR “New Zealand*” OR Aotearoa OR France OR French OR German* OR Irish OR Ireland OR Ital* OR Spain OR Spanish OR “UK” OR “United Kingdom” OR Brit* OR Engl* OR “Northern Ireland” OR “Northern Irish” OR Scot* OR Wales OR Welsh OR Canad*)) |
| S3 | S1 AND S2 |

| Date | Database | Language limiters | Date limiters | Document type limiters | Discipline limiters | Number of search results | Number of results imported into EndNote | Comments |
| --- | --- | --- | --- | --- | --- | --- | --- | --- |
| 18/03/2022 | Communications and Mass Media Complete | English, French, German, Spanish, multiple languages | 1990-2021 | N/A | N/A | 729 | 729 |  |
| 19/03/2022 | Criminal Justice Abstracts | English, French, German, Italian, Spanish | 1990-2021 | Academic journals, Trade publications, Books, Reviews | N/A | 1195 | 1195 |  |
| 19/03/2022 | SocIndex | English, French, German, Italian, Spanish | 1990-2021 | Academic journals, Trade publications, Books, Conference Papers, Dissertations, Reviews | N/A | 3079 | 3080 | To support reference screening by language, we exported references from SocIndex grouped by language. The discrepancy between the number of search results retrieved through the search and that imported into EndNote is likely a result of doing so. |

## ProQuest databases

- Continental Europe Database
- Dissertations & Theses Global
- ERIC
- Sociological Abstracts
- Technology Collection

| Set# | Search syntax |
| --- | --- |
| S1 | (AB("United States" OR "US" OR "USA" OR Australia* OR "New Zealand*" OR Aotearoa OR France OR French OR German* OR Irish OR Ireland OR Ital* OR Spain OR Spanish OR "UK" OR "United Kingdom" OR Brit* OR Engl* OR "Northern Ireland" OR "Northern Irish" OR Scot* OR Wales OR Welsh OR Canad*) OR TI("United States" OR "US" OR "USA" OR Australia* OR "New Zealand*" OR Aotearoa OR France OR French OR German* OR Irish OR Ireland OR Ital* OR Spain OR Spanish OR "UK" OR "United Kingdom" OR Brit* OR Engl* OR "Northern Ireland" OR "Northern Irish" OR Scot* OR Wales OR Welsh OR Canad*) OR MAINSUBJECT("United States" OR "US" OR "USA" OR Australia* OR "New Zealand*" OR Aotearoa OR France OR French OR German* OR Irish OR Ireland OR Ital* OR Spain OR Spanish OR "UK" OR "United Kingdom" OR Brit* OR Engl* OR "Northern Ireland" OR "Northern Irish" OR Scot* OR Wales OR Welsh OR Canad*)) |
| S2 | (TI(hate OR prejudice* OR bias* OR racis* OR xenophobi* OR sinophobi* OR anti-foreigner* OR anti-migrant* OR anti-immigrant* OR anti-refugee* OR "anti-asylum seeker" OR anti-Roma OR anti-traveller OR anti-Gypsy OR "anti-First Nations" OR anti-Indigenous OR anti-Maori OR anti-Aboriginal OR islamophobi* OR anti-semiti* OR anti-Jew* OR anti-Amish OR anti-Sikh OR anti-Buddhis* OR anti-Muslim* OR anti-Islam* OR anti-Christian* OR homophobi* OR transphobi* OR lesbophobi* OR biphobi* OR anti-gay OR anti-lesbian* OR anti-bisex* OR anti-transgender OR anti-LGBT* OR ableis* OR disableis* OR sexis* OR mysogyn* OR misandr* OR gender-based OR "gendered" OR incel OR invcel OR "involuntary celibate" OR anti-feminis* OR anti-abortion OR anti-doctor OR "anti-sex worker" OR anti-politician)) NEAR/2 (TI(crim* OR speech OR incident* OR conduct OR act OR abus* OR vilif* OR language* OR violen* OR rape* OR murder* OR harass* OR terroris* OR narrative* OR discourse* OR propaganda OR "targeted violence" OR incite* OR extremis* OR hostil* OR microaggression* OR micro-aggression* OR group*)) |
| S3 | (AB(hate OR prejudice* OR bias* OR racis* OR xenophobi* OR sinophobi* OR anti-foreigner* OR anti-migrant* OR anti-immigrant* OR anti-refugee* OR "anti-asylum seeker" OR anti-Roma OR anti-traveller OR anti-Gypsy OR "anti-First Nations" OR anti-Indigenous OR anti-Maori OR anti-Aboriginal OR islamophobi* OR anti-semiti* OR anti-Jew* OR anti-Amish OR anti-Sikh OR anti-Buddhis* OR anti-Muslim* OR anti-Islam* OR anti-Christian* OR homophobi* OR transphobi* OR lesbophobi* OR biphobi* OR anti-gay OR anti-lesbian* OR sex* OR anti-transgender OR anti-LGBT* OR ableis* OR disableis* OR sexis* OR mysogyn* OR misandr* OR gender-based OR "gendered" OR incel OR invcel OR "involuntary celibate" OR anti-feminis* OR anti-abortion OR anti-doctor OR "anti-sex worker" OR anti-politician)) NEAR/2 (AB(crim* OR speech OR incident* OR conduct OR act OR abus* OR vilif* OR language* OR violen* OR rape* OR murder* OR harass* OR terroris* OR narrative* OR discourse* OR propaganda OR "targeted violence" OR incite* OR extremis* OR hostil* OR microaggression* OR micro-aggression* OR group*)) |
| S4 | (MAINSUBJECT(hate OR prejudice* OR bias* OR racis* OR xenophobi* OR sinophobi* OR anti-foreigner* OR anti-migrant* OR anti-immigrant* OR anti-refugee* OR "anti-asylum seeker" OR anti-Roma OR anti-traveller OR anti-Gypsy OR "anti-First Nations" OR anti-Indigenous OR anti-Maori OR anti-Aboriginal OR islamophobi* OR anti-semiti* OR anti-Jew* OR anti-Amish OR anti-Sikh OR anti-Buddhis* OR anti-Muslim* OR anti-Islam* OR anti-Christian* OR homophobi* OR transphobi* OR lesbophobi* OR biphobi* OR anti-gay OR anti-lesbian* OR sex* OR anti-transgender OR anti-LGBT* OR ableis* OR disableis* OR sexis* OR mysogyn* OR misandr* OR gender-based OR "gendered" OR incel OR invcel OR "involuntary celibate" OR anti-feminis* OR anti-abortion OR anti-doctor OR "anti-sex worker" OR anti-politician)) NEAR/2 (MAINSUBJECT(crim* OR speech OR incident* OR conduct OR act OR abus* OR vilif* OR language* OR violen* OR rape* OR murder* OR harass* OR terroris* OR narrative* OR discourse* OR propaganda OR "targeted violence" OR incite* OR extremis* OR hostil* OR microaggression* OR micro-aggression* OR group*)) |
| S5 | S1 AND S2 |
| S6 | S1 AND S3 |
| S7 | S1 AND S4 |
| S8 | S5 OR S6 OR S7 |

| **Date of search** | **Database** | **Language limiters** | **Date limiters** | **Document type limiters** | **Discipline limiters** | **Number of search results** | **Number of results imported into EndNote** | **Comments** |
| --- | --- | --- | --- | --- | --- | --- | --- | --- |
| 31/03/2022 | Sociological Abstracts | English, French, German, Italian, Spanish | 1990-2021 | N/A | N/A | 4374 | 4380 | We excluded "General Information", "News" and "Poems" from search results. |
| 12/04/2022 | ERIC | English, French, German, Italian, Spanish | 1990-2021 | N/A | N/A | 929 | 932 | To support reference screening by language, we exported references from ERIC grouped by language. The discrepancy between the number of search results retrieved through the search and that imported into EndNote is likely a result of doing so. |
| 12/04/2022 | Dissertations & Theses Global | English, French, German, Italian, Spanish | 1990-2021 | N/A | N/A | 2299 | 2299 |  |
| 12/04/2022 | Technology Collection | English, French, German, Italian, Spanish | 1990-2021 | Scholarly journals, Conference papers & proceedings, Working papers, Other sources, Reports, Books | N/A | 1265 | 1265 |  |

## Ovid

- PsycInfo

| Set# | Search syntax |
| --- | --- |
| 1 | ((hate or prejudice* or bias* or racis* or xenophobi* or sinophobi* or anti-foreigner* or  anti-migrant* or anti-immigrant* or anti-refugee* or "anti-asylum seeker" or anti-  Roma or anti-traveller or anti-Gypsy or "anti-First Nations" or anti-Indigenous or  anti-Maori or anti-Aboriginal or islamophobi* or anti-semiti* or anti-Jew* or anti-  Amish or anti-Sikh or anti-Buddhis* or anti-Muslim* or anti-Islam* or anti-Christian*  or homophobi* or transphobi* or lesbophobi* or biphobi* or anti-gay or anti-lesbian*  or anti-bisex* or anti-transgender or anti-LGBT* or ableis* or disableis* or sexis* or  mysogyn* or misandr* or gender-based or "gendered" or incel or invcel or  "involuntary celibate" or anti-feminis* or anti-abortion or anti-doctor or "anti-sex  worker" or anti-politician) adj3 (crim* or speech or incident* or conduct or act or  abus* or vilif* or language* or violen* or rape* or murder* or harass* or terroris* or  narrative* or discourse* or propaganda or "targeted violence" or incite* or extremis*  or hostil* or microaggression* or micro-aggression* or group*)).ab,hw,id,mh,ot,ti. |
| 2 | ("United States" OR "US" OR "USA" OR Australia* OR "New Zealand*" OR Aotearoa OR France OR French OR German* OR Irish OR Ireland OR Ital* OR Spain OR Spanish OR "UK" OR "United Kingdom" OR Brit* OR Engl* OR "Northern Ireland" OR "Northern Irish" OR Scot* OR Wales OR Welsh OR Canad*).ab,hw,id,mh,ot,ti. |
| 3 | 1 and 2 |
| 4 | limit 3 to yr=”1990-2021” |
| 5 | limit 4 to (english or french or german or italian or spanish) |

| **Date of search** | **Database** | **Language limiters** | **Date limiters** | **Document type limiters** | **Discipline limiters** | **Number of search results** | **Number of results imported into EndNote** | **Comments** |
| --- | --- | --- | --- | --- | --- | --- | --- | --- |
|  |  |  |  |  |  |  |  |  |
| 31/03/2022 | PsycInfo | English, French, German, Italian, Spanish | 1990-2021 | N/A | N/A | 3342 | 3342 |  |

## Web of Science

- Web of Science Core Collection
- SciELO Citation Index

| Set# | Search syntax |
| --- | --- |
| #1 | TS=(hate NEAR/2 (crim* OR speech OR incident* OR conduct OR act OR abus* OR vilif* OR language* OR violen* OR rape* OR murder* OR harass* OR terroris* OR narrative* OR discourse* OR propaganda OR “targeted violence” OR incite* OR extremis* OR hostil* OR micro-aggression or microaggression OR group*)) |
| #2 | TS=(prejudice* NEAR/2 (crim* OR speech OR incident* OR conduct OR act OR abus* OR vilif* OR language* OR violen* OR rape* OR murder* OR harass* OR terroris* OR narrative* OR discourse* OR propaganda OR “targeted violence” OR incite* OR extremis* OR hostil* OR micro-aggression or microaggression OR group*)) |
| #3 | TS=(bias* NEAR/2 (crim* OR speech OR incident* OR conduct OR act OR abus* OR vilif* OR language* OR violen* OR rape* OR murder* OR harass* OR terroris* OR narrative* OR discourse* OR propaganda OR “targeted violence” OR incite* OR extremis* OR hostil* OR micro-aggression or microaggression OR group*)) |
| #4 | TS=(racis* NEAR/2 (crim* OR speech OR incident* OR conduct OR act OR abus* OR vilif* OR language* OR violen* OR rape* OR murder* OR harass* OR terroris* OR narrative* OR discourse* OR propaganda OR “targeted violence” OR incite* OR extremis* OR hostil* OR micro-aggression or microaggression OR group*)) |
| #5 | TS=(xenophobi* NEAR/2 (crim* OR speech OR incident* OR conduct OR act OR abus* OR vilif* OR language* OR violen* OR rape* OR murder* OR harass* OR terroris* OR narrative* OR discourse* OR propaganda OR “targeted violence” OR incite* OR extremis* OR hostil* OR micro-aggression or microaggression OR group*)) |
| #6 | TS=(sinophobi* NEAR/2 (crim* OR speech OR incident* OR conduct OR act OR abus* OR vilif* OR language* OR violen* OR rape* OR murder* OR harass* OR terroris* OR narrative* OR discourse* OR propaganda OR “targeted violence” OR incite* OR extremis* OR hostil* OR micro-aggression or microaggression OR group*)) |
| #7 | TS=(anti-foreigner NEAR/2 (crim* OR speech OR incident* OR conduct OR act OR abus* OR vilif* OR language* OR violen* OR rape* OR murder* OR harass* OR terroris* OR narrative* OR discourse* OR propaganda OR “targeted violence” OR incite* OR extremis* OR hostil* OR micro-aggression or microaggression OR group*)) |
| #8 | TS=(anti-migrant* NEAR/2 (crim* OR speech OR incident* OR conduct OR act OR abus* OR vilif* OR language* OR violen* OR rape* OR murder* OR harass* OR terroris* OR narrative* OR discourse* OR propaganda OR “targeted violence” OR incite* OR extremis* OR hostil* OR micro-aggression or microaggression OR group*)) |
| #9 | TS=(anti-immigrant* NEAR/2 (crim* OR speech OR incident* OR conduct OR act OR abus* OR vilif* OR language* OR violen* OR rape* OR murder* OR harass* OR terroris* OR narrative* OR discourse* OR propaganda OR “targeted violence” OR incite* OR extremis* OR hostil* OR micro-aggression or microaggression OR group*)) |
| #10 | TS=(anti-refugee* NEAR/2 (crim* OR speech OR incident* OR conduct OR act OR abus* OR vilif* OR language* OR violen* OR rape* OR murder* OR harass* OR terroris* OR narrative* OR discourse* OR propaganda OR “targeted violence” OR incite* OR extremis* OR hostil* OR micro-aggression or microaggression OR group*)) |
| #11 | TS=(“anti-asylum seeker” NEAR/2 (crim* OR speech OR incident* OR conduct OR act OR abus* OR vilif* OR language* OR violen* OR rape* OR murder* OR harass* OR terroris* OR narrative* OR discourse* OR propaganda OR “targeted violence” OR incite* OR extremis* OR hostil* OR micro-aggression or microaggression OR group*)) |
| #12 | TS=(anti-Roma NEAR/2 (crim* OR speech OR incident* OR conduct OR act OR abus* OR vilif* OR language* OR violen* OR rape* OR murder* OR harass* OR terroris* OR narrative* OR discourse* OR propaganda OR “targeted violence” OR incite* OR extremis* OR hostil* OR micro-aggression or microaggression OR group*)) |
| #13 | TS=(anti-traveller NEAR/2 (crim* OR speech OR incident* OR conduct OR act OR abus* OR vilif* OR language* OR violen* OR rape* OR murder* OR harass* OR terroris* OR narrative* OR discourse* OR propaganda OR “targeted violence” OR incite* OR extremis* OR hostil* OR micro-aggression or microaggression OR group*)) |
| #14 | TS=(anti-Gypsy NEAR/2 (crim* OR speech OR incident* OR conduct OR act OR abus* OR vilif* OR language* OR violen* OR rape* OR murder* OR harass* OR terroris* OR narrative* OR discourse* OR propaganda OR “targeted violence” OR incite* OR extremis* OR hostil* OR micro-aggression or microaggression OR group*)) |
| #15 | TS=(“anti-First Nations” NEAR/2 (crim* OR speech OR incident* OR conduct OR act OR abus* OR vilif* OR language* OR violen* OR rape* OR murder* OR harass* OR terroris* OR narrative* OR discourse* OR propaganda OR “targeted violence” OR incite* OR extremis* OR hostil* OR micro-aggression or microaggression OR group*)) |
| #16 | TS=(anti-Indigenous NEAR/2 (crim* OR speech OR incident* OR conduct OR act OR abus* OR vilif* OR language* OR violen* OR rape* OR murder* OR harass* OR terroris* OR narrative* OR discourse* OR propaganda OR “targeted violence” OR incite* OR extremis* OR hostil* OR micro-aggression or microaggression OR group*)) |
| #17 | TS=(anti-Maori NEAR/2 (crim* OR speech OR incident* OR conduct OR act OR abus* OR vilif* OR language* OR violen* OR rape* OR murder* OR harass* OR terroris* OR narrative* OR discourse* OR propaganda OR “targeted violence” OR incite* OR extremis* OR hostil* OR micro-aggression or microaggression OR group*)) |
| #18 | TS=(anti-Aboriginal NEAR/2 (crim* OR speech OR incident* OR conduct OR act OR abus* OR vilif* OR language* OR violen* OR rape* OR murder* OR harass* OR terroris* OR narrative* OR discourse* OR propaganda OR “targeted violence” OR incite* OR extremis* OR hostil* OR micro-aggression or microaggression OR group*)) |
| #19 | TS=(islamophobi* NEAR/2 (crim* OR speech OR incident* OR conduct OR act OR abus* OR vilif* OR language* OR violen* OR rape* OR murder* OR harass* OR terroris* OR narrative* OR discourse* OR propaganda OR “targeted violence” OR incite* OR extremis* OR hostil* OR micro-aggression or microaggression OR group*)) |
| #20 | TS=(antisemiti* NEAR/2 (crim* OR speech OR incident* OR conduct OR act OR abus* OR vilif* OR language* OR violen* OR rape* OR murder* OR harass* OR terroris* OR narrative* OR discourse* OR propaganda OR “targeted violence” OR incite* OR extremis* OR hostil* OR micro-aggression or microaggression OR group*)) |
| #21 | TS=(anti-Semiti* NEAR/2 (crim* OR speech OR incident* OR conduct OR act OR abus* OR vilif* OR language* OR violen* OR rape* OR murder* OR harass* OR terroris* OR narrative* OR discourse* OR propaganda OR “targeted violence” OR incite* OR extremis* OR hostil* OR micro-aggression or microaggression OR group*)) |
| #22 | TS=(anti-Jew* NEAR/2 (crim* OR speech OR incident* OR conduct OR act OR abus* OR vilif* OR language* OR violen* OR rape* OR murder* OR harass* OR terroris* OR narrative* OR discourse* OR propaganda OR “targeted violence” OR incite* OR extremis* OR hostil* OR micro-aggression or microaggression OR group*)) |
| #23 | TS=(anti-Amish NEAR/2 (crim* OR speech OR incident* OR conduct OR act OR abus* OR vilif* OR language* OR violen* OR rape* OR murder* OR harass* OR terroris* OR narrative* OR discourse* OR propaganda OR “targeted violence” OR incite* OR extremis* OR hostil* OR micro-aggression or microaggression OR group*)) |
| #24 | TS=(anti-Sikh NEAR/2 (crim* OR speech OR incident* OR conduct OR act OR abus* OR vilif* OR language* OR violen* OR rape* OR murder* OR harass* OR terroris* OR narrative* OR discourse* OR propaganda OR “targeted violence” OR incite* OR extremis* OR hostil* OR micro-aggression or microaggression OR group*)) |
| #25 | TS=(anti-Buddhis* NEAR/2 (crim* OR speech OR incident* OR conduct OR act OR abus* OR vilif* OR language* OR violen* OR rape* OR murder* OR harass* OR terroris* OR narrative* OR discourse* OR propaganda OR “targeted violence” OR incite* OR extremis* OR hostil* OR micro-aggression or microaggression OR group*)) |
| #26 | TS=(anti-Muslim* NEAR/2 (crim* OR speech OR incident* OR conduct OR act OR abus* OR vilif* OR language* OR violen* OR rape* OR murder* OR harass* OR terroris* OR narrative* OR discourse* OR propaganda OR “targeted violence” OR incite* OR extremis* OR hostil* OR micro-aggression or microaggression OR group*)) |
| #27 | TS=(anti-Islam* NEAR/2 (crim* OR speech OR incident* OR conduct OR act OR abus* OR vilif* OR language* OR violen* OR rape* OR murder* OR harass* OR terroris* OR narrative* OR discourse* OR propaganda OR “targeted violence” OR incite* OR extremis* OR hostil* OR micro-aggression or microaggression OR group*)) |
| #28 | TS=(anti-Christian* NEAR/2 (crim* OR speech OR incident* OR conduct OR act OR abus* OR vilif* OR language* OR violen* OR rape* OR murder* OR harass* OR terroris* OR narrative* OR discourse* OR propaganda OR “targeted violence” OR incite* OR extremis* OR hostil* OR micro-aggression or microaggression OR group*)) |
| #29 | TS=(homophobi* NEAR/2 (crim* OR speech OR incident* OR conduct OR act OR abus* OR vilif* OR language* OR violen* OR rape* OR murder* OR harass* OR terroris* OR narrative* OR discourse* OR propaganda OR “targeted violence” OR incite* OR extremis* OR hostil* OR micro-aggression or microaggression OR group*)) |
| #30 | TS=(transphobi* NEAR/2 (crim* OR speech OR incident* OR conduct OR act OR abus* OR vilif* OR language* OR violen* OR rape* OR murder* OR harass* OR terroris* OR narrative* OR discourse* OR propaganda OR “targeted violence” OR incite* OR extremis* OR hostil* OR micro-aggression or microaggression OR group*)) |
| #31 | TS=(lesbophobi* NEAR/2 (crim* OR speech OR incident* OR conduct OR act OR abus* OR vilif* OR language* OR violen* OR rape* OR murder* OR harass* OR terroris* OR narrative* OR discourse* OR propaganda OR “targeted violence” OR incite* OR extremis* OR hostil* OR micro-aggression or microaggression OR group*)) |
| #32 | TS=(biphobi* NEAR/2 (crim* OR speech OR incident* OR conduct OR act OR abus* OR vilif* OR language* OR violen* OR rape* OR murder* OR harass* OR terroris* OR narrative* OR discourse* OR propaganda OR “targeted violence” OR incite* OR extremis* OR hostil* OR micro-aggression or microaggression OR group*)) |
| #33 | TS=(anti-gay NEAR/2 (crim* OR speech OR incident* OR conduct OR act OR abus* OR vilif* OR language* OR violen* OR rape* OR murder* OR harass* OR terroris* OR narrative* OR discourse* OR propaganda OR “targeted violence” OR incite* OR extremis* OR hostil* OR micro-aggression or microaggression OR group*)) |
| #34 | TS=(anti-lesbian* NEAR/2 (crim* OR speech OR incident* OR conduct OR act OR abus* OR vilif* OR language* OR violen* OR rape* OR murder* OR harass* OR terroris* OR narrative* OR discourse* OR propaganda OR “targeted violence” OR incite* OR extremis* OR hostil* OR micro-aggression or microaggression OR group*)) |
| #35 | TS=(anti-bisex* NEAR/2 (crim* OR speech OR incident* OR conduct OR act OR abus* OR vilif* OR language* OR violen* OR rape* OR murder* OR harass* OR terroris* OR narrative* OR discourse* OR propaganda OR “targeted violence” OR incite* OR extremis* OR hostil* OR micro-aggression or microaggression OR group*)) |
| #36 | TS=(anti-transgender NEAR/2 (crim* OR speech OR incident* OR conduct OR act OR abus* OR vilif* OR language* OR violen* OR rape* OR murder* OR harass* OR terroris* OR narrative* OR discourse* OR propaganda OR “targeted violence” OR incite* OR extremis* OR hostil* OR micro-aggression or microaggression OR group*)) |
| #37 | TS=(anti-LGBT* NEAR/2 (crim* OR speech OR incident* OR conduct OR act OR abus* OR vilif* OR language* OR violen* OR rape* OR murder* OR harass* OR terroris* OR narrative* OR discourse* OR propaganda OR “targeted violence” OR incite* OR extremis* OR hostil* OR micro-aggression or microaggression OR group*)) |
| #38 | TS=(ableis* NEAR/2 (crim* OR speech OR incident* OR conduct OR act OR abus* OR vilif* OR language* OR violen* OR rape* OR murder* OR harass* OR terroris* OR narrative* OR discourse* OR propaganda OR “targeted violence” OR incite* OR extremis* OR hostil* OR micro-aggression or microaggression OR group*)) |
| #39 | TS=(disableis* NEAR/2 (crim* OR speech OR incident* OR conduct OR act OR abus* OR vilif* OR language* OR violen* OR rape* OR murder* OR harass* OR terroris* OR narrative* OR discourse* OR propaganda OR “targeted violence” OR incite* OR extremis* OR hostil* OR micro-aggression or microaggression OR group*)) |
| #40 | TS=(sexis* NEAR/2 (crim* OR speech OR incident* OR conduct OR act OR abus* OR vilif* OR language* OR violen* OR rape* OR murder* OR harass* OR terroris* OR narrative* OR discourse* OR propaganda OR “targeted violence” OR incite* OR extremis* OR hostil* OR micro-aggression or microaggression OR group*)) |
| #41 | TS=(misogyn* NEAR/2 (crim* OR speech OR incident* OR conduct OR act OR abus* OR vilif* OR language* OR violen* OR rape* OR murder* OR harass* OR terroris* OR narrative* OR discourse* OR propaganda OR “targeted violence” OR incite* OR extremis* OR hostil* OR micro-aggression or microaggression OR group*)) |
| #42 | TS=(misandr* NEAR/2 (crim* OR speech OR incident* OR conduct OR act OR abus* OR vilif* OR language* OR violen* OR rape* OR murder* OR harass* OR terroris* OR narrative* OR discourse* OR propaganda OR “targeted violence” OR incite* OR extremis* OR hostil* OR micro-aggression or microaggression OR group*)) |
| #43 | TS=(gender-based NEAR/2 (crim* OR speech OR incident* OR conduct OR act OR abus* OR vilif* OR language* OR violen* OR rape* OR murder* OR harass* OR terroris* OR narrative* OR discourse* OR propaganda OR “targeted violence” OR incite* OR extremis* OR hostil* OR micro-aggression or microaggression OR group*)) |
| #44 | TS=(“gendered” NEAR/2 (crim* OR speech OR incident* OR conduct OR act OR abus* OR vilif* OR language* OR violen* OR rape* OR murder* OR harass* OR terroris* OR narrative* OR discourse* OR propaganda OR “targeted violence” OR incite* OR extremis* OR hostil* OR micro-aggression or microaggression OR group*)) |
| #45 | TS=(incel NEAR/2 (crim* OR speech OR incident* OR conduct OR act OR abus* OR vilif* OR language* OR violen* OR rape* OR murder* OR harass* OR terroris* OR narrative* OR discourse* OR propaganda OR “targeted violence” OR incite* OR extremis* OR hostil* OR micro-aggression or microaggression OR group*)) |
| #46 | TS=(invcel NEAR/2 (crim* OR speech OR incident* OR conduct OR act OR abus* OR vilif* OR language* OR violen* OR rape* OR murder* OR harass* OR terroris* OR narrative* OR discourse* OR propaganda OR “targeted violence” OR incite* OR extremis* OR hostil* OR micro-aggression or microaggression OR group*)) |
| #47 | TS=(“involuntary celibate” NEAR/2 (crim* OR speech OR incident* OR conduct OR act OR abus* OR vilif* OR language* OR violen* OR rape* OR murder* OR harass* OR terroris* OR narrative* OR discourse* OR propaganda OR “targeted violence” OR incite* OR extremis* OR hostil* OR micro-aggression or microaggression OR group*)) |
| #48 | TS=(anti-feminis* NEAR/2 (crim* OR speech OR incident* OR conduct OR act OR abus* OR vilif* OR language* OR violen* OR rape* OR murder* OR harass* OR terroris* OR narrative* OR discourse* OR propaganda OR “targeted violence” OR incite* OR extremis* OR hostil* OR micro-aggression or microaggression OR group*)) |
| #49 | TS=(anti-abortion NEAR/2 (crim* OR speech OR incident* OR conduct OR act OR abus* OR vilif* OR language* OR violen* OR rape* OR murder* OR harass* OR terroris* OR narrative* OR discourse* OR propaganda OR “targeted violence” OR incite* OR extremis* OR hostil* OR micro-aggression or microaggression OR group*)) |
| #50 | TS=(anti-doctor NEAR/2 (crim* OR speech OR incident* OR conduct OR act OR abus* OR vilif* OR language* OR violen* OR rape* OR murder* OR harass* OR terroris* OR narrative* OR discourse* OR propaganda OR “targeted violence” OR incite* OR extremis* OR hostil* OR micro-aggression or microaggression OR group*)) |
| #51 | TS=(“anti-sex worker” NEAR/2 (crim* OR speech OR incident* OR conduct OR act OR abus* OR vilif* OR language* OR violen* OR rape* OR murder* OR harass* OR terroris* OR narrative* OR discourse* OR propaganda OR “targeted violence” OR incite* OR extremis* OR hostil* OR micro-aggression or microaggression OR group*)) |
| #52 | TS=(anti-politician NEAR/2 (crim* OR speech OR incident* OR conduct OR act OR abus* OR vilif* OR language* OR violen* OR rape* OR murder* OR harass* OR terroris* OR narrative* OR discourse* OR propaganda OR “targeted violence” OR incite* OR extremis* OR hostil* OR micro-aggression or microaggression OR group*)) |
| #53 | #1 OR #2 OR #3 OR #4 OR #5 … OR #51 OR #52 |
| #54 | TS=(“United States” OR “US” OR “USA” OR Australia* OR “New Zealand*” OR Aotearoa OR France OR French OR German* OR Irish OR Ireland OR Ital* OR Spain OR Spanish OR “UK” OR “United Kingdom” OR Brit* OR Engl* OR “Northern Ireland” OR “Northern Irish” OR Scot* OR Wales OR Welsh OR Canad*) |
| #55 | #53 AND #54 |

| **Date of search** | **Database** | **Language limiters** | **Date limiters** | **Document type limiters** | **Discipline limiters** | **Number of search results** | **Number of results imported into EndNote** | **Comments** |
| --- | --- | --- | --- | --- | --- | --- | --- | --- |
| 17/03/2022 | Web of Science Core Collection | English, French, German, Italian, Spanish | 1990-2021 | N/A | Architecture, Art, Arts & Humanities Other Topics, Asian Studies, Classics, Dance, Film, Radio & Television, History, History & Philosophy of Science, Literature, Music, Philosophy, Religion, Theater, Anthropology, Behavioral Sciences, Health Care Sciences & Services, Nursing, Psychiatry, Public, Environmental & Occupational Health, Archaeology, Area Studies, Biomedical Social Sciences, Business & Economics, Communication, Criminology & Penology, Cultural Studies, Demography, Development Studies, Education & Educational Research, Ethnic Studies, Family Studies, Geography, Government & Law, International Relations, Linguistics, Mathematical Methods In Social Sciences, Psychology, Public Administration, Social Issues, Social Sciences Other Topics, Social Work, Sociology, Urban Studies, Women's Studies, Computer Science | 5174 | 5175 | To support reference screening by language, we exported references from Web of Science Core Collection grouped by language. The discrepancy between the number of search results retrieved through the search and that imported into EndNote is likely a result of doing so. |
| 17/03/2022 | Web of Science SciELO | English, Italian, Spanish | 1990-2021 | N/A | Architecture, Art, Arts & Humanities Other Topics, Asian Studies, Classics, Dance, Film, Radio & Television, History, History & Philosophy of Science, Literature, Music, Philosophy, Religion, Theater, Anthropology, Behavioral Sciences, Health Care Sciences & Services, Nursing, Psychiatry, Public, Environmental & Occupational Health, Archaeology, Area Studies, Biomedical Social Sciences, Business & Economics, Communication, Criminology & Penology, Cultural Studies, Demography, Development Studies, Education & Educational Research, Ethnic Studies, Family Studies, Geography, Government & Law, International Relations, Linguistics, Mathematical Methods In Social Sciences, Psychology, Public Administration, Social Issues, Social Sciences Other Topics, Social Work, Sociology, Urban Studies, Women's Studies, Computer Science | 122 | 122 |  |

## Scopus

| Search syntax |
| --- |
| TITLE-ABS-KEY(((hate OR prejudice* OR bias* OR racis* OR xenophobi* OR sinophobi* OR anti-foreigner OR anti-migrant* OR anti-immigrant* OR anti-refugee* OR "anti-asylum seeker" OR anti-Roma OR anti-traveller OR anti-Gypsy OR "anti-First Nations" OR anti-Indigenous OR anti-Maori OR anti-Aboriginal OR islamophobi* OR antisemiti* OR anti-semiti* OR anti-Jew* OR anti-Amish OR anti-Sikh OR anti-Buddhis* OR anti-Muslim* OR anti-Islam* OR anti-Christian* OR homophobi* OR transphobi* OR lesbophobi* OR biphobi* OR anti-gay OR anti-lesbian* OR anti-bisex* OR anti-transgender OR anti-LGBT* OR ableis* OR disableis* OR sexis* OR mysogyn* OR misandr* OR gender-based OR "gendered" OR incel OR invcel OR "involuntary celibate" OR anti-feminis* OR anti-abortion OR anti-doctor OR "anti-sex worker" OR anti-politician) W/2 (crim* OR speech OR incident* OR conduct OR act OR abus* OR vilif* OR language* OR violen* OR rape* OR murder* OR harass* OR terroris* OR narrative* OR discourse* OR propaganda OR "targeted violence" OR incite* OR extremis* OR hostil* OR micro-aggression or microaggression OR group*)) AND ("United States" OR "US" OR "USA" OR Australia* OR "New Zealand*" OR Aotearoa OR France OR French OR German* OR Irish OR Ireland OR Ital* OR Spain OR Spanish OR "UK" OR "United Kingdom" OR Brit* OR Engl* OR "Northern Ireland" OR "Northern Irish" OR Scot* OR Wales OR Welsh OR Canad*)) |

| **Date of search** | **Database** | **Language limiters** | **Date limiters** | **Document type limiters** | **Discipline limiters** | **Number of search results** | **Number of results imported into EndNote** | **Comments** |
| --- | --- | --- | --- | --- | --- | --- | --- | --- |
| 17/03/2022 | Scopus | English, French, German, Italian, Spanish | 1990-2021 | N/A | SOCI, ARTS, PSYC, COMP, BUSI, NURS, ECON, HEAL, DECI, MULT | 6,443 | 6,489 | To support reference screening by language, we exported references from Scopus grouped by language. The discrepancy between the number of search results retrieved through the search and that imported into EndNote is likely a result of doing so. |

# Group 2: Databases that allow for simple searches

| Date | Database | URL | Search limiters | Search fields | Search syntax | Number of search results | Number of results imported into EndNote | Comments |
| --- | --- | --- | --- | --- | --- | --- | --- | --- |
| 28/01/2022 | National Criminal Justice Reference System | https://www.ojp.gov/ncjrs/virtual-library | **Published after:** 12/1989  **Published before:** 01/2022 | “General search” | hate crime | 645 |  | We combined search results across all searches in a single Excel spreadsheet and removed 164 duplicates prior to import into EndNote. |
|  |  |  |  |  | hate speech | 39 |  |  |
|  |  |  |  |  | hate incident | 21 |  |  |
|  |  |  |  |  | hate conduct | 1 |  |  |
|  |  |  |  |  | hate propaganda | 1 |  |  |
|  |  |  |  |  | hate group | 97 |  |  |
|  |  |  |  |  | bias crime | 142 |  |  |
|  |  |  |  |  | prejudice-motivated crime | 0 |  |  |
|  |  |  |  |  |  | **Total** | **782** |  |
| 29/01/2022 | UN Digital Library | https://digitallibrary.un.org/ | **Added since:** 01/01/2002  **until:** 31/12/2021 | “All of the words” in “any field” | hate crime | 6 | 1 | We manually entered search results into EndNote. We did not enter references for ineligible document types (press releases, letters) or ineligible countries, and removed 15 duplicates. When we entered “01/01/1990” in the “Added since” field, it automatically adjusted to “01/01/2002”. |
|  |  |  |  |  | hate speech | 28 | 19 |  |
|  |  |  |  |  | hate incident | 0 | 0 |  |
|  |  |  |  |  | hate conduct | 0 | 0 |  |
|  |  |  |  |  | hate propaganda | 3 | 1 |  |
|  |  |  |  |  | hate group | 19 | 0 |  |
|  |  |  |  |  | bias crime | 3 | 3 |  |
|  |  |  |  |  | prejudice-motivated crime | 0 | 0 |  |
|  |  |  |  |  |  | **Total** | 24 |  |
| 29/01/2022 | UN Office of the High Commissioner for Human Rights Digital Library | https://searchlibrary.ohchr.org/ | **Added since:** 01/01/2002  **until:** 31/12/2021 | “All of the words” in “any field” | hate crime | 10 | 6 | We manually entered search results into EndNote. We did not enter references for ineligible document types (press releases, letters), for ineligible countries or in ineligible languages, and removed 23 duplicates. |
|  |  |  |  |  | hate speech | 18 | 14 |  |
|  |  |  |  |  | hate incident | 2 | 2 |  |
|  |  |  |  |  | hate conduct | 0 | 0 |  |
|  |  |  |  |  | hate propaganda | 0 | 0 |  |
|  |  |  |  |  | hate group | 24 | 2 |  |
|  |  |  |  |  | bias crime | 0 | 0 |  |
|  |  |  |  |  | prejudice-motivated crime | 0 | 0 |  |
|  |  |  |  |  |  | **Total** | 24 |  |
| 30/01/2022 | EUR-Lex | https://eur-lex.europa.eu/homepage.html | **Collections:**  Treaties, Legal Acts, International Agreements, National Transposition  **All of these languages:** English, French, German, Italian, Spanish | “All of the words” in “any field” | hate crime | 4 |  | Upon review of search results, we found our search strategy to be unsatisfactory for the purpose of identifying EU legislation. We therefore decided to not consider any search results and have a legal expert conduct a systematic search for relevant legislation. |
|  |  |  |  |  | hate speech | 8 |  |  |
|  |  |  |  |  | hate incident | 0 |  |  |
|  |  |  |  |  | hate conduct | 0 |  |  |
|  |  |  |  |  | hate propaganda | 0 |  |  |
|  |  |  |  |  | hate group | 0 |  |  |
|  |  |  |  |  | bias crime | 2 |  |  |
|  |  |  |  |  | prejudice-motivated crime | 0 |  |  |
|  |  |  |  |  |  | **Total** | **0** |  |
| 31/01/2022 | EU Publications Office | https://op.europa.eu/en/home | **Collections:** EU Publications  **Language:** English, French, German, Italian, Spanish  **from:** 01/01/1990  **to:** 31/12/2021 | **Find results with** “Exact phrase” | hate crime | 505 |  | We combined search results across all searches in a single Excel spreadsheet. We removed 157 duplicates prior to import into EndNote. We furthermore manually identified various series of reports (e.g., EASO country of origin reports, country reports on implementation of non-discrimination principles) and removed 263 references because they were country reports for countries not eligible for inclusion in our review. We retained all reports from these report series for countries eligible for inclusion, including in cases where it was not clear whether the report would focus on an eligible country. |
|  |  |  |  |  | hate speech | 895 |  |  |
|  |  |  |  |  | hate incident | 4 |  |  |
|  |  |  |  |  | hate conduct | 2 |  |  |
|  |  |  |  |  | hate propaganda | 9 |  |  |
|  |  |  |  |  | hate group | 9 |  |  |
|  |  |  |  |  | bias crime | 10 |  |  |
|  |  |  |  |  | prejudice-motivated crime | 0 |  |  |
|  |  |  |  |  |  | **Total** | **1,031** |  |
| 31/01/2022 | OSCE Office for Democratic Institutions and Human Rights Document Library | https://www.osce.org/resources/documents | **Institutions & Structures:** OSCE Office for Democratic Institutions and Human Rights  **Year of publishing:** (from year) 1990 (to year) 2021  **Document type:** Conference/Meeting Document, Recommendation, Report, Treaty/Agreement | “Enter your keywords:” | hate crime | 1,003 |  | We performed the searches without applying the filter “Document type” and combined search results across all searches in a single Excel spreadsheet. We included the classification of the document type for each reference in the Excel spreadsheet. In a first step, we filtered the search results in the Excel spreadsheet to only include eligible document types. Thereby, we removed a total of 1,966 search results. In a second step, we removed 2,429 duplicates prior to import into EndNote. |
|  |  |  |  |  | hate speech | 1,000 |  |  |
|  |  |  |  |  | hate incident | 758 |  |  |
|  |  |  |  |  | hate conduct | 750 |  |  |
|  |  |  |  |  | hate propaganda | 203 |  |  |
|  |  |  |  |  | hate group | 963 |  |  |
|  |  |  |  |  | bias crime | 1 |  |  |
|  |  |  |  |  | prejudice-motivated crime | 479 |  |  |
|  |  |  |  |  |  | **Total** | 762 |  |
| 08/03/2022 | Google Scholar | https://scholar.google.com/ | **Language:** English  **Custom range:**  1990-2021 |  | “hate crime”\|“hate speech”\|“hate incident”\|“hate conduct”\|“hate propaganda”\|“hate group”\|“prejudice-motivated crime”\|“bias crime” | 23,900 | 200 | As per our Protocol, we retrieved the first 200 search results. |
|  |  |  |  |  |  | **Total** | 200 |  |
| 08/03/2022 | Google Scholar | https://scholar.google.com/ | **Language:** French  **Custom range:**  1990-2021 |  | “crime de haine”\|“discours haineux”\|“incident de haine”\|“conduite haineuse”\|“groupe de haine”\|“crime motivé par des préjugés”\|“crime biaisé” | 1,120 | 200 | As per our Protocol, we retrieved the first 200 search results. |
|  |  |  |  |  |  | **Total** | 200 |  |
| 15/03/2022 | Google Scholar | https://scholar.google.com/ | **Language:** German  **Custom range:**  1990-2021 |  | “Hasskriminalität”\|“Hassrede”\|“Hassvorfall”\|“Hassverhalten”\|“Hassgruppe”\|“Hasspropaganda”\|“vorurteilsmotivierte Straftat”\|“Vorurteilskriminalität” | 2,730 | 200 | As per our Protocol, we retrieved the first 200 search results. |
|  |  |  |  |  |  | **Total** | 200 |  |
| 15/03/2022 | Google Scholar | https://scholar.google.com/ | **Language:** Italian  **Custom range:**  1990-2021 |  | “crimini d’odio”\|“discorsi d’odio”\|“incidenti di odio”\|“comportamento di odio”\|“propaganda d’odio”\|“gruppi d’odio”\| “crimini motivati da pregiudizio” | 496 | 200 | As per our Protocol, we retrieved the first 200 search results. |
|  |  |  |  |  |  | **Total** | 200 |  |
| 15/03/2022 | Google Scholar | https://scholar.google.com/ | **Language:** Spanish  **Custom range:**  1990-2021 |  | “crimen de odio”\|“discurso de odio”\|“incidente de odio”\|“conducta de odio”\|“propaganda de odio”\|“grupo de odio”\|“crimen motivado por prejuicio” | 3,390 | 200 | As per our Protocol, we retrieved the first 200 search results. |
|  |  |  |  |  |  | **Total** | 200 |  |
| 05/04/2022 | ScienceDirect | https://www.sciencedirect.com/ | **Year(s):**  1990-2021 | “Find article with these terms” | “hate crime” OR “hate speech” OR “hate incident” OR “hate conduct” OR “hate propaganda” OR “hate group” OR “prejudice-motivated crime” OR “bias crime” | 155 | 155 |  |
|  |  |  |  |  |  | **Total** | 155 |  |
